# Network pharmacology-based identifcation of potential targets of the flower of *Trollius chinensis Bunge* acting on anti-inflammatory effectss

**DOI:** 10.1038/s41598-019-44538-z

**Published:** 2019-05-30

**Authors:** Jing-wei Liang, Ming-yang Wang, Kamara Mohamed Olounfeh, Nan Zhao, Shan Wang, Fan-hao Meng

**Affiliations:** School of Pharmacy, China Medical University, Liaoning, 110122 China

**Keywords:** Computational biology and bioinformatics, Immunological disorders

## Abstract

The flower of *Trollius chinensis Bunge* was widely used for the treatment of inflammation-related diseases in traditional Chinese medicine (TCM). In order to clarify the anti-inflammatory mechanism of this Chinese herbs, a comprehensive network pharmacology strategy that consists of three sequential modules (pharmacophore matching, enrichment analysis and molecular docking.) was carried out. As a result, Apoptosis signal-regulating kinase 1 (ASK1), Janus kinase 1 (JAK1), c-Jun N-terminal kinases (JNKs), transforming protein p21 (HRas) and mitogen-activated protein kinase 14 (p38α) that related to the anti-inflammatory effect were filtered out. In further molecular dynamics (MD) simulation, the conformation of CID21578038 and CID20055288 were found stable in the protein ASK1 and JNKs respectively. The current investigation revealed that two effective compounds in the flower of *Trollius chinensis Bunge* played a crucial role in the process of inflammation by targeting ASK1 and JNKs, the comprehensive strategy can serve as a universal method to guide in illuminating the mechanism of the prescription of traditional Chinese medicine by identifying the pathways or targets.

## Introduction

The flower of *Trollius chinensis Bunge* was a Chinese herb for the treatment of inflammation-related diseases such as tract infection, tonsillitis, and pharyngitis that caused by toxic heat^1^. In traditional Chinese medicine (TCM), it has a long history of medicinal use^2^. “The flower of *Trollius chinensis Bunge*, properties: bitter, cold, non-toxic, treat aphtha, sore throat, otalgia and ophthalmalgia”, as recorded in “Supplement to compendium of Materia Medica”^3^. Modern with advanced technology applied it to medicine or tea such as Trollii Flos Oral Liquid, played a superb effect in the aspect of anti-inflammatory and cleaning away heat and toxic material^4–6^. So it would be interesting and significative to study the molecular mechanism of the treatment of inflammation by effective compounds in the Chinese herb. The past researches have shown that the natural products in the Chinese herb mainly contains flavonoids, phenolic acids, and alkaloids, which had demonstrated biological activity in past researches^7–10^. Most studies focused on the relationship between single component and its anti-inflammatory activity^6,11^, but no one revealed the complex interactions between the Chinese herb and cellular proteins, or detect the influence of their interactions on the function and behavior of human body.

Network pharmacology was becoming an easily available method with rapid growth of available biomedical data in the post genomic era, systems biology and poly pharmacology^12^. The holistic philosophy of TCM has much in common with the key ideas of network pharmacology, which shifts the “one target, one drug” paradigm to the “network target, multi-component” strategy^13,14^. Subsequently, a set of TCM network pharmacology methods was created for sequencing disease-related genes, predicting the targeting distribution and pharmacological effects of herbal compounds, revealing the common modular association of “drug-target-pathway-disease”, and screening synergistic compounds in TCM formulations efficiently^15–17^.

**Figure 1:**
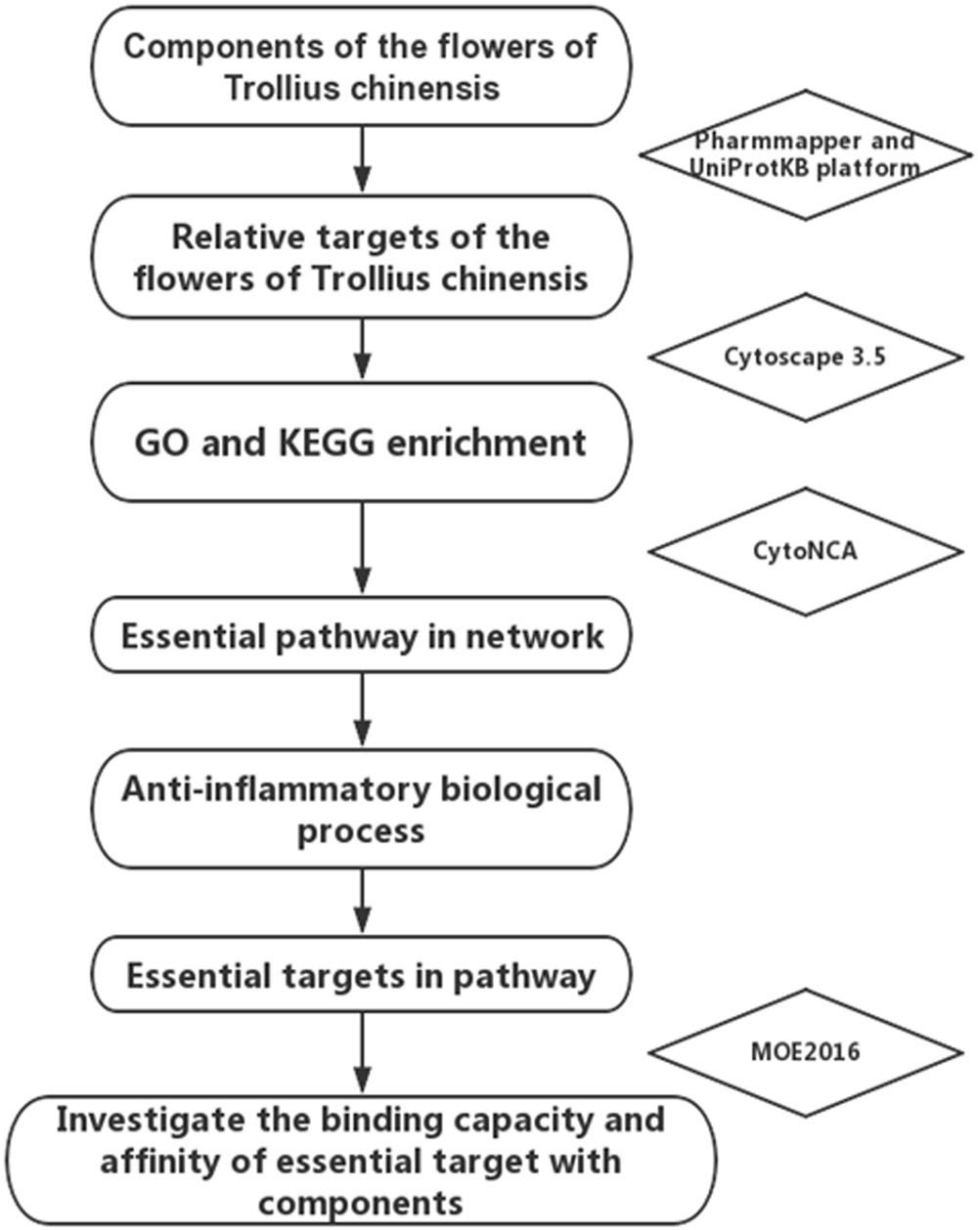
The integrated process of the Network pharmacology based method to identifying the anti-inflammatory mechanism of the flower of *Trollius chinensis Bunge*.

The effects produced by the flower of *Trollius chinensis Bunge* cannot be adequately explained by considering separately each ingredient in it, so it was necessary for applying the TCM network pharmacology method to illuminate the anti-inflammatory mechanism of this Chinese herb. In the current investigation, after collecting compounds gathered from the past literatures, a comprehensive network pharmacology strategy was carried out. The strategy was composed with three sequential modules: predicting potential target by the reverse docking method, enrichment analysis according to GO terms and KEGG, docking and molecular dynamics technology for investigating the binding affinity and stability of ligand-receptor conformation. The integrated process was showed in Fig. 1.

## Method

### Data preparation and construction

The chemical structure information of effective components in Trollius chinensis was gathered from relevant literature^4,6–8,10^. Finally, a total of twenty compounds which include flavonoids, phenolic acids, and alkaloids were gathered in Table 1. All the compounds were downloaded from the ChemSpider (http://www.chemspider.com/) and NCBI PubChem database (http://www.ncbi.nlm.nih.gov/pccompound/) and saved as mol2 format. Then the charges of the compounds were calculated by the Gasteiger-Huckel method, and tripos force field was utilized for energy minimization of them in MOE2016.

**Table 1:** Twenty effective components of the flowers of Trollius chinensis.

No.	PubChem CID	Compound name
01	44258343	Isoswertisin 2″-O-(2‴-methylbutyrate)
02	21578038	22″-O-(3‴,4‴-Dimethoxybenzoyl)vitexin
03	5280442	Acacetin
04	44257884	acacetin-7-O-β-D-glucoside
05	20055288	apigenin-8-C-(2-O-feruloyl)-β-D-glucoside
06	159460	cirsimarin
07	188323	cirsimaritin
08	5281416	esculetin
09	101606458	ICARISIDE b
10	5280804	isoquercetin
11	5281675	orientin
12	5320438	pectolinarigenin
13	486614	pinoresinol-β-D-glucopyranoside
14	132759	progloberflowery acid
15	72	protocatechuic acid
16	5280343	quercetin
17	11160309	trolline
18	4483040	trollioside
19	7121	veratric acid
20	5280441	vitexin

The Pharmmapper server is an updated integrated pharmacophore matching platform with statistical method and reverse docking technology for identifying the potential targets of the natural products and the synthetic compounds^18,19^. The mol2 format files of the twenty compounds were submitted to the Pharmmapper server, the Conformation Generation parameters of Generate Confomers and Maximum Generated Conformations was set as ON and 300 respectively. In advanced options, the Druggable Pharmacophore Models (v2017, 16159) was selected as targets set 15, other options were used as the default. Finally, the top 300 reserved matched targets were selected for the following study of the comprehensive network pharmacology analysis, according to the receiver operating characteristic (ROC) curve which depicted the fraction of true positives versus the fraction of false positives^20^.

### Network construction and enrichment analysis

After overlapping the results of the twenty compounds that received from the Pharmmapper service, the one that occurs more than once were converted to the UniProtKB identifiers by employing the Retrieve/ID mapping (http://www.uniprot.org/uploadlists/) for the following enrichment analysis.

Cytoscape is an open source software platform for visualizing molecular interaction networks and biological pathways, then integrating these networks with annotations, gene expression profiles and other state data. The putative components-target network were constructed by generating the linkage that exists between the 20 compounds and the result of pharmmapper in Cytoscape software^3^, the network contain a verity of functional nodes and edges. In order to biological interpretation of large lists of putative target, the GO and KEGG enrichment analysis of components-target network were carried out for identifying the key pathway of the anti-inflammation effect of the title flower^21,22^.

The ClueGO plugins in Cytoscape 3.5 software were used to perform KEGG and GO enrichment analysis. ClueGO is a user-friendly Cytoscape plugin to analyze interrelations of terms and functional groups in biological networks, a variety of flexible adjustments allow for a profound exploration of gene clusters in annotation networks of the key GO term^23,24^. In operation interface, all the pathways and targets related to respiratory inflammation will be struck out in the list of results. In ClueGO operation interface, the compound nodes were deleted and others UniprotKB maker nodes with relative edges were put into Homo sapiens load list. The Go tree interval with values range from 5 to 10, kappa score was set as 0.4, p values threshold was set as less 0.01, the result of ClueGO only show the Biological Process terms and KEGG pathway with the p values less than or equal 0.01.

In order to screen the essential genes and pathways in the complicated enrichment network, the CytoNCA plugin was carried out to calculate the centrality measures of nodes. CytoNCA is a user-friendly and multifunctional Cytoscape plugin, it offers eight centrality measures (Betweenness, Closeness, Degree Centrality, Eigenvector Centrality, Local Average Connectivity-based Centrality, Network Centrality, Subgraph Centrality and Information Centrality), various visualization analyses, and comprehensive construction of essential interaction sub-networks^25^. We selected top 10% nodes to the Betweenness, Subgraph and Closeness Centrality measures respectively, then generated the sub-network with essential genes by overlapping the three centrality measures groups.

### Molecular docking and dynamics

Some proteins that screened out by the Pharmmapper server existed in various biological processes and signaling pathways, but had weak binding with the compounds. Similarly, some compounds in the flower probably had weak binding to key proteins. In order to weed out these proteins and compounds, virtual screening based on molecular docking was carried out.

Molecular Operating Environment (MOE) was a professional molecular simulation software which cover the functions of ADME (absorption, distribution, metabolizm and excretion), QSAR (quantitative structure-activity relationship), homology modeling, pharmacophore models, molecular docking and molecular dynamics, these functions provide a computational method to researcher in drug Discovery and biochemistry. The molecular docking and dynamics technology in MOE 2016 software were carried out to investigate the binding affinity of the putative target to the compound. Twenty ligands were from Method 1, and the target proteins from KEGG pathway were downloaded from RCSB Protein Data Bank (http://www.rcsb.org/pdb/home/home.do), the downloaded protein pdb data was optimized by Quickprep and Protonate 3D function in Amber10 EHT force field and Gasteiger-Huckel charges for further docking. In docking operation interface, the placement and refinement were set as “Triangle Matcher” and “Induced Fit” respectively, the London dG score will be used to evaluate ligand binding capacity to receptor^26,27^.

In order to evaluate the stability of docking conformations, molecular dynamics studies were carried out. The molecular dynamics simulation in MOE2016 was carried out for verifying the stability and affinity of the ligand-receptor complexes. The force field and charges parameters in energy minimization process conform to the parameters of above docking study. In preparation step, the ligand-receptor complexes was put into a 16 Å^3^ size water box (Periodic boundary condition, PBC). There are three main step in molecular dynamics simulation: restrictive MD and Non-restrictive MD. In restrictive MD process, the backbone in canonical ensemble (NVT) system was restrained with the Constrain parameter of “Tether”, under the restrictive MD, the initial temperature 300 K was gradually increasing to biological temperature 310 K in 100 ps^28,29^. Then in Non-restrictive MD process, the Constrain parameter was set to “Free”, the root mean square deviations (RMSD) of the receptor-ligand complex will be analyzed in a 5 ns MD simulations (with 2 fs timestep).

## Result and Discussion

### Data preparation and construction

**Figure 2:**
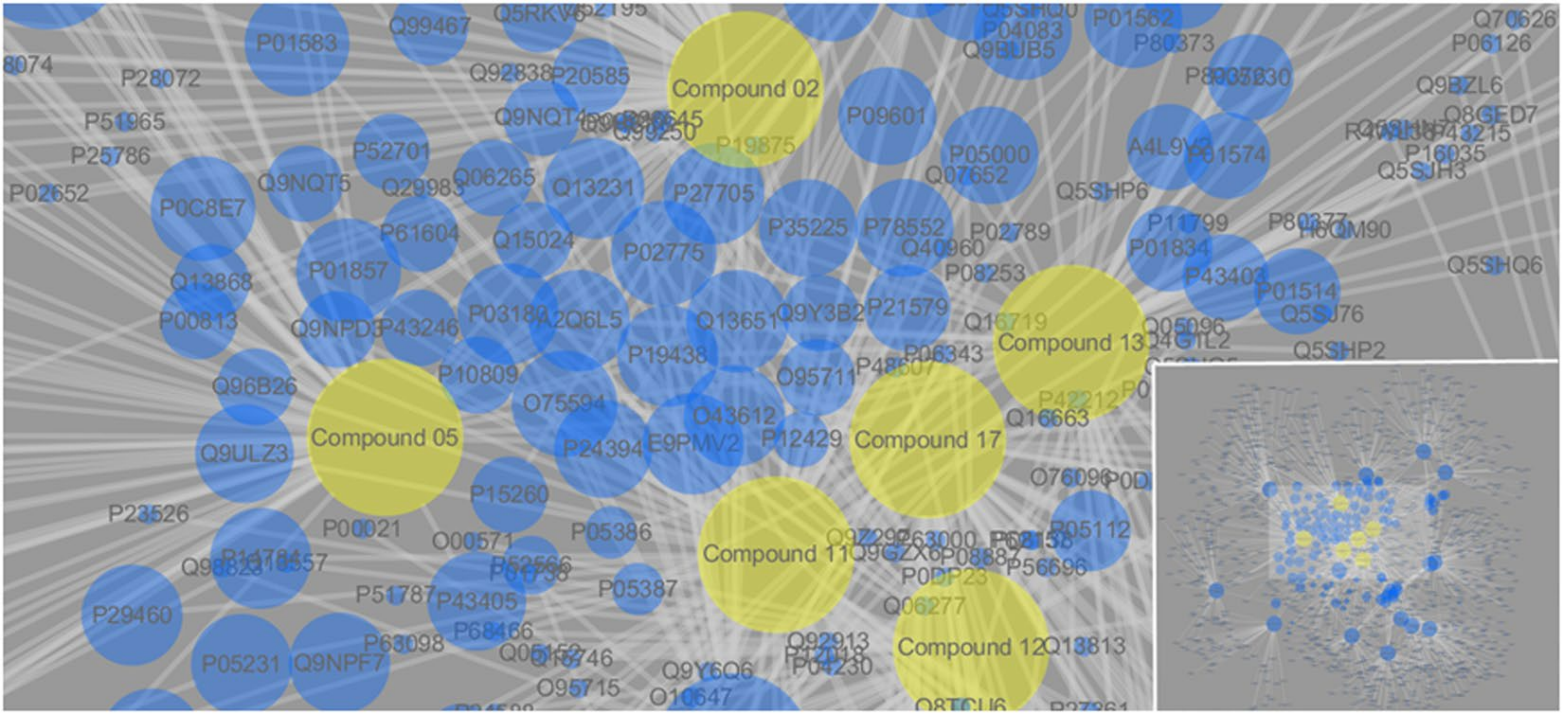
The network of the 20 compounds of the title flower of and putative target from Pharmmapper result. The compound 2, 5, 11, 12, 13 and 17 in the central moiety of the network.

The 6000 target proteins were received from the Pharmmapper service. After overlapping, 2579 protein targets were converted to 1258 UniProtKB identifiers by employing the Retrieve/ID mapping (http://www.uniprot.org/uploadlists/) for the following enrichment analysis (in Table S1). The 1258 UniProtKB identifiers of the twenty compounds were collected as listed in Table S2. The predictive interactions were used to build the compound-target network in Cytoscape software. After converting compounds and targets to nodes, the generated network contained 1278 nodes and 1855 edges were shown in Fig. 2. The different sizes of the target nodes indicated that edges have connectivity which represented the degree of compounds with the common target protein. The compound 2, 5, 11, 12, 13 and 17 in the central moiety of the network demonstrated more multiple interactions with others UniProtKB nodes than others in periphery moiety. The central UniProtKB nodes mainly includes interleukin (Such as IL12B, IL1A and IL13 with UniProtKB P29460, P01538 and P35225 respectively), platelet basic protein(PPBP with UniProtKB P02775), Immunoglobulin (IGHG with UniProtKB P01587), Tumor necrosis factor receptor superfamily member (TNFRSF1A with UniProtKB P19438), these proteins are all involved in immune and inflammatory response. The network revealed that twenty components of the title flower have combined effect for protein target or biological pathway.

### Network construction and enrichment analysis

**Figure 3:**
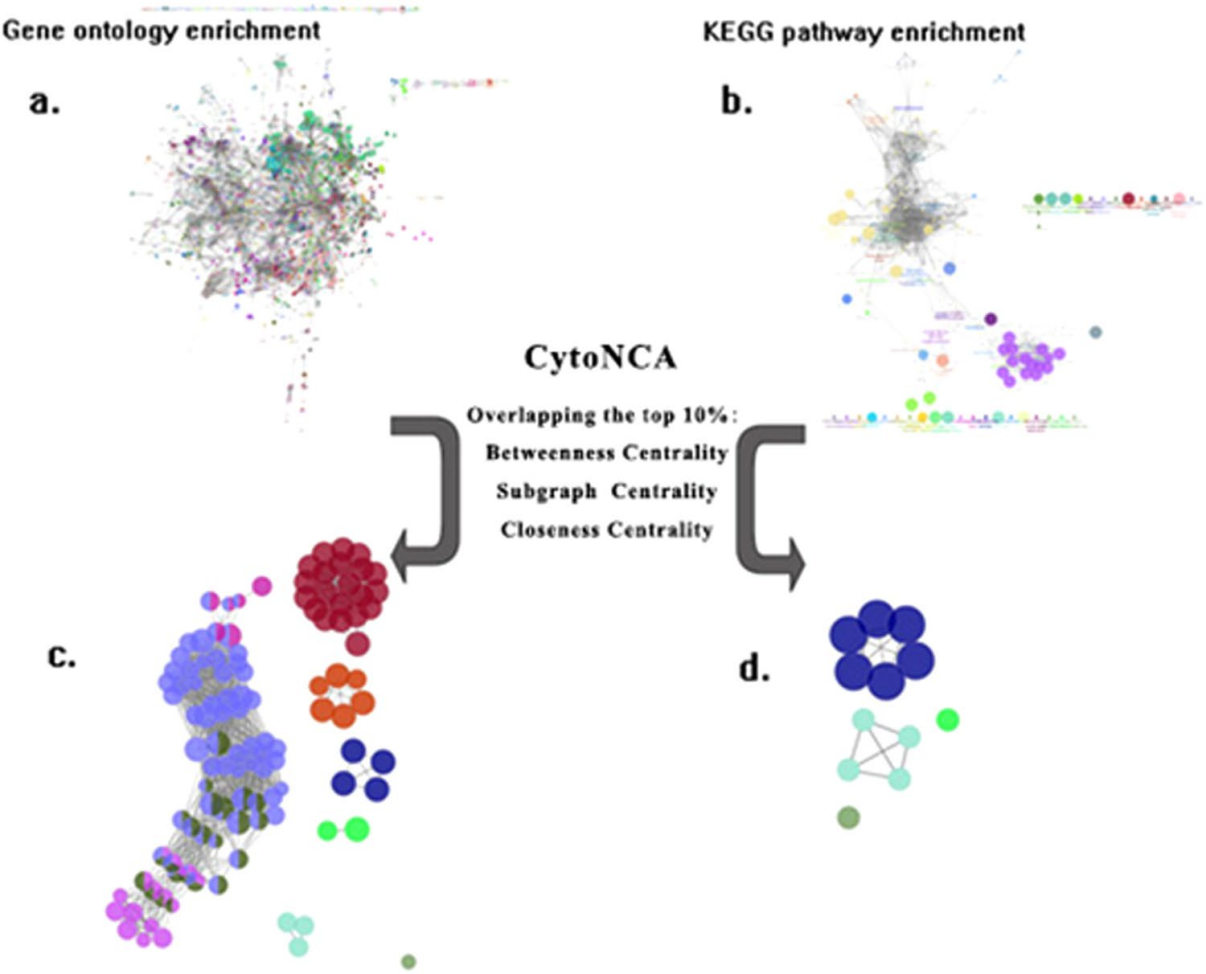
Integrated enrichment network of biological process (BP) gene ontology (**a**) and KEGG pathway (**b**) and sub-network of gene ontology (**c**) and KEGG pathway (**d**).

After neglecting the compounds node in the compound-target network, other nodes with different numbers of edges were used for Gene and KEGG enrichment analysis. Under the setting of parameters in Method 2, ClueGo plugin got two integrated Gene (with 4517 nodes and 166053 edges respectively) and KEGG (with 751 nodes and 5171 edges respectively in) enrichment network were showed in Fig. 3a,b respectively. After overlapping 3 centrality measures groups of nodes, the gene and KEGG sub-network with essential nodes were showed in Fig. 3c,d, the gene terms closely relative were clustered in the same color group.

Chinese herbal medicine is a multi-component compound preparation. In the process of treating diseases, its multi-components will cooperate with each other, similar to the multi-target of single-component drugs^30^. In current research, different components of the flower of *Trollius chinensis Bunge* were found to target different nodes of an interconnected biological network, which performed similar functions. The advantage of acting on an interrelated biological network was avoiding drug resistance caused by bypass activation^31,32^.

In gene enrichment sub-network (with 162 nodes and 1889 edges), 149 gene ontology terms were significantly associated with a great deal of biological processes, including the regulation of IL (interleukin)-2, 4 and 6, cellular response to interferon-gamma, the activation of B and T cell, cellular response to virus, respiratory burst, rRNA and tRNA catabolic process and leukocyte apoptotic process (in Table S3), the representative term with the lowest p values in each groups was documented in Table 2. And the KEGG pathway sub-network contained 12 nodes and 49 edges, associated with various biological processes, such as the cytokine-cytokine receptor interaction, B and T cells receptor signaling pathway, leukocyte transendothelial migration and several signaling pathways, besides, the KEGG pathway sub-network reveal the relationship to respiratory disease pertussis and asthma (in Tables 3 and S3). We found that vast majority of gene ontologies and KEGG pathway were concerned with body immune system, given that the immune responses were accompanied by inflammatory response inevitably, so it is feasible to employ this subnetwork for analyzing the mechanism of the anti-inflammatory effect of the flower of *Trollius chinensis Bunge*.

**Table 2:** The representative gene ontology term with lowest p value of each groups.

Gene Ontology terms	Color in Fig. 3c	p-value
GO:0016078: tRNA catabolic process	Red	2.74 × 10^−22^
GO:0032675: regulation of interleukin-6 production	Mazarine	3.97 × 10^−9^
GO:0098586: cellular response to virus	Green	4.7 × 10^−4^
GO: 0038110: interleukin-2-mediated signaling pathway	Cyan	1.3 × 10^−6^
GO:0045730: respiratory burst	Purple	4.06 × 10^−6^
GO:0060333: interferon-gamma-mediated signaling pathway	Orange	1.33 × 10^−8^
GO:0043368: positive T cell selection	Blue	7.73 × 10^−9^
GO:0002335: mature B cell differentiation	Pink	3.1 × 10^−5^
GO:0071353: cellular response to interleukin-4	Emerald	9.84 × 10^−7^
GO:0070661: leukocyte proliferation	Olive	7.73 × 10^−9^

**Table 3:** The KEGG pathway in enrichment network.

KEGG pathway	Color in Fig. 3d	p-value
hsa04668 TNF signaling pathway	Cyan	1.0 × 10^−15^
hsa04662: B cell receptor signaling pathway	Mazarine	5.8 × 10^−12^
hsa04670: Leukocyte transendothelial migration	Mazarine	5.6 × 10^−10^
hsa04660: T cell receptor signaling pathway	Mazarine	9.6 × 10^−9^
hsa04612: Antigen processing and presentation	Mazarine	1.8 × 10^−8^
hsa04060: Cytokine-cytokine receptor interaction	Mazarine	2.9 × 10^−8^
hsa04010: MAPK signaling pathway	Cyan	1.2 × 10^−6^
hsa04650: Natural killer cell mediated cytotoxicity	Mazarine	2.7 × 10^−6^
hsa04064: NF-κ B signaling pathway	Cyan	3.6 × 10^−3^
hsa04151: PI3K-Akt signaling pathway	Cyan	7.9 × 10^−3^
hsa05133: Pertussis	Emerald	0.00387
hsa05310: asthma	Green	0.00755

In the result of gene ontology and KEGG pathway enrichment analysis, a large quantities of essential genes were related to T cell and B cell receptor signaling pathway or biological processes (the nodes were painted in blue and pink in Fig. 3c, nodes were painted in mazarine in Fig. 3d), indicated that the anti-inflammatory activity of the flower of *Trollius chinensis Bunge* was probably related to the activation of B cell and T cell.

**Figure 4:**
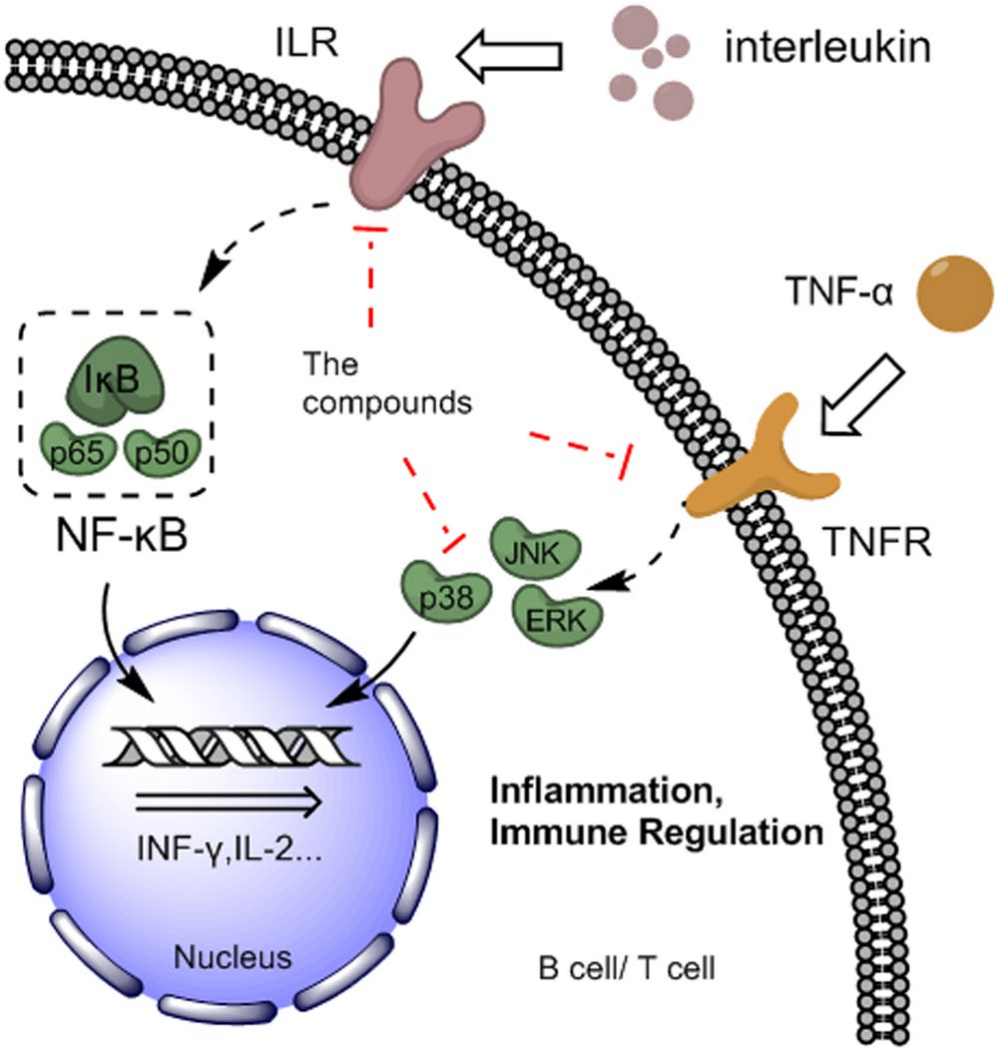
Pharmacological mechanisms cascade pathway of the flower of *Trollius chinensis Bunge* impact on inflammation.

The B cell and T cell-related biological processes contained several pathways. In the process of T cell and B cell participation in inflammation and immune regulation, MAPK and NF-κB pathway played a crucial role in signal transduction of TNF-α and interleukin as demonstrated in Fig. 4 ^33,34^. The interleukin-2 (IL-2), IL-4 and IL-6 signaling pathway were showed as color emerald, cyan and mazarine in Fig. 3c. Like the MAPK and NF-κB pathway, the three interleukins have the effect of promoting the development and differentiation of T and B cell but at an upstream position: IL-2 is a lymphokine that induces the proliferation of responsive T cells, IL4 is produced by CD4+ T cells specialized in providing help to B cells to proliferate and to undergo class switch recombination and somatic hypermutation, IL6 is a cytokine involved in a wide variety of biological functions, which played an essential role in the final differentiation of B cells into immunoglobulin-secreting cells^35,36^. This was consistent with the results of the KEGG pathway enrichment network, as the pathways mentioned above appear in the KEGG results that marked with color cyan.

The olive and purple nodes in Fig. 3c demonstrated many interactions with the nodes of leukocyte proliferation and respiratory burst respectively. Leukocyte produced and derived from multipotent cells in the bone marrow known as hematopoietic stem cells and it was the important cell to prevent the body from both infectious disease and foreign invaders^37,38^. Respiratory burst refers to the cells in immune system have the behavior of releasing reactive oxygen species (ROS), NADPH oxidase and myeloperoxidase were utilized by neutrophils, monocytes and immune cells for combating infections^39^, these were accord with the description about the curative effect of the flower of *Trollius chinensis Bunge* in TCM.

It was noteworthy that biological processes GO:0016078 contained fewer proteins and almost all came from the same family. Cytochrome c-oxidoreductase (Gene ID: UQCR) contributed to the excellent enrichment results of GO:0016078, while the tRNA catabolic process was not associated with inflammation or immune system directly, so this gene ontology terms were undesirable.

In KEGG sub-network, the TNF, MAPK, NF-κB and PI3K-Akt signaling pathways were further divided and placed into the same group in Fig. 3d, TNF-α was a cell factor that involved in systemic inflammation through key proteins in the MAPK pathway^40,41^. In the results of KEGG pathway analysis, the TNF signaling pathway (Entry: hsa04668 and was painted in cyan in Fig. 3d) got the highest KEGG enrichment score among the others pathway, and the TNF regulation related gene ontology appeared simultaneously in the list of gene enrichment analysis. PI3K-Akt signaling pathway (Entry: hsa04151 was painted cyan in Fig. 3d) is also judged as an essential pathway in the KEGG enrichment analysis, because quite a few signaling molecules in the pathway were thought to be crucial in regulating Bad activities, caspase-9, NF-κB, IKK, Raf, and ERK activities, and PI3K-Akt was the downstream signaling pathways in the TNF signaling pathway^42–44^. It was reasonable to conjecture that the compounds regulated the immune response and reduce inflammation by influencing the pathways mentioned above.

In summary, the T cells and B cells biological processes played a crucial role in immune regulation and inflammation, while the TNF, MAPK, NF-κB and PI3K-Akt signaling pathways were the key to the biological processes. After overlapping the results of KEGG sub-network (Table S3), the proteins screened out by the Pharmmapper Server and enriched in the above signaling pathways were selected as targets for further docking research: MAP3K5 (ASK1, PDBID: 4BIB), JAK (JAK1, PDBID: 4EHZ), MAPK10 (JNKs, PDBID: 3V6S), HRAS1 (HRas, PDBID: 4NYI) and MAPK14 (PDBID: 3FLW). So it suggested that the compounds regulated the TNF, MAPK, NF-κB and PI3K-Akt signaling pathways by targeting these key proteins, which affect the differentiation and maturation of T cells and B cells, and then exhibited anti-inflammatory effects.

### Molecular docking and dynamics

The dock results of 20 compounds in the title flower were showed in Table S4, the mean docking scores of the five target proteins(3FLW, 3V6S, 4BIB, 4EHZ, and 4NYI) were 6.87, 7.33, 7.24, 7.25 and 7.01 respectively, demonstrating a similar binding capacity compare with the previous build-in ligands (with the fitscore of 7.06, 7.36, 7.02, 7.57 and 7.37 respectively). The dock results demonstrated that the interaction between the five crucial protein targets in the four KEGG pathways (TNF signaling pathway, NF-κB pathway, MAPK pathway and PIK3-Akt) and flower of *Trollius chinensis Bunge* is beneficial for the anti-inflammatory properties.

For screening out the compounds that possess a better combination with these key proteins, the affinity and binding capacity between the compounds and targets were examed. The compounds-targets complex were screened out in anti-inflammatory cascade response by sorting the docking scores in descending order. Finally, the top 10 docking results screened out from Table S4, the compound 2 (2″-O-(3′,4,-dimethoxybenzoyl)vitexin) and compound 5 (apigenin-8-C-(2-O-feruloyl)-β-D-glucoside) exhibited strong binding capacity to the protein ASK1 and JNKs. Compound 5 is a derivative of apigenin, several past studies have reported that the anti-inflammatory action of apigenin and its glycosylation^44–46^. Compound 2 is a derivative of vitexin, which has been proved to be effective for alleviating inflammation through NF-κB, and p38 signaling pathway^47,48^. ASK1 serve as an a connecting link between the upstream and the downstream member in TNF, MAPK, NF-κB and PI3K pathway, JNKs and p38 can be activated by ASK1 in case of an array of stresses such as cytokines, oxidative stress, endoplasmic reticulum stress and calcium influx. JNKs can bind and phosphorylate c-Jun on Ser-63 and Ser-73 within its transcriptional activation domain, it is suggested that this signaling cascade contributes to inflammatory responses. Compound 5-3v6s and compound 2-4bib (with the fitscores of 10.34 and 10.30 respectively) were selected for further docking analysis and molecular dynamics for examining the affinity and binding capacity.

**Figure 5:**
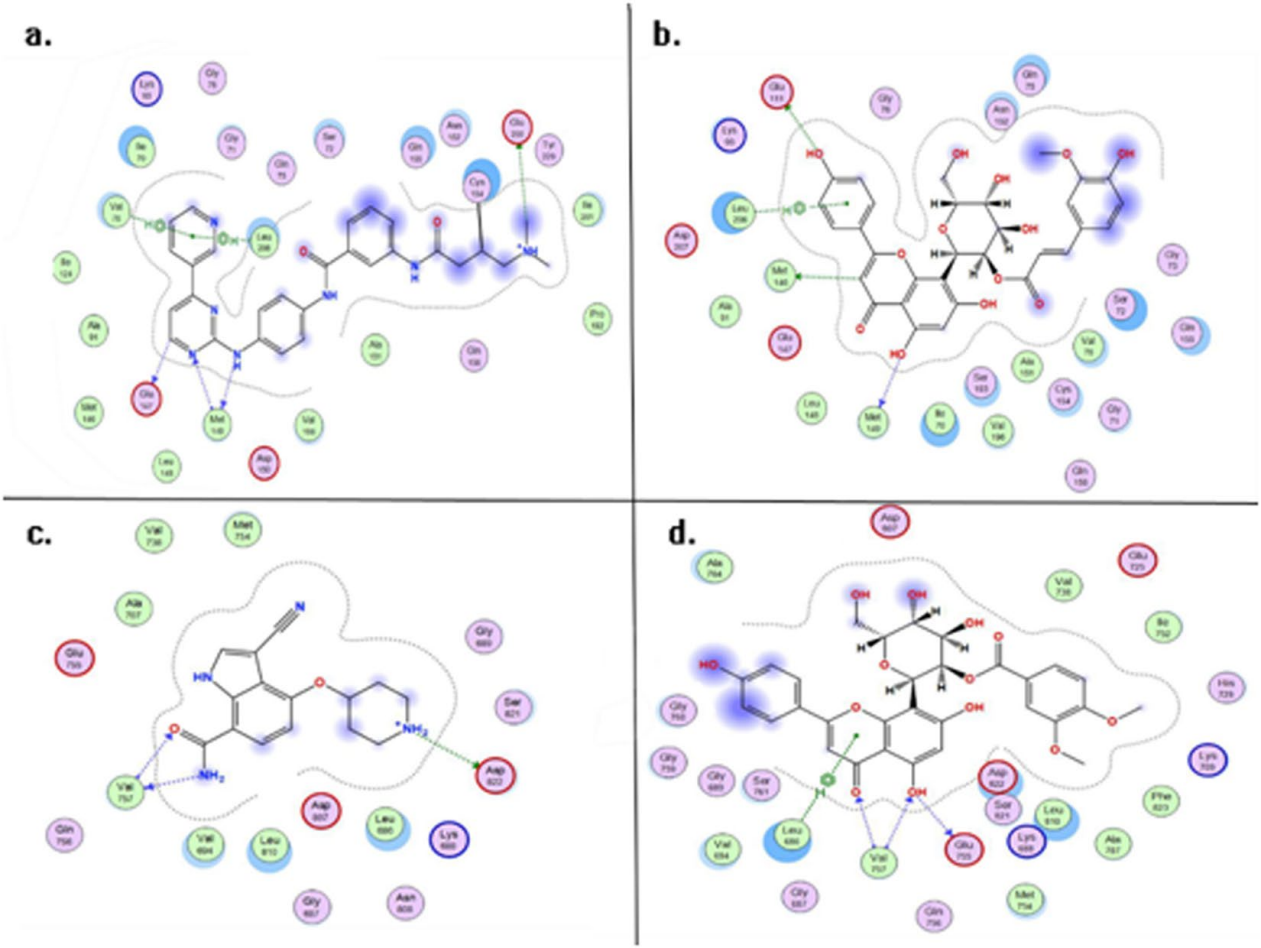
The dock results of inhibitor-3v6s (**a**), compound5-3v6s (**b**), inhibitor-4bib (**c**), and compound2-4bib (**d**).

The ligand interaction of compound 5-3V6S and compound 2-4BIB were showed in Fig. 5b,d respectively. In Fig. 5a, the build-in ligand is a covalent inhibitors of JNKs which inhibits the phosphorylation of c-Jun^49^, the pyridine moiety of build-in ligand formed two arene-H interactions with Val78 and Leu206, such arene-H interaction can be seen between the phenolic group of compound 5 and Leu206 in Fig. 5b. Met149 formed a hydrogen with the pyrimidine of build-in ligand as a backbone acceptor, it exhibit similar interaction to the hydroxyl in compound 5-3V6S. Furthermore, the greasy (Ala91, Ala151 and Leu148), basic (Lys93) and acidic (Glu147) amino acid surround the two ligand were nearly identical, indicating that compound 5 and build-in ligand possess similarly inhibitory effect to JNKs. In Fig. 5c,d, the ASK1 inhibitor and compound 2 formed similar interaction to the surrounding amino acids in active pocket, such as the backbone donor and acceptor interaction of Val757 and the surrounding greasy (Val694, Val738 and Ala707), basic (Lys688) and acidic (Glu755, Asp822 and Asp 807) amino acids, compound 2 may exhibit the same effect through these surrounding acidic amino acids.

**Figure 6:**
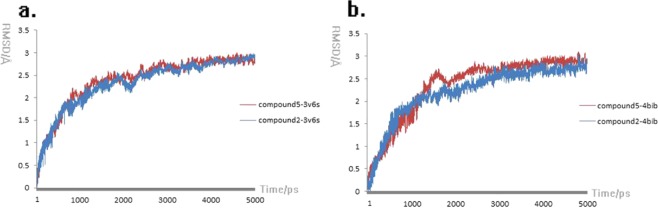
Molecular dynamics results of ligand-3v6s complexes (**a**) and ligand-4bib complexes (**b**) in 310 K.

The results of molecular dynamics were shown in Fig. 6a,b, the tendency of red (compound5-3V6S) and blue curve (compound2-3V6S) showed similar increasing tendency in early 3 ns, the RMSD values of red curve were maintained about 2.793 Å in the last 2 ns, and the RMSD values of blue curve showed a similar tendency with RMSD values of 2.755 Å in the last 2 ns. In Fig. 6b, in early 3 ns simulations, the red RMSD values curve generated fluctuant data compare to blue curve in 1.7–2.9 ns, but in last 2 ns simulations the two curves regenerated the similar tendency with the mean RMSD values of 2.843 Å and 2.718 Å respectively.

In addition, the change of interaction between the two compounds and key amino acids of the active pocket in different protein was evaluated for explaining the results of molecular dynamics (Fig. S1). With the substitution of the ligand, the conformational change of unstable target-protein complexes may be occurred in the binding site, ligand-receptor conformation which was beneficial for promoting or inhibiting effect will be changed, and in such situations the binding capacity and affinity were decreased, then the RMSD values would fluctuate dramatically. The basic, acidic and interactional amino acids in active pocket of 3V6S and 4BIB were recorded in Table 4, of the protein 3V6S, naphthalene and glucoside moiety of compound 2 formed many hydrogen bond and arene interactions to the residues Asn152, MET149, Gly76 before MD simulation, the similar residues interaction can also be seen in the compound 5-3V6S complex in Fig. 5b, these key interactions in compound 5 were maintained after the MD simulation, and the residue Glu111 in pocket and formed a new hydrogen bond to phenolic group in compound. Of the protein 4bib, the Asp822 and Val757 were the key residue in pocket according to the inhibitor-4bib complex in Fig. 5c. Compound 5 formed many more potent hydrogen bond and arene interactions to Asp822, Val757, Leu686 and Val694 after the molecular simulation than before. the results of compound 2 were similar to those of compound 5, where the glucoside moiety formed new hydrogen bond interactions with the simulated 4bib. The amino acids of the same compound at different stages did not demonstrated obvious distinction, This result was consistent with the RMSD values curve, which revealed that the two compounds possesses potent binding capacity and affinity to the protein ASK1 and JNKs.

**Table 4:** Key amino acids in active pocket around ligand after docking, and two temperature molecular dynamics.

Ligand	3v6s	4bib
Compound 2 (docking)	Met149 Gly76 Lys93 Glu111 Leu206 Asn152 Asp150 Asp207	Leu686 Val757 Glu755 Lys688 Asp822 Asp807 Glu725 Lys709
Compound 2 (MD)	Met149 Met146 Glu111 Gln75 Leu206 Glu147 Asp207 Lys93	Asp822 Lys688 Asp807 Lys709 Glu725 Glu755 Arg767
Compound 5 (docking)	Met146 Met149 Leu206 Glu111 Lys93 Asp207	Leu686 Val757 Lys688 Glu755 Asp822 Lys709 Asp807 Glu725
Compound 5 (MD)	Met149 Asp150 Lys93 Glu111 Asp207	Val757 Leu686 Val694 Asp822 Asp807 Lys688 Lys805 Asp803

## Conclusion

The network pharmacology identified the key pathway of the anti-inflammatory effect of the constituents in the flower of *Trollius chinensis Bunge*. Four putative pathways related to inflammation were generated from the gene and KEGG pathways, then the molecular dynamics and docking technology of the key kinases in pathways demonstrated that the CID21578038 and CID20055288 were stable in the protein ASK1 and JNKs respectively. The current investigation revealed that two effective compounds in the flower of *Trollius chinensis Bunge* played a crucial role in the process of inflammation by targeting ASK1 and JNKs. This comprehensive method is only applicable for study to predict pathway or target of multi-component Chinese herbal, but also in a multi-component herbal prescription. The further experimental data from the *in vitro* and *in vivo* experiments will be utilized to validate and optimize this method constantly.

## Supplementary information

Dataset 1

gene network

pharmmapper result (geneid)
