# Supplementary material for: Network pharmacology-based identifcation of potential targets of the flower of Trollius chinensis Bunge acting on anti-inflammatory effectss
Source: Sci Rep. 2019 May 30;9:8109. doi: 10.1038/s41598-019-44538-z (PMC6542797; doi:10.1038/s41598-019-44538-z)
Supplement: Supplementary file 1 — Dataset 1 [file 41598_2019_44538_MOESM1_ESM.pdf]

Network pharmacology-based identifcation of potential targets of the flower of *Trollius chinensis Bunge* acting on anti-inflammatory effect

Jing-wei Liang[1,†], Ming-yang Wang[1,†], Kamara Mohamed Olounfeh[1], Nan Zhao[1], Shan Wang[1], Fan-hao Meng[1,*].

1.School of Pharmacy, China Medical University, Liaoning 110122, China

*****Correspondence: e-mail@fhmeng@cmu.edu.cn
† These authors have contributed equally to this work

Table S1 The overlapping result of 20 compounds and their corresponding UNIPROTKB

Compound 1	Compound 1	Compound 2	Compound 2	Compound 3	Compound 3	Compound 4	Compound 4	Compound 5	Compound 5	Compound 6	Compound 6	Compound 7	Compound 7	Compound 8	Compound 8
PDBID	UNIPROTKB	PDBID	UNIPROTKB	PDBID	UNIPROTKB	PDBID	UNIPROTKB	PDBID	UNIPROTKB	PDBID	UNIPROTKB	PDBID	UNIPROTKB	PDBID	UNIPROTKB
1A1N	P30685	1A1M	P30491	5TEZ	P03485	1A1N	P30685	6D5M	P01112	1A13	P01515	3LKQ	P30685	1A7C	P05121
1A9B	P30685	1A1O	P30491	1AKJ	P01732	1A30	P04585	6D5V	P01112	2CZP	P01515	3LKR	P30685	9PAI	P05121
1A9E	P30685	1A4Y	P13489	1B0G	Q9NPA0	1A7X	P62942	6Q21	P01112	5XOT	P30685	3LKS	P30685	2D3I	P02789
1A8J	P01709	1Z7X	P13489	1B6E	Q13241	4ODR	P62942	721P	P01112	6BJ2	P30685	3MV7	P30685	1AN8	Q8NKX2
1DCL	P01709	2BEX	P13489	1BZ9	P01899	5I7P	P62942	821P	P01112	6BJ3	P30685	2RUK	P04637	1HQR	Q8NKX2
1AQK	P0DOY3	2Q4G	P13489	1CB0	Q13126	5I7Q	P62942	1A07	P12931	5F6X	P63279	2VUK	P04637	1KTK	Q8NKX2
7FAB	P0DOY3	5M9Q	P03950	1CG9	P03204	1A9M	P03366	4MXO	P12931	5F6Y	P63279	2WGX	P04637	1AZ7	Q99062
1AR2	P01593	5M9R	P03950	1CWR	P30305	1AJV	P03366	4MXX	P12931	5FQ2	P63279	2X0U	P04637	2D9Q	Q99062
1BWW	P01593	5M9S	P03950	1D9K	P04213	1AJX	P03366	4MXY	P12931	6N32	P01834	2X0V	P04637	1B3J	Q29983
1REI	P01593	5M9T	P03950	1EKU	P01579	1AXA	P03366	4MXZ	P12931	6N35	P01834	2X0W	P04637	1HYR	Q29983
1WTL	P01593	5M9V	P03950	1G3J	P35222	1MER	P03366	1A81	P43405	1A5R	P63165	2XWR	P04637	4UAO	P01835
1B0W	P01594	1A6Z	Q30201	1IVY	P10619	4YC6	P61024	1B47	P22681	6E0P	P63165	2YBG	P04637	1BHF	P06239
4K07	P01594	1C42	Q30201	1KPR	P13747	1CMK	P61925	1BUH	P61024	6E0T	P63165	2YDR	P04637	5MTN	P06239
1B3T	P03211	1DE4	Q30201	1NFD	P01849	2JDT	P61925	1YEG	P01863	1A0C	P02681	2Z5S	P04637	3L5J	P40189
1VHI	P03211	1A7S	P20160	1M6O	Q95HB9	1S6P	P03366	2JDV	P61925	1AU1	P01574	2Z5T	P04637	1BP1	P17213
5WMF	P03211	1AE5	P20160	3L3K	Q95HB9	1S6Q	P03366	1CQ0	O43612	1AXM	P05230	3D05	P04637	1EWF	P17213
5WUM	P03211	1FY1	P20160	1MI5	Q3KST2	1S9E	P03366	1E4U	O95628	3BAH	P05230	3D06	P04637	1ZGL	P02686
5WUN	P03211	1FY3	P20160	3SKO	Q3KST2	1S9G	P03366	1EXT	P19438	3BAO	P05230	3D07	P04637	5QCH	P25774
1BBN	P05112	1AKL	Q03023	1MT7	Q9YYH6	1SBG	P03366	1F9P	P02775	1B3T	P03211	3D08	P04637	5QCI	P25774
5FHX	P05112	1JIW	Q03023	3GIV	Q9YYH6	6DUG	P03366	1FPR	P29350	1BF5	P42224	3D09	P04637	5QCJ	P25774
1BBR	P02671	1KAP	Q03023	1N59	Q9QDK7	6DUH	P03366	1FQV	Q13309	1D7N	P01514	3D0A	P04637	1CD9	P40223
1FZF	P02671	3VI1	Q03023	3TID	Q9QDK7	6ELI	P03366	1P22	P63208	1DG3	P32455	3DAB	P04637	2D9Q	P09919
1FZG	P02671	1A07	P14079	1QD0	A2KD59	1AGB	P30460	1FYV	Q15399	1DQM	P01863	3DAC	P04637	5GW9	P09919
1LT9	P02671	1BD2	P14079	2X89	A2KD59	1AGC	P30460	1FYW	O60603	1Y6P	P00592	3IGK	P04637	1UVQ	O43612
1LTJ	P02671	1HHK	P14079	1S9W	P78358	1AGD	P30460	1GUV	Q13231	1DWY	P10279	3IGL	P04637	1WSO	O43612
1N86	P02671	1IM3	P14079	2OG3	P59595	1E7B	P02768	1H27	P46527	1E1G	P04156	3KMD	P04637	1D0A	P43489
5CFA	P02671	2AV1	P14079	1T1W	O11822	1E7C	P02768	1HDJ	P25685	1EJ6	P11079	3KZ8	P04637	2HEV	P43489
1BHT	P14210	2AV7	P14079	1TXP	P07910	1E7E	P02768	1IO1	P06179	1F45	P29459	3LW1	P04637	2HEY	P43489
5CT3	P14210	5IRO	P14079	1UXS	P13285	1E7F	P02768	1J7V	Q13651	1FBV	P43403	3OQ5	P04637	1D9C	P07353
6FUT	P00746	1AV1	P02647	3B08	P43355	1E7G	P02768	1JX3	P05107	1FN9	P03527	3PDH	P04637	1D9G	P07353
1C3D	P01024	6CM1	P02647	1XR8	P30464	2BX8	P02768	1LDJ	Q13616	1ITF	P01563	3Q01	P04637	1RFB	P07353
1GHQ	P01024	2GMC	P02788	1YDP	P16104	2BXA	P02768	1N3Y	P20702	1J4X	P51452	4X34	P04637	1D9K	P01910
6EHG	P01024	2GMD	P02788	3KYN	P17693	5UJB	P02768	1OXN	Q96CA5	1JFW	P04610	4XR8	P04637	1IAK	P01910
1CD0	P01721	2HD4	P02788	5EE7	P47871	5VNW	P02768	3J0A	O60602	1JMU	P07939	4ZZJ	P04637	1JL4	P01910
1PEW	P01721	2PMS	P02788	5XEZ	P47871	4JSX	P42345	1QYM	O75832	1JSP	Q92793	5A7B	P04637	1D9K	P06343
2CD0	P01721	1B1X	O77811	2BSS	Q98Y46	4JT5	P42345	5VHQ	O75832	6FR0	Q92793	5AB9	P04637	1IAK	P06343
5IR3	P01721	1B7U	O77811	2BVO	Q70A61	4JT6	P42345	5VHR	O75832	6FRF	Q92793	5ABA	P04637	1JL4	P06343
1CE1	Q6PYX1	1B7Z	O77811	2BVQ	Q70A61	5FLC	P42345	1T5W	P27705	1K3B	P53634	5AOI	P04637	1DLH	P04664
4NHH	Q6PYX1	1F9B	O77811	2BVP	Q8URG0	5GPG	P42345	1T5X	P27705	2DJF	P53634	5AOJ	P04637	2G9H	P04664
4QGT	Q6PYX1	1I6B	O77811	2CIK	O19626	5H64	P42345	1U58	A2Q6L5	4OEL	P53634	5AOK	P04637	1DR9	P33681
5VGP	Q6PYX1	1QJM	O77811	2FYY	O19626	5WMF	P03211	4A0C	Q86VP6	4OEM	P53634	5AOL	P04637	1I8L	P33681
1CFA	P01031	3CR9	O77811	2FZ3	O19626	5WUM	P03211	1UCP	Q9ULZ3	1K5K	P04613	5AOM	P04637	1DYQ	P0A0L2
6H03	P01031	5USQ	P36897	2H6P	P33260	5WUN	P03211	2KN6	Q9ULZ3	1KPI	P9WPB5	5BUA	P04637	1ESF	P0A0L2
6H04	P01031	6B8Y	P36897	2CLR	P27797	1BD2	P0C213	3J63	Q9ULZ3	3HEM	P9WPB5	5ECG	P04637	1E4Q	O15263
1DEQ	P02676	1BIY	O77698	3D0W	P27797	4E5X	P0C213	5H8O	Q9ULZ3	1L1E	P9WPB3	1A6A	P01912	1EXT	P19438
1JY2	P02676	1CE2	O77698	2D9I	Q86UW6	4FTV	P0C213	6N1H	Q9ULZ3	1LDJ	P62877	1AIN	P04083	1F9P	P02775
1JY3	P02676	1BLF	P24627	2GW5	Q8N423	1BPV	Q8WZ42	1UJR	Q9CZW6	1LDK	P62877	1B09	P04083	1FV1	Q30154
1DGX	P01597	5CRY	P24627	2HJK	Q70AA1	2ILL	Q8WZ42	2RSF	Q9CZW6	1U6G	P62877	1QLS	P04083	1G2S	Q9Y258
1F6L	P01597	5HBC	P24627	2HJL	Q1KW74	2J8H	Q8WZ42	4QPL	Q9CZW6	2HYE	P62877	5VFW	P04083	4N5E	P01738
3UPA	P01597	1C16	Q31615	2KNU	Q03463	2J8O	Q8WZ42	1UMH	Q8OUW2	2LGV	P62877	1AU8	P08311	1G91	P55773

4OB5	P01619	1C5A	P01032	3MRJ	Q03463	2NZI	Q8WZ42	1UMI	Q80UW2	1LUZ	P18378	1CGH	P08311	5NRA	Q13231
4ODX	P01619	1CA7	P14174	3MRL	Q03463	5JOE	Q8WZ42	2E33	Q80UW2	1NI2	P15311	1KYN	P08311	1H15	P03198
5LVE	P06312	2YPL	Q70XD7	2PA2	P27635	6IOY	Q8WZ42	2RJ2	Q80UW2	1PFA	P07288	1T32	P08311	1H6H	Q15080
1DLE	P00751	3BH9	Q8WVV4	5AJ0	P27635	1BQH	P01901	5B4N	Q80UW2	1QNZ	P01665	1B0F	P08246	1H8U	P13727
1Q0P	P00751	5VGE	P10321	2RIG	P30123	1FOO	P01901	1VLK	P03180	1RRP	P49792	1H1B	P08246	1HF1	P12544
3HS0	P00751	3D25	Q92619	2RMX	P26715	1KPV	P01901	1X4J	Q9H0F5	1T2Z	Q9Y6Y9	1B3T	P03211	1HYR	P26718
1DUG	P02679	3FT3	Q92619	2YU7	P26715	1LEG	P01901	4V3K	Q9H0F5	3ULA	Q9Y6Y9	1VHI	P03211	1I1R	Q98823
3FIB	P02679	3D2U	P08560	2X40	P04583			4V3L	Q9H0F5	2D07	Q13569	1BGC	P35833	1I3R	P04230
4B60	P02679	3DBX	Q5GL29	2X4R	P06725			1YCK	O75594	1Y8Q	Q9UBE0	1BGD	P35834	1I85	P42081
1EAW	Q9Y5Y6	3FKU	Q2F4V2	2X4S	Q33CG5			2AK5	Q13191	1YUR	Q99584	1BGE	P35834	1IEA	P01904
1EAX	Q9Y5Y6	3MGO	Q2F4V2	2XPG	P60201			2BZ8	Q13191	2AI6	Q9NRX4	1C4Z	Q05086	1ILK	P22301
2FMV	Q9Y5Y6	3FQW	Q9Y4H2	3RL1	P04439			2D06	Q13191	4F09	O75928	1D5F	Q05086	1ILM	P01589
2GV6	Q9Y5Y6	3FQX	Q9Y4H2	3RL2	P04439			5FER	P51668	2B5L	P11207	1EQX	Q05086	1ILP	P25024
2GV7	Q9Y5Y6	3H7B	P38110	2YPK	Q70XD7			5TUT	P51668	2B5M	Q16531	2KR1	Q05086	1J7V	Q13651
3BN9	Q9Y5Y6	3H9H	P38110	1MES	P03366			2CT7	Q96EP0	2K45	P21579	4GIZ	Q05086	1JLI	P08700
5LYO	Q9Y5Y6	3H9S	P38110	6GIN	Q04771			5X0W	Q96EP0	2LHA	P21579	1CDQ	P13987	1KCG	Q9BZM4
1EZV	P01647	3H9R	Q04771	6GIP	Q04771			2DAY	Q96BH1	5Y18	Q9UER7	1CDR	P13987	1KG0	P03205
2V7H	P01647	3MTF	Q04771	3HG1	Q8WLS4			2DMF	Q96BH1	5Y60	Q9UER7	1CDS	P13987	1KTD	P00021
2VWE	P01647	3OOM	Q04771	3O4L	Q8WLS4			3DKB	P21580	2O6S	Q4G1L3	1CHO	P68390	1LB5	Q9Y6Q6
3U0W	P01647	3Q4U	Q04771	3I6G	P59596			3OJ3	P21580	3A79	Q4G1L3	1EIG	O00175	1LK3	P20759
4PD4	P01647	4BGG	Q04771	3I6K	Q692E0			5UZK	P17612	2P4D	P33064	1ES0	P04228	1LQS	P17150
1FHC	P08603	6D03	P02786	3T02	Q692E0			6C0U	P17612	2PZI	P9WI73	2OKK	Q05329	3B2U	P01857
1HAQ	P08603	6D04	P02786	3LV3	Q01668			2J67	Q9BXR5	4Y0X	P9WI73	1ESR	P80075	2KS1	P04626
4AYI	P08603	6D05	P02786	3MDY	O00238			2KHW	P0CG47	4Y12	P9WI73	1EVS	P13725	1MGS	P09341
4AYM	P08603	1M27	O60880	3MGT	Q1WDM0			6MSB	P0CG47	2Q05	P07239	1FX9	P00592	1N26	P08887
6BIJ	P02675	1I3Z	Q13291	3MRF	Q3KSU1			6MSD	P0CG47	2RF6	P07239	1LV9	P02778	1OQD	Q02223
6BIL	P02675	2DZF	Q13291	3MRK	P02771			6MSG	P0CG47	3CM3	P07239	1M4R	Q9GZX6	1PLF	P02777
1G86	Q05315	2IE9	Q13291	3MRM	Q9DIT6			6N13	P0CG47	2WZL	P0C569	1MFG	P04626	1PYW	P0A0L3
1HDK	Q05315	2IFL	Q13291	3MRN	Q9DIT6			2LAJ	Q96PU5	2Z62	O00206	1MFL	P04626	1QNK	P19875
6A1U	Q05315	2IG5	Q13291	3MRR	Q92959			2LB2	Q96PU5	2Z63	O00206	1MW4	P04626	1R5I	P11133
6A1V	Q05315	1DDH	P04582	3OXS	P0C6H2			3JW0	Q96PU5	2Z66	O00206	1N8Z	P04626	1RI9	O15117
6A1X	Q05315	1HHG	P04582	3S6C	P15812			5HPK	Q96PU5	6B3T	P9WFK7	1OVC	P04626	1RJ7	Q92838
6A1Y	Q05315	1Q03	P04582	3TIE	Q49PI7			3CON	P01111	3RJR	P07200	1QR1	P04626	1RJT	O14625
1HEZ	P01871	1SZT	P04582	3V5D	E0YFW1			5UHV	P01111	5VQF	P07200	1S78	P04626	1SJE	P12495
2AGJ	P01871	1DTZ	Q9TUM0	6EK0	Q96L21			2YHN	Q8WY64	3W3G	Q9NR97	2A91	P04626	1T5W	P27705
1YMH	Q51918	1I6Q	Q9TUM0	5A2Q	P62701			3A7C	Q9QUN7	3W3J	Q9NR97	2JWA	P04626	1U58	A2Q6L5
6BAH	Q51918	2J4U	Q9TUM0	5A8L	P18621			5D3I	Q9QUN7	3W3K	Q9NR97	5P2P	P00592	1UVQ	E9PMV2
1HOU	P01764	1DYN	Q05193	4V6W	P05388			3NG2	O88846	3WPB	Q2EEY0	1HM6	P19619	1VLK	P03180
1OHQ	P01764	6DLV	Q05193	1KPV	P04857			5AIT	O88846	3WPE	Q5I2M5	1MCX	P19619	1YCK	O75594
5JPN	P0C0L4	1E27	P24740	2VAB	P04857			5AIU	O88846	3WPF	Q9EQU3	1IRU	P33672	1ZXT	Q76RJ0
5JTW	P0C0L4	1NCP	P24740	1GHQ	P20023			3WI4	P30690	4HOU	P09914	1KJ6	P81534	2CH9	O76096
1J74	Q15819	3LZS	P24740	2MCZ	P17927			4DHI	Q9XVR6	4JRX	C5MK56	1N3U	P09601	2E7L	A2NTY6
1J7D	Q15819	3LZU	P24740	5NRF	Q13231			4I6E	Q9R194	4OR9	P16298	1N6U	P48551	2FFK	O10647
1ZGU	Q15819	1EFX	P04222	1HHH	P12901			4R30	O14818	4QRP	X2G898	1PF6	P50750	2G9H	Q52T95
5AIT	Q15819	2DL2	P43627	1HHJ	P04588			5L62	P20618	4QRS	Q9YRL3	1S3R	Q9LCB8	2H32	P12018
2C2V	P61088	2DLI	P43627	3IXO	P04588			5DSV	P25788	4QRT	Q9YRL8	1VZN	P03126	2H32	P15814
5VOO	P61088	1QGK	P52292	1I4F	P43358			4WZ0	Q5X159	4ZAI	O43927	1YSK	P04054	2HCC	Q16663
5YWR	P61088	1QGR	P52292	1IGA	P01876			5D0I	Q6ZSG1	5C2B	P01704	1ZIW	O15455	2HDL	O95715
1JVK	P0DOY2	5H43	P52292	1IM3	P09713			5EU6	A0A592	5DXU	O00329	2F8A	P07203	2HEV	P23510
1LIL	P0DOY2	1EXT	P19438	1IM9	P43626							2PK2	O60563	2PA2	P27635
6BG7	P14174	1FT4	P19438	1QQD	P30504							2YEZ	Q5ZMD1	4UG0	Q96L21
6FVH	P14174	1ICH	P19438	1IW2	P07360							3BEV	Q95601	4UG0	P62701
1CE6	P04858	1NCF	P19438	1J7V	Q13651							3HHC	Q8IZI9	4UG0	P18077
1CQ0	O43612	1TNR	P19438	1J8I	P47992							3JVG	A5HUM9	4UG0	P18124

1R02	O43612	1F16	Q07812	1JIW	Q03026
1UVQ	O43612	1F95	O43521	1JW1	Q29477
1WSO	O43612	5VX2	O43521	1M05	P12977
1CX8	P02786	5VX3	O43521	1M7M	P00813
1OTS	P01808	5WOS	O43521	1OZZ	P84156
3EJZ	P01808	1F9P	P02775	1QVO	P04602
4KKB	P01808	1NAP	P02775	1R3H	Q31206
4MQX	P01808	1TVX	P02775	1S8D	O11818
1Q3X	O00187	1FG9	P15260	1SYS	Q9Y5X3
1SZB	O00187	1FYH	P15260	1SYV	Q9TQB0
1ZJK	O00187	1JRH	P15260	1T5W	P27705
3TVJ	O00187	1FZK	P04857	1U58	A2Q6L5
1VVC	P68638	2GSX	P20023	1UVQ	E9PMV2
1WOR	P27918	3OED	P20023	1VLK	P03180
1YLA	P61086	1GKG	P17927	1WOV	Q07352
1ZEC	P03404	5FO9	P17927	1XEQ	P03502
2FB4	P0CG04	1GUV	Q13231	3BZH	P51965
2A4D	Q13404	5NRF	Q13231	1YCK	O75594
2B39	Q2UVX4	1HHH	P12901	1YPZ	P06334
2C2L	Q9WUD1	1HHJ	P04588	1Z2M	P05161
2CM4	Q5YD59	3IXO	P04588	1ZS8	Q860W6
2CSW	O76064	1I4F	P43358	2CDX	Q8T0W8
2PA2	P27635	2WAO	P43358	2GFU	P52701
2QZW	P0CY27	1IGA	P01876	2LR1	P16778
2XJY	P25791	1OW0	P01876	2NN6	Q06265
3VN9	P52564	2QEJ	P01876	3THW	P43246
3A79	Q9EPW9	3CHN	P01876	2QQH	P07357
3B21	Q8VSD5	1IM9	P43626	2XRC	P05156
4XZX	Q8VSD5	1NKR	P43626	3B2D	O95711
3FRP	Q91132	1QQD	P30504	3LJB	P20591
3OJY	P07358	1IW2	P07360	3PT2	Q6TQF5
3SDY	Q91MA7	1J7V	Q13651	4PJ1	P10809
5UMN	Q91MA7	1J8I	P47992	4PVF	P34897
6BKM	Q91MA7	2RN4	Q03026	5FMW	P02748
6CEX	Q91MA7	1JW1	Q29477	3PRX	D3JIB2
3T5O	P13671	1M05	P12977	3SDY	Q91MA7
4AIZ	Q5NV90	1M7M	P00813	3T5O	P13671
4GMS	P03435	1OZZ	P84156	4AIZ	Q5NV90
4PRH	C5MK56	1QVO	P04602	4D3C	U3KM01
4OGX	S6C4R2	1R3H	Q31206	4GMS	P03435
4OGY	S6C4R2	1S8D	O11818	4JRX	C5MK56
4PUB	S6C4R2	1SYS	Q9Y5X3	4LLU	S6C4R2
5W1K	S6C4R2	1SYV	Q9TQB0	4LLW	S6B291
4OQT	S6B291	1T5W	P27705	4NM4	S6C4S0
4YDV	S6B291	1U58	A2Q6L5	4O5I	R9U684
5VQM	S6B291	1VLK	P03180	4OM7	Q9Y2C9
4NM4	S6C4S0	1WOV	Q07352	4OPA	Q20NS3
4WE8	R9U684	1XEQ	P03502	4PRN	Q1HVF7
4OM7	Q9Y2C9	1XR9	P51965	4QRP	X2G898
4OPA	Q20NS3	1YCK	O75594	4QRS	Q9YRL3
4QRP	X2G898	1YPZ	P06334	4QRT	Q9YRL8
4QRS	Q9YRL3	1Z2M	P05161	4RX4	Q8JDI3
4QRT	Q9YRL8	1ZS8	Q860W6	5FLX	P22090
4RX4	Q8JDI3	2CDX	Q8T0W8	5FOB	Q89859

3KUZ	O60486	4UGO	P18621
3L9R	Q1L1H6	4UGO	P26373
5V61	P04608	4V6W	P05388
3OG4	Q8IU54	4V6X	Q8NC51
5IXD	Q8IU57	4V6X	P30050
3PWV	Q9YKD7		
3S98	P17181		
3UX9	P01562		
4CVX	Q5ZJG4		
4D0D	O46789		
4CW1	A0ZXM3		
4E0R	O46790		
4F7C	A1L565		
4JRX	C5MK56		
4QRP	X2G898		
4QRS	Q9YRL3		
4QRT	Q9YRL8		
5B0V	P35260		

5T2C	Q96L21	2GFU	P52701	5IQ7	V9QIE5
5A2Q	P62701	2NN6	Q06265	5IQ9	Q1HSF8
5A8L	P18621	3THW	P43246	7FAB	P01825
4V6W	P05388	2QQH	P07357		
5FLX	P22090	2XRC	P05156		
5U3J	V9QIE5	3B2D	O95711		
		3LJB	P20591		
		3PT2	Q6TQF5		
		4PJ1	P10809		
		4PVF	P34897		
		5X3V	P34897		
		6DK3	P34897		
		5FMW	P02748		
		6DLW	P02748		

Compound 9	Compound 9	Compound 10	Compound 10	Compound 11	Compound 11	Compound 12	Compound 12	Compound 13	Compound 13	Compound 14	Compound 14	Compound 15	Compound 15	Compound 16	Compound 16
PDBID	UNIPROTKB	PDBID	UNIPROTKB	PDBID	UNIPROTKB	PDBID	UNIPROTKB	PDBID	UNIPROTKB	PDBID	UNIPROTKB	PDBID	UNIPROTKB	PDBID	UNIPROTKB
3FB6	P01837	1WH9	P23396	1A13	P01515	1A06	Q63450	1A1N	P30685	5XOT	P30685	1A7L	P0AEX9	1A7F	P01308
3HFM	P01837	4UG0	P23396	2CZP	P01515	1A29	P62157	6BJ8	P30685	6BJ2	P30685	1A9U	Q16539	2VXW	P13501
4KK5	P01837	4V6X	P23396	6HM6	P43405	1A7A	P23526	1AII	P12429	6BJ3	P30685	4R8I	P13500	5CMD	P13501
4KK8	P01837	5A2Q	P23396	6HM7	P43405	1AJI	P0DP23	1AXN	P12429	6BJ8	P30685	1F66	P0C0S5	5COY	P13501
4KVC	P01837	5AJ0	P23396	1AII	P12429	1AK4	P62937	1B3T	P03211	1AII	P12429	1GZ2	Q9PRS8	3H8B	P07711
4QNP	P01837	5FLX	P23396	1AXN	P12429	1AUI	P63098	1VHI	P03211	1AXN	P12429	1K12	Q7SIC1	3H8C	P07711
4ZXB	P01837	5LKS	P23396	1AIN	P04083	2JOG	Q08209	5WUM	P03211	1B3T	P03211	1NR2	Q92583	3HHA	P07711
6FAB	P01837	5OA3	P23396	1BO9	P04083	1B9C	P42212	5WUN	P03211	1VHI	P03211	1U2P	P9WIA1	6EZP	P07711
1A1W	Q13158	5T2C	P23396	1QLS	P04083	5NDJ	P80372	1BR9	P16035	1YY6	P03211	1AOI	P02281	6EZX	P07711
1A1Z	Q13158	5VYC	P23396	5VFW	P04083	4ZF5	P42212	1GXD	P16035	4PRA	P03211	3C1B	P06897	6F06	P07711
1E3Y	Q13158	6EK0	P23396	1AJ7	P01857	5F9G	P42212	2TMP	P16035	2TMP	P16035	2HUE	P62799	1DOK	P13500
1E41	Q13158	6FEC	P23396	1AQK	P01857	5FGU	P42212	4ILW	P16035	4ILW	P16035	1ATK	P43235	5GOI	P62987
2GF5	Q13158	6G51	P23396	6DE7	P01857	5MA5	P42212	1CK7	P08253	1CK7	P08253	1AX8	P41159	5GOJ	P62987
3EZQ	Q13158	6G53	P23396	1ALU	P05231	5MA6	P42212	1CXW	P08253	1CXW	P08253	1BV8	P01023	5GOK	P62987
3OQ9	Q13158	6G5H	P23396	1IL6	P05231	5MA8	P42212	3AYU	P08253	3AYU	P08253	1C3T	P0CG48	5HPK	P62987
1AH1	P16410	6G5I	P23396	1N2Q	P05231	1BZI	P25445	1CQ0	O43612	1CQ0	O43612	1C9Q	P98170	5HPL	P62987
1H6E	P16410	2CQL	P32969	1P9M	P05231	1DDF	P25445	1R02	O43612	1R02	O43612	1CA4	Q12933	5HPS	P62987
1I85	P16410	2KHW	P62979	2IL6	P05231	2NA7	P25445	1UVQ	O43612	1UVQ	O43612	1CDF	P25942	5HPT	P62987
1I8L	P16410	2KOX	P62979	1QYF	P42212	2O5G	P11799	1WSO	O43612	1WSO	O43612	1CFP	P02638	5J26	P62987
2X44	P16410	2KTF	P62979	1QYO	P42212	4OY4	P11799	1EXT	P19438	1EXT	P19438	3IQO	P02638	1ZOJ	P9WIA1
3BX7	P16410	2KWU	P62979	1P63	P05230	5U6Y	P11275	1FT4	P19438	1FT4	P19438	3IQQ	P02638	2LUO	P9WIA1
3OSK	P16410	2KWV	P62979	4XKI	P05230	1CFF	P23634	1ICH	P19438	1ICH	P19438	3LKO	P02638	1U8F	P04406
5GGV	P16410	2LOF	P62979	4YOL	P05230	1CQ0	O43612	1NCF	P19438	1NCF	P19438	3LK1	P02638	1ZNQ	P04406
5TRU	P16410	2LOT	P62979	1B50	P10147	1R02	O43612	1TNR	P19438	1TNR	P19438	3LLE	P02638	2FEH	P04406
5XJ3	P16410	2XK5	P62979	1B53	P10147	1UVQ	O43612	1F9P	P02775	1F9P	P02775	5DKQ	P02638	3GPD	P04406
1AIK	P04578	3AXC	P62979	1RCB	P05112	1WSO	O43612	1NAP	P02775	5LMV	P80372	5DKR	P02638	4WNC	P04406
6DE7	P04578	3I3T	P62979	2B8U	P05112	1EXT	P19438	1TVX	P02775	3GC7	Q16539	5ER4	P02638	4WNI	P04406
6DLN	P04578	3K9P	P62979	2B8X	P05112	1FT4	P19438	5IMQ	P80372	3GCP	Q16539	5ER5	P02638	6ADE	P04406
1AJ7	P01857	3N30	P62979	2B8Y	P05112	1ICH	P19438	6GZZ	P80372	3HV3	Q16539	1CS9	P68431	1UR6	P62837
1AQK	P01857	3N32	P62979	1PNJ	P23727	1NCF	P19438	1FKA	P80373	6BUW	P80372	1CT6	P68431	1W4U	P62837
1AXS	P01857	3NHE	P62979	1QAD	P23727	1TNR	P19438	1JGO	P80373	6BZ6	P80372	1O9S	P68431	2CLW	P62837
5U66	P01857	3NOB	P62979	2PNA	P23727	1F9P	P02775	1JGP	P80373	6BZ7	P80372	6HTS	P68431	2ESK	P62837
5V43	P01857	3NS8	P62979	2PNB	P23727	1NAP	P02775	1X18	P80376	6BZ8	P80372	1DS6	P52566	5ULH	P62837
5V4E	P01857	3PHD	P62979	6D86	P23727	1TVX	P02775	1MJ1	P80377	6CAE	P80372	5H1D	P52566	5ULK	P62837
6DCV	P01857	3PHW	P62979	6D87	P23727	5NRA	Q13231	6C5L	P80380	6CAO	P80372	2W2T	P15153	5VZW	P62837
6DCW	P01857	3TBL	P62979	6G6W	P23727	5NRF	Q13231	1MVR	Q5SHN3	1TWT	P80373	2W2V	P15153	6HPR	P62837
6N35	P01857	3VDZ	P62979	1D7N	P01514	6B94	P09382	3A1P	Q5SHP2	4V4I	P80373	2W2X	P15153	1WA8	P9WNK5
1ALY	P29965	4R62	P62979	1DS6	P52566	1IG1	P53355	4V4G	Q5SHQ5	6CFJ	P80374	1DYT	P12724	3FAV	P9WNK5

1I9R	P29965	5WVO	P62979	2W2T	P15153	1JKK	P53355	1G1X	Q5SJ76	6CFK	P80374	1H1H	P12724	1WH9	P23396
3LKJ	P29965	5YDK	P62979	1EKU	P01579	1JKL	P53355	2KJV	Q5SLP8	6CFL	P80374	1QMT	P12724	4UGO	P23396
3QD6	P29965	6DC6	P62979	1F42	P29460	1JKS	P53355	2KJW	Q5SLP8	6N9E	P80374	4OXF	P12724	4V6X	P23396
6BRB	P29965	6G18	P62979	1F9P	P02775	6GY5	P53355	3ZZP	Q5SLP8	6N9F	P80374	4X08	P12724	6G53	P23396
1AUT	P04070	2LJ5	P62987	1FBV	P43403	1ILK	P22301	1FQ3	P10144	1X18	P80376	3GV6	P84233	6G5H	P23396
1LQV	P04070	2MBH	P62987	1JRH	P15260	1INR	P22301	1IAU	P10144	1MJ1	P80377	5XBK	P84233	6G5I	P23396
1PCU	P04070	2MJB	P62987	1GA3	P35225	1J7V	P22301	1GD8	Q9Z9H5	6C5L	P80380	1FEW	Q9NR28	2CV5	O60814
2PCT	P04070	2MUR	P62987	1HM6	P19619	1LK3	P22301	1GUV	Q13231	1MVR	Q5SHN3	1OXQ	Q9NR28	2D58	P55008
3F6U	P04070	2N3U	P62987	1IRS	P24394	1Y6K	P22301	5NR8	Q13231	1PN7	Q5SHN3	1TW6	Q9NR28	2G2B	P55008
3JTC	P04070	2N3V	P62987	1ILM	P31785	2H24	P22301	5NRA	Q13231	1PN8	Q5SHN3	1XB0	Q9NR28	2HKY	Q9H1E1
4DT7	P04070	2N3W	P62987	1Z92	P01589	2ILK	P22301	5NRF	Q13231	1QZC	Q5SHN3	4Q1R	P09382	2HM2	Q8WXC3
1BX2	P01911	2NBD	P62987	6E8K	P14784	1LQS	Q13651	1J7V	Q13651	3A1P	Q5SHP2	4Q26	P09382	4QOB	Q8WXC3
1YMM	P01911	2NBE	P62987	1IRL	P60568	1Y6M	Q13651	1LQS	Q13651	4V4G	Q5SHQ5	4Q27	P09382	2KHW	P62979
2WBJ	P01911	2RSU	P62987	1ITA	P01583	1Y6N	Q13651	1Y6K	Q13651	1G1X	Q5SJ76	4Q2F	P09382	2KOX	P62979
6CPO	P01911	4HJK	P62987	1ITF	P01563	5IXI	Q13651	1Y6M	Q13651	2KJV	Q5SLP8	4XBL	P09382	2KTE	P62979
3ALQ	P20333	4PIG	P62987	1N6U	P48551	6EQ9	P53779	1JBI	O43405	1FQ3	P10144	4Y1X	P09382	2L0F	P62979
1D5M	P01552	4PIH	P62987	1PWI	Q15109	1K8T	P40136	1L3P	Q40963	1IAU	P10144	4Y1Y	P09382	2L0T	P62979
1D5X	P01552	4PIJ	P62987	1S4J	P05387	1Y0V	P40136	1NLX	P43215	1GD8	Q9Z9H5	4Y1Z	P09382	6G18	P62979
1D5Z	P01552	4RF0	P62987	1UCP	Q9ULZ3	1KJB	P14151	1T5W	P27705	1GUV	Q13231	4Y20	P09382	2NZ2	P00966
1D6E	P01552	4RF1	P62987	1YCK	O75594	3CFW	P14151	1T5X	P27705	1HKI	Q13231	4Y22	P09382	2PA2	P27635
1G0Z	P01552	4S1Z	P62987	1YUR	Q99584	5VC1	P14151	1TWV	Q5SHN9	1J7V	Q13651	4Y24	P09382	2V1D	Q16695
1SBB	P01552	4XKL	P62987	2GFU	P52701	1M9J	P29474	5V8I	Q5SHN9	1LQS	Q13651	5MWT	P09382	2YBP	Q16695
1SE3	P01552	5G07	P62987	2HM2	Q8WXC3	1M9K	P29474	1U58	A2Q6L5	1Y6K	Q13651	5MWX	P09382	2YBS	Q16695
1SE4	P01552	5G08	P62987	2K2J	P16885	6CIF	P29474	1VLK	P03180	1Y6M	Q13651	6B94	P09382	3A6N	Q16695
1SEB	P01552	5G0B	P62987	2K45	P21579	1NSI	P35228	4V67	Q5SLQ1	1Y6N	Q13651	1IAT	P06744	3T6R	Q16695
2SEB	P01552	5G0C	P62987	2KR6	P49768	2NSI	P35228	3HY2	Q9BYN0	5IXI	Q13651	1IRI	P06744	4V2V	Q16695
3GP7	P01552	5G0D	P62987	2MP1	Q99731	3E7G	P35228	1YCK	O75594	1JBI	O43405	1JIQ	P06744	4V2W	Q16695
3R8B	P01552	5G0G	P62987	2N7Q	Q92542	3EJ8	P35228	2HZP	Q16719	1L3P	Q40963	1JLH	P06744	2VZ6	Q9UQM7
3SEB	P01552	5G0H	P62987	2NN6	Q06265	4CX7	P35228	3E9K	Q16719			1NUH	P06744	3SOA	Q9UQM7
3W2D	P01552	5G0I	P62987	2O6S	Q4G1L3	1SLC	P11116	2M0R	Q9HCY8			1ISF	Q10588	5IG3	Q9UQM7
4C56	P01552	5G0J	P62987	3A79	Q4G1L3	1SLT	P11116	2O6R	Q4G1L2			1ISG	Q10588	2YD0	Q9NZ08
4RGM	P01552	5G0K	P62987	4UIP	Q4G1L3	1SOR	Q6J8I9	2Z62	Q4G1L2			1ISH	Q10588	3MDJ	Q9NZ08
4RGN	P01552	5HPK	P62987	3THW	P43246	2B60	Q6J8I9	2Z63	Q4G1L2			1ISI	Q10588	3QNF	Q9NZ08
4RGO	P01552	5HPL	P62987	3THX	P43246	2B6P	Q6J8I9	6BXA	Q4G1L2			1ISJ	Q10588	3RJO	Q9NZ08
1EMR	P15018	5HPS	P62987	3THY	P43246	3M9I	Q6J8I9	6BXC	Q4G1L2			1ISM	Q10588	5J5E	Q9NZ08
1PVH	P15018	5HPT	P62987	3THZ	P43246	1T0J	Q13936	2PA2	P27635			1JXQ	P55211	2ZFY	Q96FW1
2Q7N	P15018	5J26	P62987	2PA2	P27635	5V2P	Q13936	5AJ0	P27635			2AR9	P55211	3VON	Q96FW1
1ENF	P0A0M0	5J8P	P62987	5AJ0	P27635	5V2Q	Q13936	4XCS	Q06830			1KWP	P49137	4DHZ	Q96FW1
1EWC	P0A0M0	5JBV	P62987	2RIG	P30123	1T5W	P27705	3E07	P48607			1LOX	P12530	4I6L	Q96FW1
1F77	P0A0M0	5JBY	P62987	2ZBJ	P58908	1T5X	P27705	4LXR	P48607			6BXI	Q15208	3AN2	P49450
1HXY	P0A0M0	2PA2	P27635	3B2D	O95711	1U58	A2Q6L5	4LXS	P48607			1QBH	Q13490	3NQJ	P49450
2XN9	P0A0M0	3BCH	P08865	4HWB	P78552	1VLK	P03180	3OQV	Q8GED7			1SLA	P11116	3NQU	P49450
2XNA	P0A0M0	6G4S	P08865	3FPR	P0C8E7	1W2C	P23677	4Q24	Q8GED7			1WDY	Q05823	3R45	P49450
1EOT	P51671	3VI6	P62888	3FPT	P0C8E7	1W2D	P23677	3R2V	F2Z275			1WLJ	Q96AZ6	3WTP	P49450
2EOT	P51671	4AOW	P63244	3OG4	Q8IU54	1W2F	P23677	4JRX	C5MK56			1X23	P61077	5CVD	P49450
2MPM	P51671	4BXF	P46776	3OG6	Q8IU54	1WMK	Q9UIK4	4JRY	C5MK56			1ZKK	P62805	6BUZ	P49450
1HTN	P05452	4CCM	P62917	5IXD	Q8IU57	1Z9X	Q9UIK4	6EVJ	H6QM92			2RVQ	P04908	6COW	P49450
1RJH	P05452	4CCN	P62917	5IXI	Q8IU57	1ZWS	Q9UIK4	6EVK	H6QM92			2D0T	P14902	3BCH	P08865
1TN3	P05452	4CCO	P62917	5L04	Q8IU57	2A27	Q9UIK4	4QRP	X2G898			2HOD	P35226	6G4S	P08865
3L9J	P05452	4Y30	P62917	5T5W	Q8IU57	2A2A	Q9UIK4	4QRQ	X2G898			3GS2	Q99496	3SE6	Q6P179
1HUL	P05113	4CXG	P62266	3RJR	P07200	2CKE	Q9UIK4	4QRS	Q9YRL3			2IIJ	Q71DI3	4E36	Q6P179
3QT2	P05113	4CXH	P62266	5VQF	P07200	1XQ8	P37840	4QRT	Q9YRL8			2K2J	P16885	4JBS	Q6P179
3VA2	P05113	6G4W	P62266	3S98	P17181	2JN5	P37840	4UAQ	P9WGP3			2KR6	P49768	5AB0	Q6P179
2IPK	Q03909	5A8L	P36578	4PO6	P17181	6H6B	P37840	4UGO	Q96L21			2KRE	O95155	5AB2	Q6P179

1IL7	P13232
3DI2	P13232
3DI3	P13232
1ILR	P18510
1ILT	P18510
1IRA	P18510
1IRP	P18510
1ITN	P18510
2IRT	P18510
1ITA	P01583
2ILA	P01583
2KKI	P01583
2L5X	P01583
5UC6	P01583
1JCK	POAOL5
1JWM	POAOL5
1JWS	POAOL5
1JE6	Q29980
1JV2	P05106
3E3Q	P01897
1M8A	P78556
1MKF	041925
1OBX	Q01344
1R5I	Q48898
1SYW	P68104
1T7V	P25311
1U5M	P02458
1VGK	P01902
1ZSD	P03206
2GT9	Q16655
2KUM	Q9Y4X3
2L4N	000585
2MGS	P42830
2MP1	Q99731
2NXY	Q49DS8
2NXZ	Q6P5S8
2PJV	P19551
2000	095150
2OQP	Q9HBE4
2Q8R	Q16627
2RA4	Q99616
2SAM	Q5QGH9
2X29	P41273
2XFX	Q30291
2YEZ	P21611
2YS0	P22413
3UP1	P16871
3FPR	POC8E7
3GBL	Q65XZ7
3IOZ	Q5QGG3
3IT8	Q9DHW0
3JTS	P19505
3JTT	Q30597
3LB6	Q14627

4XXB	P62913
6F4P	P62753
6F4Q	P62753
4V6W	P05388
1AU1	P01574
1AXM	P05230
1DJS	P05230
1DZC	P05230
1DZD	P05230
1E00	P05230
1EVT	P05230
1HKN	P05230
1JQZ	P05230
1JT3	P05230
1JT4	P05230
1JT5	P05230
1JT7	P05230
1JTC	P05230
1JY0	P05230
1K5U	P05230
1K5V	P05230
1M16	P05230
1NZK	P05230

3UX9	P01562
4CCA	Q15833
4P5I	Q2F862
4ZK9	Q2F862
4ZKC	Q2F862
4PJ1	P10809
4UG0	Q96L21
5LKS	Q96L21
5T2C	Q96L21
6EK0	Q96L21
5A2Q	P62701
5OA3	P62701
5VYC	P62701
6FEC	P62701
6G18	P62701
6G4S	P62701
6G4W	P62701
6G51	P62701
6G53	P62701
6G5H	P62701
6G5I	P62701
5A8L	P18621
4V6W	P05388
6D9J	P13639
4ZF7	A3FBE6
5M6U	000329
5UBT	000329
5VLR	000329

1YCK	075594
1YGR	P08575
1YGU	P08575
5FMV	P08575
5FN6	P08575
5FN7	P08575
2CQV	Q15746
2YR3	Q15746
2E80	Q9Y3Z3
3U1N	Q9Y3Z3
4BZB	Q9Y3Z3
4QG4	Q9Y3Z3
4RXO	Q9Y3Z3
3F31	Q13813
2H9R	P24588
2HQW	Q05586
2I4I	000571
2K36	P68466
2KAV	Q99250
2KBI	Q14524
3KF9	Q9H1R3
2M64	Q40960
2N80	P08138
2NYJ	035433
2OVC	P56696
2PA2	P27635
5AJ0	P27635
6B27	Q13698
2VN9	Q13557
2W2C	Q13557
3GP2	Q13557
5VLO	Q13557
6AYW	Q13557
3BJ4	P51787
3HFC	P51787
3HFE	P51787
3HBW	Q92913
5VMS	PODP24
5WSU	PODP24
5WSV	PODP24
3WA0	Q9Y4B6
4P7I	Q9Y4B6
5JK7	Q9Y4B6
4IGV	P85524
4IGW	P85524
4IHR	P85524
4UG0	Q96L21
6EK0	Q96L21
5A2Q	P62701
5A8L	P18621
4V6W	P05388
5CYW	P68598

4V6X	Q96L21
5LKS	Q96L21
5T2C	Q96L21
6EK0	Q96L21
5A2Q	P62701
5OA3	P62701
6G5I	P62701
5A8L	P18621
4V6W	P05388
5X03	P55788
5X04	P55788
8TFV	P55788

2L43	P84243
2N7Q	Q92542
2PA2	P27635
2QXG	P49862
4CAY	P06899
2SHP	Q06124
2UVL	Q13489
2ZBJ	P58908
3GC6	Q9TTF5
3HHS	044249
3KV4	Q6NXT2
5E1T	Q13077
3MWP	P13699
4AW9	Q99538
4CCA	Q15833
4EF4	Q86WV6
4EFO	Q9UHD2
4G1T	P09913
4KGH	Q9NP55
4UG0	Q96L21
5A2Q	P62701
5A8L	P18621
4V6W	P05388
5CFL	A7SLZ2

5CU5	Q6P179
5J6S	Q6P179
5K1V	Q6P179
3WAA	Q71UI9
4AOW	P63244
4CFG	Q14258
4LTB	Q14258
5EYA	Q14258
5FER	Q14258
5NT1	Q14258
5NT2	Q14258
6FLM	Q14258
6FLN	Q14258
4CXG	P62266
4CXH	P62266
6G4W	P62266
6F4P	P62753
6F4Q	P62753
5A8L	P18621
4V6W	P05388

3LQA	Q1PHM6
3O4O	P27930
3ON9	Q7TDW8
3PWU	P41355
3PWV	Q95477
3QQ3	Q9Q0U7
3QQ4	O19244
5H94	Q07717
3RWI	Q9GJ77
4NZD	Q9HBE5
3URF	O00300
3WEX	I2FL84

Compound 17	Compound 17	Compound 18	Compound 18	Compound 19	Compound 19	Compound 20	Compound 20
PDBID	UNIPROTKB	PDBID	UNIPROTKB	PDBID	UNIPROTKB	PDBID	UNIPROTKB
1A0N	P27986	1A1M	P61769	2SDF	P48061	1AKJ	P01892
1A4R	P60953	3HCS	Q9Y4K3	3GV3	P48061	1ALU	P05231
1BFI	P23727	1LNU	P14483	3HP3	P48061	1FYT	P01850
1CQ0	O43612	1MPV	Q96RJ3	4X5W	P04233	1FHH	P00268
1E96	P63000	1PX5	Q29599	5KSU	P04233	1BMG	P01888
1EXT	P19438	1A8M	P01375	5KSV	P04233	1BZI	P48023
1F9P	P02775	1AJW	P19803	6CQR	P01903	5ZC5	P00749
1GUV	Q13231	1AM4	Q07960	1AO7	A0A5B9	1C8P	P32927
1J7V	Q13651	1AZE	P62993	1BD2	A0A5B9	1EGJ	P32927
1N3K	Q9Z297	1B3T	P03211	2EYR	A0A5B9	1GH7	P32927
1PME	P28482	1BII	P01887	5HHO	A0A5B9	2GYS	P32927
1S4J	P05387	6MP1	P01887	5HYJ	A0A5B9	2NA8	P32927
1T5W	P27705	2N80	P52565	5KS9	A0A5B9	2NA9	P32927
1U58	A2Q6L5	1CDH	P01730	5KSA	A0A5B9	4NKQ	P32927
1VLK	P03180	1CDI	P01730	5KSB	A0A5B9	5DWU	P32927
1WDZ	Q9UQB8	6MEO	P01730	6CPH	A0A5B9	6BNK	P11609
1YCK	O75594	6MET	P01730	1KGC	P01848	4I9X	O14763
2CQL	P32969	1CEE	P42768	1MI5	P01848	4N90	O14763
2ED0	Q9NYB9	1EJ5	P42768	5JZI	P01848	4OD2	O14763
2ENQ	P42336	2OT0	P42768	5NQK	P01848	1D2Q	P50591
2LJ5	P62987	1WSO	O43612	1B2T	P78423	1DG6	P50591
2NBE	P62987	4NKQ	P04141	1F2L	P78423	5CIR	P50591
2RSU	P62987	4OTH	Q16512	3KBX	P10147	1EL0	P22362
4HJK	P62987	4OTI	Q16512	4RA8	P10147	4OIJ	P22362
4JIO	P62987	1E0A	P35465	4ZKB	P10147	4OIK	P22362
4P4H	P62987	2DMO	P19878	5COR	P10147	1E08	P03437
4PIG	P62987	1EES	Q61036	5D65	P10147	1HA0	P03437
4PIH	P62987	1EIA	P69732	1BO0	P80098	1HGG	P03437
4PIJ	P62987	1HEK	P69732	1NCV	P80098	1HTM	P03437
3PWL	P61769	2BL6	P69732	4ZKC	P80098	3VUN	P03437
3QZW	P61769	2EIA	P69732	1CE4	P20871	4C56	P03437
3REW	P61769	2K84	P69732	2ESX	P20871	4HMG	P03437
3RL1	P61769	1EXT	P19438	2ESZ	P20871	5HMG	P03437
3RL2	P61769	1F5X	P27870	4R2G	P20871	1F42	P29460
3RWJ	P61769	1F9P	P02775	1CM9	Q98157	1F45	P29460
3S6C	P61769	1FHA	P02794	1DN3	P02776	5NJD	P29460
3SDX	P61769	1FOE	Q60610	1HUM	P13236	1G0Y	P14778
3SJV	P61769	1G4U	P74873	1JH5	Q9Y275	1IRA	P14778
3SKM	P61769	1YYL	P35961	1JMA	Q92956	6ATI	P08670
5J8P	P62987	1GA3	P35225	1L8J	Q9UNN8	6BIR	P08670

5JBV	P62987	1GUV	Q13231	1LB4	Q9Y4K3	1HDM	P28067
5JBY	P62987	1GZS	O52623	4NQE	P01850	2BC4	P28067
2ZOQ	P27361	1HE1	Q51451	1B13	P00268	4FQX	P28067
4QTB	P27361	1HIB	P01584	4RWO	Q29599	4GBX	P28067
3N3U	Q06277	1I49	P53365	1WH3	Q15646	4IOP	P28068
3QIK	Q8TCU6	1IAR	P24394	1ZT4	P15813	1HTI	P60174
5D27	Q8TCU6	1ICW	P10145	2G47	P14735	1KLG	P60174
5D3V	Q8TCU6	1J7V	Q13651	3N57	P14735	1KLU	P60174
5D3W	Q8TCU6	1JU5	P46108	3OFI	P14735	3QB7	P31785
5D3X	Q8TCU6	1KI1	Q15811	3QZ2	P14735	4GS7	P31785
5D3Y	Q8TCU6	1KZ7	Q64096	4DTT	P14735	5M5E	P31785
3VI6	P62888	1NF3	Q9JK83	4DWK	P14735	6E8K	P14784
4BXF	P46776	1NTY	O75962	4GS8	P14735	1IRL	P60568
4CCM	P62917	1S9I	P36507	4GSC	P14735	1M47	P60568
4CCN	P62917	1T5W	P27705	4GSF	P14735	1M48	P60568
4CCO	P62917	1TJT	Q08043	4IFH	P14735	1M49	P60568
4Y3O	P62917	1VLK	P03180	4IOF	P14735	1M4A	P60568
4RJ9	Q6YWF1	1WG7	Q9BZ29	4LTE	P14735	5KSA	P01909
5A8L	P18621	1YCK	O75594	4M1C	P14735	5KSB	P01909
4XXB	P62913	2C30	Q9NQU5	4NXO	P14735	5KSU	P01909
4V6W	P05388	2CRH	P15498	4PES	P14735	4KPO	P23946
6D9J	P13639	2DFK	Q9QX73	4PF7	P14735	5YJM	P23946
5U5S	P40763	2FJU	Q00722	4PF9	P14735	5YJP	P23946
6G5I	P42677	2H7O	Q05608	4PFC	P14735	1LNU	P14434
5A2Q	P46781	2HW6	Q9BUB5	4QIA	P14735	1MUJ	P14434
6F4P	P62753	2JPH	O43157	4RE9	P14735	3C5Z	P14434
6F4Q	P62753	2RQR	Q92608	5CJO	P14735	3TJV	P20718
1CT6	P68431	2VOU	O49003	5UOE	P14735	3TK9	P20718
4UPO	P68431	4HWB	P78552	5WOB	P14735	4GAW	P20718
1DS6	P52566	3BYH	P60709	6B3Q	P14735	1M73	P00491
2W2T	P15153	3GCG	Q9R8E4	6B70	P14735	1PF7	P00491
1DYT	P12724	3ME2	O35305	6B7Y	P14735	1PWY	P00491
1F66	P84233	3RYT	P70206	6B7Z	P14735	5YY4	P51681
1FEW	Q9NR28	3URF	O14788	6BF6	P14735	6FGP	P51681
1GZW	P09382	3VHL	Q8C147	6BF7	P14735	6MEO	P51681
1IAT	P06744	4DID	O30916	6BF8	P14735	6MET	P51681
1ISF	Q10588	3QBY	P62805	6BF9	P14735	1OC0	P04004
1JXQ	P55211	2CV5	P04908	6BFC	P14735	1S4G	P04004
1KWP	P49137	2D0T	P14902	3ODU	P61073	1SSU	P04004
1LOX	P12530	2H0D	P35226	3OE0	P61073	2JQ8	P04004
1PSB	Q15208	3GS2	Q99496	3OE6	P61073	1TJG	Q75760
1QBH	Q13490	2IIJ	Q71DI3	3OE8	P61073	2B4C	Q75760
1SLA	P11116	2K2J	P16885	3OE9	P61073	2F5B	Q75760
1WDY	Q05823	2W2X	P16885	2MS7	Q7Z434	4JM2	Q75760
1WLJ	Q96AZ6	2KR6	P49768	2MS8	Q7Z434	5FYK	Q75760
1X23	P61077	5FN4	P49768	2VGQ	Q7Z434	1TNR	P01374
1ZKK	P62805	2KRE	O95155	3J6C	Q7Z434	4MXV	P01374
2RVQ	P04908	2L43	P84243	3J6J	Q7Z434	4MXW	P01374
2D0T	P14902	2N7Q	Q92542	3RC5	Q7Z434	3CC5	P40967
2H0D	P35226	2PA2	P27635	4P4H	Q7Z434	4IS6	P40967
3GS2	Q99496	2QXG	P49862	5JEK	Q7Z434	1W63	P35585
2IIJ	Q71DI3	4CAY	P06899	2NNA	P18573	4HMY	P35585
2K2J	P16885	2SHP	Q06124	4GG6	P18573	4P6Z	P35585
2KR6	P49768	2UVL	Q13489	4OZF	P18573	6CM9	P35585

2KRE	O95155	2ZBJ	P58908	4OZG	P18573	6CRI	P35585
2L43	P84243	3GC6	Q9TTF5	4OZH	P18573	6D83	P35585
2N7Q	Q92542	3HHS	O44249	4OZI	P18573	6D84	P35585
2PA2	P27635	3KV4	Q6NXT2	4Z7U	P18573	6DFF	P35585
2QXG	P49862	5E1T	Q13077	4Z7V	P18573	1YWH	Q03405
4CAY	P06899	3MWP	P13699	4Z7W	P18573	3U74	Q03405
2SHP	Q06124	3RM1	Q5E997	2VXS	Q16552	4QTI	Q03405
6CMS	Q06124	4AW9	Q99538	4HR9	Q16552	2ERS	Q13261
6CRF	Q06124	4BVU	Q99PZ6	4HSA	Q16552	2Z3Q	Q13261
6CRG	Q06124	4CCA	Q15833	4QHU	Q16552	2Z3R	Q13261
2UVL	Q13489	4EF4	Q86WV6	5HHV	Q16552	2Q6W	P79483
3EB5	Q13489	4EFO	Q9UHD2	5HHX	Q16552	3C5J	P79483
3EB6	Q13489	4G1T	P09913	5HI3	Q16552	4H1L	P79483
3MOA	Q13489	4KEG	Q9NP55	5HI4	Q16552	2XQB	P40933
3MOD	Q13489	4UGO	Q96L21	5HI5	Q16552	4H25	B8YAC7
2ZBJ	P58908	5A2Q	P62701	5N7W	Q16552	4H26	B8YAC7
3GC6	Q9TTF5	5A8L	P18621	5N92	Q16552	3JVF	Q96F46
3GH3	Q9TTF5	4V6W	P05388	5NAN	Q16552	4HSA	Q96F46
3GHH	Q9TTF5	5CFL	A7SLZ2	5VB9	Q16552	4NUX	Q96F46
3KOU	Q9TTF5	4V5R	Q5SHN7	2WRY	O73909	5N9B	Q96F46
3P5S	Q9TTF5	1FJG	Q5SHP2	3NJ5	O73909	5NAN	Q96F46
3HHS	O44249	1QD7	Q5SHQ5	4X37	O73909	3K51	O95407
3KV4	Q6NXT2	1G1X	Q5SJ76	4X38	O73909	3MHD	O95407
5BWN	Q6NXT2	2KJV	Q5SLP8	4X39	O73909	3MI8	O95407
5BWO	Q6NXT2	1X18	Q5SLQ0	4X3A	O73909	4J6G	O95407
5F6K	Q6NXT2	1FQ3	P10144	3JTT	Q6V7J5	4KGG	O95407
5I3L	Q6NXT2	1GD8	Q9Z9H5	3RWC	Q6V7J5	4KGQ	O95407
5E1T	Q13077	1JBI	O43405	3RWD	Q6V7J5	3O4O	Q9NPH3
5H10	Q13077	1L3P	Q40963	3RWE	Q6V7J5	4D8P	L8E864
3MWP	P13699	1NLX	P43215	3RWF	Q6V7J5	5KS9	L8E864
3MWT	P13699	1TWV	Q5SHN9	3RWG	Q6V7J5	4DOH	Q6UXL0
3MX2	P13699	4V67	Q5SLQ1	3RWH	Q6V7J5	6DF3	Q6UXL0
3MX5	P13699	1XW3	Q9BYN0	3RWI	Q6V7J5	4GRL	Q30066
3Q7B	P13699	2HZP	Q16719	4ZFZ	Q6V7J5	4MAY	Q30066
3Q7C	P13699	2MOR	Q9HCY8	3LQZ	P04440	4HSV	P10720
4FVU	P13699	2O6R	Q4G1L2	4P4R	P04440	4HT1	O43508
4G9Z	P13699	4XCS	Q06830	4P57	P04440	4IG8	P00973
4GV3	P13699	3E07	P48607	4P5K	P04440	4JQV	P30466
4GV6	P13699	3OQV	Q8GED7	4P5M	P04440	4XXC	P30466
4GV9	P13699	3R2V	F2Z275	3NGB	Q0ED31	4JQX	Q3KSS8
3RM1	Q5E997	4NFZ	H6QM92	3SE8	Q0ED31	4KKN	Q28090
4AW9	Q99538	4UAQ	P9WGP3	4YDK	Q0ED31	4LFH	Q6PJ56
4AWA	Q99538	8TFV	P55788	4YDL	Q0ED31	4LHU	Q6PJ56
4CCA	Q15833			4YFL	Q0ED31	4MNH	Q6PJ56
4EF4	Q86WV6			4ENO	O43557	4NDM	Q6PJ56
4EFO	Q9UHD2			4J6G	O43557	4MD4	P16112
4G1T	P09913			4KG8	O43557	4MHE	P55774
4KEG	Q9NP55			4KGG	O43557	4N8P	F6ZMI5
5CFL	A7SLZ2			4KGQ	O43557		
4V5R	Q5SHN7			4NKQ	P15509		
1FJG	Q5SHP2			4RS1	P15509		
1QD7	Q5SHQ5			4P5I	Q2F862		
1G1X	Q5SJ76			4ZK9	Q2F862		
2KJV	Q5SLP8			4R4H	Q73372		

2KJW	Q5SLP8
3ZZP	Q5SLP8
1X18	Q5SLQ0
1FQ3	P10144
1IAU	P10144
1GD8	Q9Z9H5
1JBI	O43405
1L3P	Q40963
1NLX	P43215
1TWV	Q5SHN9
5V8I	Q5SHN9
4V67	Q5SLQ1
1XW3	Q9BYN0
1XW4	Q9BYN0
1YZS	Q9BYN0
2B6F	Q9BYN0
2RII	Q9BYN0
3CYI	Q9BYN0
3HY2	Q9BYN0
2HZP	Q16719
3E9K	Q16719
2MOR	Q9HCY8
2O6R	Q4G1L2
4BV4	Q4G1L2
4QDH	Q4G1L2
4QXE	Q4G1L2
4QXF	Q4G1L2
5GY2	Q4G1L2
5IJB	Q4G1L2
5IJC	Q4G1L2
5IJD	Q4G1L2
6BXA	Q4G1L2
6BXC	Q4G1L2
4XCS	Q06830
3E07	P48607
4LXR	P48607
4LXS	P48607
3OQV	Q8GED7
4Q24	Q8GED7
3R2V	F2Z275
4JRX	C5MK56
4JRY	C5MK56
4PRA	C5MK56
4PRB	C5MK56
6EVJ	H6QM92
6EVK	H6QM92
4PRN	Q1HVF7
4QRP	X2G898
4QRQ	X2G898
4QRS	Q9YRL3
4QRT	Q9YRL8
4UAQ	P9WGP3
6GD5	P55788
5U3K	Q73372
5U3L	Q73372
5U3M	Q73372
5U3N	Q73372
5U3O	Q73372
4S3N	Q9Y6K5
5UJT	O19707
4ZF7	A3FBE6
4ZG6	Q13822
4ZG7	Q13822
4ZG9	Q13822
4ZGA	Q13822
5KXA	Q13822
5M7M	Q13822
5MHP	Q13822

Table S2 A total of overlapping 1258 UniprotKB markers yo twenty compounds

Compound Name	UnipriotKB
1 2’ -O-(2-methylbutyryl) -isoswertisin	P0CY27
1 2’ -O-(2-methylbutyryl) -isoswertisin	P03404
1 2’ -O-(2-methylbutyryl) -isoswertisin	P05112
1 2’ -O-(2-methylbutyryl) -isoswertisin	Q9EPW9
1 2’ -O-(2-methylbutyryl) -isoswertisin	Q9Y2C9
1 2’ -O-(2-methylbutyryl) -isoswertisin	P68638
1 2’ -O-(2-methylbutyryl) -isoswertisin	Q89859
1 2’ -O-(2-methylbutyryl) -isoswertisin	Q20NS3
1 2’ -O-(2-methylbutyryl) -isoswertisin	Q05315
1 2’ -O-(2-methylbutyryl) -isoswertisin	P02671
1 2’ -O-(2-methylbutyryl) -isoswertisin	P02675
1 2’ -O-(2-methylbutyryl) -isoswertisin	P02676
1 2’ -O-(2-methylbutyryl) -isoswertisin	P02679
1 2’ -O-(2-methylbutyryl) -isoswertisin	P01871
1 2’ -O-(2-methylbutyryl) -isoswertisin	Q51918
1 2’ -O-(2-methylbutyryl) -isoswertisin	P27918
1 2’ -O-(2-methylbutyryl) -isoswertisin	P01700
1 2’ -O-(2-methylbutyryl) -isoswertisin	P01824
1 2’ -O-(2-methylbutyryl) -isoswertisin	P01880
1 2’ -O-(2-methylbutyryl) -isoswertisin	P0DOX3
1 2’ -O-(2-methylbutyryl) -isoswertisin	Q2UVX4
1 2’ -O-(2-methylbutyryl) -isoswertisin	P01721
1 2’ -O-(2-methylbutyryl) -isoswertisin	P0CG04
1 2’ -O-(2-methylbutyryl) -isoswertisin	P01772
1 2’ -O-(2-methylbutyryl) -isoswertisin	Q6PYX1
1 2’ -O-(2-methylbutyryl) -isoswertisin	Q9Y5Y6
1 2’ -O-(2-methylbutyryl) -isoswertisin	P08603
1 2’ -O-(2-methylbutyryl) -isoswertisin	P00751
1 2’ -O-(2-methylbutyryl) -isoswertisin	A0A0H3K6Z8
1 2’ -O-(2-methylbutyryl) -isoswertisin	P07358
1 2’ -O-(2-methylbutyryl) -isoswertisin	D3JIB2
1 2’ -O-(2-methylbutyryl) -isoswertisin	Q91132
1 2’ -O-(2-methylbutyryl) -isoswertisin	P01647

1 2’ -O-(2-methylbutyryl) -isoswertisin	P01808
1 2’ -O-(2-methylbutyryl) -isoswertisin	P01597
1 2’ -O-(2-methylbutyryl) -isoswertisin	Q5NV90
1 2’ -O-(2-methylbutyryl) -isoswertisin	P14210
1 2’ -O-(2-methylbutyryl) -isoswertisin	U3KM01
1 2’ -O-(2-methylbutyryl) -isoswertisin	P00746
1 2’ -O-(2-methylbutyryl) -isoswertisin	P13671
1 2’ -O-(2-methylbutyryl) -isoswertisin	O00187
1 2’ -O-(2-methylbutyryl) -isoswertisin	P0C0L4
1 2’ -O-(2-methylbutyryl) -isoswertisin	P01024
1 2’ -O-(2-methylbutyryl) -isoswertisin	P01594
1 2’ -O-(2-methylbutyryl) -isoswertisin	P01764
1 2’ -O-(2-methylbutyryl) -isoswertisin	P25791
1 2’ -O-(2-methylbutyryl) -isoswertisin	P01593
1 2’ -O-(2-methylbutyryl) -isoswertisin	S6C4R2
1 2’ -O-(2-methylbutyryl) -isoswertisin	Q91MA7
1 2’ -O-(2-methylbutyryl) -isoswertisin	S6C4S0
1 2’ -O-(2-methylbutyryl) -isoswertisin	P03435
1 2’ -O-(2-methylbutyryl) -isoswertisin	R9U684
1 2’ -O-(2-methylbutyryl) -isoswertisin	P01619
1 2’ -O-(2-methylbutyryl) -isoswertisin	Q8JDI3
1 2’ -O-(2-methylbutyryl) -isoswertisin	S6B291
1 2’ -O-(2-methylbutyryl) -isoswertisin	P01709
1 2’ -O-(2-methylbutyryl) -isoswertisin	A0A158RFT3
1 2’ -O-(2-methylbutyryl) -isoswertisin	A0A158RFT4
1 2’ -O-(2-methylbutyryl) -isoswertisin	A0A158RFT5
1 2’ -O-(2-methylbutyryl) -isoswertisin	Q5YD59
1 2’ -O-(2-methylbutyryl) -isoswertisin	P01031
1 2’ -O-(2-methylbutyryl) -isoswertisin	V9QIE5
1 2’ -O-(2-methylbutyryl) -isoswertisin	P0DOY2
1 2’ -O-(2-methylbutyryl) -isoswertisin	Q1HSF8
1 2’ -O-(2-methylbutyryl) -isoswertisin	P06312
1 2’ -O-(2-methylbutyryl) -isoswertisin	P01825
1 2’ -O-(2-methylbutyryl) -isoswertisin	P0DOY3
1 2’ -O-(2-methylbutyryl) -isoswertisin	Q9WUD1

1	2’ -O-(2-methylbutyryl) -isoswertisin	Q13404
1	2’ -O-(2-methylbutyryl) -isoswertisin	P52564
1	2’ -O-(2-methylbutyryl) -isoswertisin	Q8VSD5
1	2’ -O-(2-methylbutyryl) -isoswertisin	O76064
1	2’ -O-(2-methylbutyryl) -isoswertisin	Q15819
1	2’ -O-(2-methylbutyryl) -isoswertisin	P61088
1	2’ -O-(2-methylbutyryl) -isoswertisin	P27635
1	2’ -O-(2-methylbutyryl) -isoswertisin	P22090
1	2’ -O-(2-methylbutyryl) -isoswertisin	P61086
1	2’ -O-(2-methylbutyryl) -isoswertisin	Q8NC51
1	2’ -O-(2-methylbutyryl) -isoswertisin	Q96L21
1	2’ -O-(2-methylbutyryl) -isoswertisin	P62701
1	2’ -O-(2-methylbutyryl) -isoswertisin	P05388
1	2’ -O-(2-methylbutyryl) -isoswertisin	P18077
1	2’ -O-(2-methylbutyryl) -isoswertisin	P18124
1	2’ -O-(2-methylbutyryl) -isoswertisin	P18621
1	2’ -O-(2-methylbutyryl) -isoswertisin	P26373
1	2’ -O-(2-methylbutyryl) -isoswertisin	P30050
1	2’ -O-(2-methylbutyryl) -isoswertisin	Q1HVF7
1	2’ -O-(2-methylbutyryl) -isoswertisin	C5MK56
1	2’ -O-(2-methylbutyryl) -isoswertisin	P03211
1	2’ -O-(2-methylbutyryl) -isoswertisin	X2G898
1	2’ -O-(2-methylbutyryl) -isoswertisin	P30685
1	2’ -O-(2-methylbutyryl) -isoswertisin	Q9YRL3
1	2’ -O-(2-methylbutyryl) -isoswertisin	Q9YRL8
1	2’ -O-(2-methylbutyryl) -isoswertisin	Q13619
1	2’ -O-(2-methylbutyryl) -isoswertisin	Q16531
1	2’ -O-(2-methylbutyryl) -isoswertisin	P33064
1	2’ -O-(2-methylbutyryl) -isoswertisin	P0C569
1	2’ -O-(2-methylbutyryl) -isoswertisin	P07239
2	2’ -O-(3,4-dimethoxybenzoyl)vitexin	P80372
2	2’ -O-(3,4-dimethoxybenzoyl)vitexin	P80373
2	2’ -O-(3,4-dimethoxybenzoyl)vitexin	P80374
2	2’ -O-(3,4-dimethoxybenzoyl)vitexin	P80376
2	2’ -O-(3,4-dimethoxybenzoyl)vitexin	P80377

2	2’-O-(3,4-dimethoxybenzoyl)vitexin	P80380
2	2’-O-(3,4-dimethoxybenzoyl)vitexin	Q5SHN3
2	2’-O-(3,4-dimethoxybenzoyl)vitexin	Q5SHN7
2	2’-O-(3,4-dimethoxybenzoyl)vitexin	Q5SHN8
2	2’-O-(3,4-dimethoxybenzoyl)vitexin	Q5SHN9
2	2’-O-(3,4-dimethoxybenzoyl)vitexin	Q5SHP0
2	2’-O-(3,4-dimethoxybenzoyl)vitexin	Q5SHP2
2	2’-O-(3,4-dimethoxybenzoyl)vitexin	Q5SHP3
2	2’-O-(3,4-dimethoxybenzoyl)vitexin	Q5SHP6
2	2’-O-(3,4-dimethoxybenzoyl)vitexin	Q5SHP8
2	2’-O-(3,4-dimethoxybenzoyl)vitexin	Q5SHP9
2	2’-O-(3,4-dimethoxybenzoyl)vitexin	Q5SHQ0
2	2’-O-(3,4-dimethoxybenzoyl)vitexin	Q5SHQ4
2	2’-O-(3,4-dimethoxybenzoyl)vitexin	Q5SHQ5
2	2’-O-(3,4-dimethoxybenzoyl)vitexin	Q5SHQ6
2	2’-O-(3,4-dimethoxybenzoyl)vitexin	Q5SHQ7
2	2’-O-(3,4-dimethoxybenzoyl)vitexin	Q5SHR2
2	2’-O-(3,4-dimethoxybenzoyl)vitexin	Q5SHZ1
2	2’-O-(3,4-dimethoxybenzoyl)vitexin	Q5SIH3
2	2’-O-(3,4-dimethoxybenzoyl)vitexin	Q5SJ76
2	2’-O-(3,4-dimethoxybenzoyl)vitexin	Q5SJE1
2	2’-O-(3,4-dimethoxybenzoyl)vitexin	Q5SJH3
2	2’-O-(3,4-dimethoxybenzoyl)vitexin	Q5SKU1
2	2’-O-(3,4-dimethoxybenzoyl)vitexin	Q5SLP8
2	2’-O-(3,4-dimethoxybenzoyl)vitexin	Q5SLQ0
2	2’-O-(3,4-dimethoxybenzoyl)vitexin	Q5SLQ1
2	2’-O-(3,4-dimethoxybenzoyl)vitexin	Q9Z9H5
2	2’-O-(3,4-dimethoxybenzoyl)vitexin	P55788
2	2’-O-(3,4-dimethoxybenzoyl)vitexin	P10144
2	2’-O-(3,4-dimethoxybenzoyl)vitexin	Q9BYN0
2	2’-O-(3,4-dimethoxybenzoyl)vitexin	Q06830
2	2’-O-(3,4-dimethoxybenzoyl)vitexin	P08953
2	2’-O-(3,4-dimethoxybenzoyl)vitexin	P48607
2	2’-O-(3,4-dimethoxybenzoyl)vitexin	Q4G1L2
2	2’-O-(3,4-dimethoxybenzoyl)vitexin	F2Z275

2 2’-O-(3,4-dimethoxybenzoyl)vitexin	H6QM90
2 2’-O-(3,4-dimethoxybenzoyl)vitexin	H6QM91
2 2’-O-(3,4-dimethoxybenzoyl)vitexin	H6QM92
2 2’-O-(3,4-dimethoxybenzoyl)vitexin	P08253
2 2’-O-(3,4-dimethoxybenzoyl)vitexin	P16035
2 2’-O-(3,4-dimethoxybenzoyl)vitexin	O43405
2 2’-O-(3,4-dimethoxybenzoyl)vitexin	P9WGP3
2 2’-O-(3,4-dimethoxybenzoyl)vitexin	Q16719
2 2’-O-(3,4-dimethoxybenzoyl)vitexin	P12429
2 2’-O-(3,4-dimethoxybenzoyl)vitexin	Q8GED7
2 2’-O-(3,4-dimethoxybenzoyl)vitexin	Q9HCY8
2 2’-O-(3,4-dimethoxybenzoyl)vitexin	Q40963
2 2’-O-(3,4-dimethoxybenzoyl)vitexin	P43215
2 2’-O-(3,4-dimethoxybenzoyl)vitexin	Q8NC51
2 2’-O-(3,4-dimethoxybenzoyl)vitexin	Q96L21
2 2’-O-(3,4-dimethoxybenzoyl)vitexin	P62701
2 2’-O-(3,4-dimethoxybenzoyl)vitexin	P05388
2 2’-O-(3,4-dimethoxybenzoyl)vitexin	P18077
2 2’-O-(3,4-dimethoxybenzoyl)vitexin	P18124
2 2’-O-(3,4-dimethoxybenzoyl)vitexin	P18621
2 2’-O-(3,4-dimethoxybenzoyl)vitexin	P26373
2 2’-O-(3,4-dimethoxybenzoyl)vitexin	P27635
2 2’-O-(3,4-dimethoxybenzoyl)vitexin	P30050
2 2’-O-(3,4-dimethoxybenzoyl)vitexin	P27705
2 2’-O-(3,4-dimethoxybenzoyl)vitexin	P19438
2 2’-O-(3,4-dimethoxybenzoyl)vitexin	P02775
2 2’-O-(3,4-dimethoxybenzoyl)vitexin	A2Q6L5
2 2’-O-(3,4-dimethoxybenzoyl)vitexin	E9PMV2
2 2’-O-(3,4-dimethoxybenzoyl)vitexin	O43612
2 2’-O-(3,4-dimethoxybenzoyl)vitexin	Q13231
2 2’-O-(3,4-dimethoxybenzoyl)vitexin	P03180
2 2’-O-(3,4-dimethoxybenzoyl)vitexin	Q13651
2 2’-O-(3,4-dimethoxybenzoyl)vitexin	O75594
2 2’-O-(3,4-dimethoxybenzoyl)vitexin	Q1HVF7
2 2’-O-(3,4-dimethoxybenzoyl)vitexin	C5MK56

2	2’ -O-(3,4-dimethoxybenzoyl)vitexin	P03211
2	2’ -O-(3,4-dimethoxybenzoyl)vitexin	X2G898
2	2’ -O-(3,4-dimethoxybenzoyl)vitexin	P30685
2	2’ -O-(3,4-dimethoxybenzoyl)vitexin	Q9YRL3
2	2’ -O-(3,4-dimethoxybenzoyl)vitexin	Q9YRL8
3	Acacetin	Q98Y46
3	Acacetin	Q8URG0
3	Acacetin	Q70A61
3	Acacetin	O11822
3	Acacetin	P26590
3	Acacetin	P27797
3	Acacetin	P16104
3	Acacetin	Q8N423
3	Acacetin	P16780
3	Acacetin	P01899
3	Acacetin	O19626
3	Acacetin	P33260
3	Acacetin	Q70AA1
3	Acacetin	Q9MYI6
3	Acacetin	Q1KW74
3	Acacetin	Q9IDV9
3	Acacetin	P01849
3	Acacetin	P30123
3	Acacetin	P04213
3	Acacetin	Q9NPA0
3	Acacetin	P03485
3	Acacetin	P04583
3	Acacetin	Q33CG5
3	Acacetin	A2KD59
3	Acacetin	P60201
3	Acacetin	Q70XD7
3	Acacetin	Q0QI92
3	Acacetin	P01579
3	Acacetin	Q66793
3	Acacetin	Q8WVV4

3	Acacetin	Q86UW6
3	Acacetin	P43355
3	Acacetin	P10619
3	Acacetin	Q56H30
3	Acacetin	P10321
3	Acacetin	P30464
3	Acacetin	P13747
3	Acacetin	P26715
3	Acacetin	Q13241
3	Acacetin	P47871
3	Acacetin	P08560
3	Acacetin	Q5GL29
3	Acacetin	P03204
3	Acacetin	P35222
3	Acacetin	P30305
3	Acacetin	Q9Y4H2
3	Acacetin	Q92619
3	Acacetin	Q9YYH6
3	Acacetin	P06725
3	Acacetin	Q04771
3	Acacetin	P38110
3	Acacetin	P59596
3	Acacetin	P59595
3	Acacetin	P78358
3	Acacetin	P17693
3	Acacetin	Q95HB9
3	Acacetin	P07910
3	Acacetin	P30479
3	Acacetin	Q13126
3	Acacetin	Q01668
3	Acacetin	O00238
3	Acacetin	Q2F4V2
3	Acacetin	Q1WDM0
3	Acacetin	P02771
3	Acacetin	Q03463

3	Acacetin	Q9DIT6
3	Acacetin	Q92959
3	Acacetin	Q3KSU1
3	Acacetin	Q8WLS4
3	Acacetin	P0C6H2
3	Acacetin	P01732
3	Acacetin	P13285
3	Acacetin	Q9YV12
3	Acacetin	P04439
3	Acacetin	P15812
3	Acacetin	Q3KST2
3	Acacetin	Q9QDK7
3	Acacetin	Q49PI7
3	Acacetin	Q692E0
3	Acacetin	E0YFW1
3	Acacetin	Q8NC51
3	Acacetin	Q96L21
3	Acacetin	P62701
3	Acacetin	P05388
3	Acacetin	P18077
3	Acacetin	P18124
3	Acacetin	P18621
3	Acacetin	P26373
3	Acacetin	P27635
3	Acacetin	P30050
3	Acacetin	P0AEX9
3	Acacetin	Q9NP55
3	Acacetin	P12724
3	Acacetin	Q6NXT2
3	Acacetin	P84243
3	Acacetin	Q71DI3
3	Acacetin	P04908
3	Acacetin	P06899
3	Acacetin	P62805
3	Acacetin	P68431

3	Acacetin	P55211
3	Acacetin	Q9NR28
4	acacetin-7-O-β -D-glucoside	Q6SW59
4	acacetin-7-O-β -D-glucoside	P62318
4	acacetin-7-O-β -D-glucoside	P00403
4	acacetin-7-O-β -D-glucoside	Q9WPU2
4	acacetin-7-O-β -D-glucoside	Q9YYU3
4	acacetin-7-O-β -D-glucoside	P12499
4	acacetin-7-O-β -D-glucoside	P30490
4	acacetin-7-O-β -D-glucoside	P04603
4	acacetin-7-O-β -D-glucoside	Q77378
4	acacetin-7-O-β -D-glucoside	P68979
4	acacetin-7-O-β -D-glucoside	K7N5M4
4	acacetin-7-O-β -D-glucoside	Q9YXL3
4	acacetin-7-O-β -D-glucoside	Q9YXL6
4	acacetin-7-O-β -D-glucoside	P42345
4	acacetin-7-O-β -D-glucoside	P0C213
4	acacetin-7-O-β -D-glucoside	P03366
4	acacetin-7-O-β -D-glucoside	P01012
4	acacetin-7-O-β -D-glucoside	P01901
4	acacetin-7-O-β -D-glucoside	Q8QMP2
4	acacetin-7-O-β -D-glucoside	Q9YXY3
4	acacetin-7-O-β -D-glucoside	P40126
4	acacetin-7-O-β -D-glucoside	P01891
4	acacetin-7-O-β -D-glucoside	Q70626
4	acacetin-7-O-β -D-glucoside	P30481
4	acacetin-7-O-β -D-glucoside	Q9QAC5
4	acacetin-7-O-β -D-glucoside	P17763
4	acacetin-7-O-β -D-glucoside	P30484
4	acacetin-7-O-β -D-glucoside	P0CF51
4	acacetin-7-O-β -D-glucoside	Q9YYU8
4	acacetin-7-O-β -D-glucoside	P68043
4	acacetin-7-O-β -D-glucoside	Q9Q0U8
4	acacetin-7-O-β -D-glucoside	K7N5M3
4	acacetin-7-O-β -D-glucoside	P18464

4	acacetin-7-O-β -D-glucoside	R4WL38
4	acacetin-7-O-β -D-glucoside	H2RG00
4	acacetin-7-O-β -D-glucoside	O14746
4	acacetin-7-O-β -D-glucoside	P02768
4	acacetin-7-O-β -D-glucoside	P62942
4	acacetin-7-O-β -D-glucoside	P43631
4	acacetin-7-O-β -D-glucoside	Q76ZQ3
4	acacetin-7-O-β -D-glucoside	Q9BZL6
4	acacetin-7-O-β -D-glucoside	Q8NHL6
4	acacetin-7-O-β -D-glucoside	P33241
4	acacetin-7-O-β -D-glucoside	Q01433
4	acacetin-7-O-β -D-glucoside	P03466
4	acacetin-7-O-β -D-glucoside	Q07FI1
4	acacetin-7-O-β -D-glucoside	D9J353
4	acacetin-7-O-β -D-glucoside	P30505
4	acacetin-7-O-β -D-glucoside	P30475
4	acacetin-7-O-β -D-glucoside	Q3KSS4
4	acacetin-7-O-β -D-glucoside	Q1HVF7
4	acacetin-7-O-β -D-glucoside	C5MK56
4	acacetin-7-O-β -D-glucoside	P03211
4	acacetin-7-O-β -D-glucoside	X2G898
4	acacetin-7-O-β -D-glucoside	P30685
4	acacetin-7-O-β -D-glucoside	Q9YRL3
4	acacetin-7-O-β -D-glucoside	Q9YRL8
4	acacetin-7-O-β -D-glucoside	P03169
4	acacetin-7-O-β -D-glucoside	P30460
4	acacetin-7-O-β -D-glucoside	P04585
4	acacetin-7-O-β -D-glucoside	P03407
4	acacetin-7-O-β -D-glucoside	P30480
4	acacetin-7-O-β -D-glucoside	P69726
4	acacetin-7-O-β -D-glucoside	Q31610
4	acacetin-7-O-β -D-glucoside	P13746
4	acacetin-7-O-β -D-glucoside	P04601
4	acacetin-7-O-β -D-glucoside	P05534
4	acacetin-7-O-β -D-glucoside	P19544

4	acacetin-7-O-β-D-glucoside	P06126
4	acacetin-7-O-β-D-glucoside	P18465
4	acacetin-7-O-β-D-glucoside	P43629
4	acacetin-7-O-β-D-glucoside	P43357
4	acacetin-7-O-β-D-glucoside	A0A578
4	acacetin-7-O-β-D-glucoside	P30443
4	acacetin-7-O-β-D-glucoside	Q8WZ42
4	acacetin-7-O-β-D-glucoside	P29016
4	acacetin-7-O-β-D-glucoside	P29017
4	acacetin-7-O-β-D-glucoside	P18139
4	acacetin-7-O-β-D-glucoside	Q95460
4	acacetin-7-O-β-D-glucoside	P03989
4	acacetin-7-O-β-D-glucoside	P32241
4	acacetin-7-O-β-D-glucoside	P01889
4	acacetin-7-O-β-D-glucoside	P12478
4	acacetin-7-O-β-D-glucoside	Q8NC51
4	acacetin-7-O-β-D-glucoside	Q96L21
4	acacetin-7-O-β-D-glucoside	P62701
4	acacetin-7-O-β-D-glucoside	P05388
4	acacetin-7-O-β-D-glucoside	P18077
4	acacetin-7-O-β-D-glucoside	P18124
4	acacetin-7-O-β-D-glucoside	P18621
4	acacetin-7-O-β-D-glucoside	P26373
4	acacetin-7-O-β-D-glucoside	P27635
4	acacetin-7-O-β-D-glucoside	P30050
4	acacetin-7-O-β-D-glucoside	P58908
4	acacetin-7-O-β-D-glucoside	P49137
4	acacetin-7-O-β-D-glucoside	Q16539
4	acacetin-7-O-β-D-glucoside	Q99538
4	acacetin-7-O-β-D-glucoside	P43235
4	acacetin-7-O-β-D-glucoside	P01023
4	acacetin-7-O-β-D-glucoside	P13699
4	acacetin-7-O-β-D-glucoside	P41159
4	acacetin-7-O-β-D-glucoside	O44249
4	acacetin-7-O-β-D-glucoside	Q25519

4	acacetin-7-O-β -D-glucoside	P14902
4	acacetin-7-O-β -D-glucoside	P49862
4	acacetin-7-O-β -D-glucoside	Q06124
4	acacetin-7-O-β -D-glucoside	Q15208
4	acacetin-7-O-β -D-glucoside	Q5E997
4	acacetin-7-O-β -D-glucoside	P02638
5	apigenin-8-C-(2-O-feruloyl)-β -D-glucoside	Q06277
5	apigenin-8-C-(2-O-feruloyl)-β -D-glucoside	Q9Z297
5	apigenin-8-C-(2-O-feruloyl)-β -D-glucoside	Q15121
5	apigenin-8-C-(2-O-feruloyl)-β -D-glucoside	Q9UQB8
5	apigenin-8-C-(2-O-feruloyl)-β -D-glucoside	Q7L576
5	apigenin-8-C-(2-O-feruloyl)-β -D-glucoside	Q8WUW1
5	apigenin-8-C-(2-O-feruloyl)-β -D-glucoside	Q92558
5	apigenin-8-C-(2-O-feruloyl)-β -D-glucoside	Q9NYB9
5	apigenin-8-C-(2-O-feruloyl)-β -D-glucoside	Q9Y2A7
5	apigenin-8-C-(2-O-feruloyl)-β -D-glucoside	P27361
5	apigenin-8-C-(2-O-feruloyl)-β -D-glucoside	Q96PY5
5	apigenin-8-C-(2-O-feruloyl)-β -D-glucoside	O95466
5	apigenin-8-C-(2-O-feruloyl)-β -D-glucoside	P40763
5	apigenin-8-C-(2-O-feruloyl)-β -D-glucoside	P28482
5	apigenin-8-C-(2-O-feruloyl)-β -D-glucoside	P23727
5	apigenin-8-C-(2-O-feruloyl)-β -D-glucoside	P63000
5	apigenin-8-C-(2-O-feruloyl)-β -D-glucoside	P60953
5	apigenin-8-C-(2-O-feruloyl)-β -D-glucoside	Q8TCU6
5	apigenin-8-C-(2-O-feruloyl)-β -D-glucoside	P27986
5	apigenin-8-C-(2-O-feruloyl)-β -D-glucoside	P42336
5	apigenin-8-C-(2-O-feruloyl)-β -D-glucoside	Q6YWF1
5	apigenin-8-C-(2-O-feruloyl)-β -D-glucoside	P05386
5	apigenin-8-C-(2-O-feruloyl)-β -D-glucoside	P05387
5	apigenin-8-C-(2-O-feruloyl)-β -D-glucoside	P05388
5	apigenin-8-C-(2-O-feruloyl)-β -D-glucoside	P13639
5	apigenin-8-C-(2-O-feruloyl)-β -D-glucoside	P18077
5	apigenin-8-C-(2-O-feruloyl)-β -D-glucoside	P18124
5	apigenin-8-C-(2-O-feruloyl)-β -D-glucoside	P18621
5	apigenin-8-C-(2-O-feruloyl)-β -D-glucoside	P26373

5	apigenin-8-C-(2-O-feruloyl)-β-D-glucoside	P30050
5	apigenin-8-C-(2-O-feruloyl)-β-D-glucoside	P32969
5	apigenin-8-C-(2-O-feruloyl)-β-D-glucoside	P35268
5	apigenin-8-C-(2-O-feruloyl)-β-D-glucoside	P36578
5	apigenin-8-C-(2-O-feruloyl)-β-D-glucoside	P39023
5	apigenin-8-C-(2-O-feruloyl)-β-D-glucoside	P40429
5	apigenin-8-C-(2-O-feruloyl)-β-D-glucoside	P42766
5	apigenin-8-C-(2-O-feruloyl)-β-D-glucoside	P46776
5	apigenin-8-C-(2-O-feruloyl)-β-D-glucoside	P46777
5	apigenin-8-C-(2-O-feruloyl)-β-D-glucoside	P46778
5	apigenin-8-C-(2-O-feruloyl)-β-D-glucoside	P46779
5	apigenin-8-C-(2-O-feruloyl)-β-D-glucoside	P47914
5	apigenin-8-C-(2-O-feruloyl)-β-D-glucoside	P49207
5	apigenin-8-C-(2-O-feruloyl)-β-D-glucoside	P50914
5	apigenin-8-C-(2-O-feruloyl)-β-D-glucoside	P61254
5	apigenin-8-C-(2-O-feruloyl)-β-D-glucoside	P61313
5	apigenin-8-C-(2-O-feruloyl)-β-D-glucoside	P61353
5	apigenin-8-C-(2-O-feruloyl)-β-D-glucoside	P61513
5	apigenin-8-C-(2-O-feruloyl)-β-D-glucoside	P61927
5	apigenin-8-C-(2-O-feruloyl)-β-D-glucoside	P62424
5	apigenin-8-C-(2-O-feruloyl)-β-D-glucoside	P62750
5	apigenin-8-C-(2-O-feruloyl)-β-D-glucoside	P62829
5	apigenin-8-C-(2-O-feruloyl)-β-D-glucoside	P62888
5	apigenin-8-C-(2-O-feruloyl)-β-D-glucoside	P62891
5	apigenin-8-C-(2-O-feruloyl)-β-D-glucoside	P62899
5	apigenin-8-C-(2-O-feruloyl)-β-D-glucoside	P62906
5	apigenin-8-C-(2-O-feruloyl)-β-D-glucoside	P62910
5	apigenin-8-C-(2-O-feruloyl)-β-D-glucoside	P62913
5	apigenin-8-C-(2-O-feruloyl)-β-D-glucoside	P62917
5	apigenin-8-C-(2-O-feruloyl)-β-D-glucoside	P62987
5	apigenin-8-C-(2-O-feruloyl)-β-D-glucoside	P63173
5	apigenin-8-C-(2-O-feruloyl)-β-D-glucoside	P83881
5	apigenin-8-C-(2-O-feruloyl)-β-D-glucoside	Q02543
5	apigenin-8-C-(2-O-feruloyl)-β-D-glucoside	P27705
5	apigenin-8-C-(2-O-feruloyl)-β-D-glucoside	P19438

5	apigenin-8-C-(2-O-feruloyl)-β-D-glucoside	P02775
5	apigenin-8-C-(2-O-feruloyl)-β-D-glucoside	A2Q6L5
5	apigenin-8-C-(2-O-feruloyl)-β-D-glucoside	E9PMV2
5	apigenin-8-C-(2-O-feruloyl)-β-D-glucoside	O43612
5	apigenin-8-C-(2-O-feruloyl)-β-D-glucoside	Q13231
5	apigenin-8-C-(2-O-feruloyl)-β-D-glucoside	P03180
5	apigenin-8-C-(2-O-feruloyl)-β-D-glucoside	Q13651
5	apigenin-8-C-(2-O-feruloyl)-β-D-glucoside	O75594
5	apigenin-8-C-(2-O-feruloyl)-β-D-glucoside	Q96L21
5	apigenin-8-C-(2-O-feruloyl)-β-D-glucoside	P62701
5	apigenin-8-C-(2-O-feruloyl)-β-D-glucoside	P27635
5	apigenin-8-C-(2-O-feruloyl)-β-D-glucoside	P0C6H2
5	apigenin-8-C-(2-O-feruloyl)-β-D-glucoside	P01732
5	apigenin-8-C-(2-O-feruloyl)-β-D-glucoside	P13285
5	apigenin-8-C-(2-O-feruloyl)-β-D-glucoside	Q9YV12
5	apigenin-8-C-(2-O-feruloyl)-β-D-glucoside	P04439
5	apigenin-8-C-(2-O-feruloyl)-β-D-glucoside	P15812
5	apigenin-8-C-(2-O-feruloyl)-β-D-glucoside	Q3KST2
5	apigenin-8-C-(2-O-feruloyl)-β-D-glucoside	Q9QDK7
5	apigenin-8-C-(2-O-feruloyl)-β-D-glucoside	Q49PI7
5	apigenin-8-C-(2-O-feruloyl)-β-D-glucoside	Q692E0
5	apigenin-8-C-(2-O-feruloyl)-β-D-glucoside	E0YFW1
5	apigenin-8-C-(2-O-feruloyl)-β-D-glucoside	Q8NC51
6	cirsimarin	Q2EEY0
6	cirsimarin	Q5I2M5
6	cirsimarin	O00206
6	cirsimarin	Q9Y6Y9
6	cirsimarin	Q9EQU3
6	cirsimarin	Q9NR97
6	cirsimarin	P01665
6	cirsimarin	P01863
6	cirsimarin	P07288
6	cirsimarin	P9WI73
6	cirsimarin	A4L9V2
6	cirsimarin	Q4G1L3

6 cirsimarin	P43403
6 cirsimarin	P07200
6 cirsimarin	P01834
6 cirsimarin	P29459
6 cirsimarin	P9WPB3
6 cirsimarin	P9WPB5
6 cirsimarin	P9WFK7
6 cirsimarin	P10279
6 cirsimarin	P32455
6 cirsimarin	Q9NRX4
6 cirsimarin	P51452
6 cirsimarin	P04156
6 cirsimarin	P15311
6 cirsimarin	P01514
6 cirsimarin	P01515
6 cirsimarin	P05230
6 cirsimarin	P21579
6 cirsimarin	Q99584
6 cirsimarin	O00329
6 cirsimarin	O43927
6 cirsimarin	P01704
6 cirsimarin	P16298
6 cirsimarin	Q13569
6 cirsimarin	P42224
6 cirsimarin	O75928
6 cirsimarin	Q9UER7
6 cirsimarin	P49792
6 cirsimarin	Q92793
6 cirsimarin	P63279
6 cirsimarin	Q9UBE0
6 cirsimarin	Q9UBT2
6 cirsimarin	P63165
6 cirsimarin	P01574
6 cirsimarin	P01563
6 cirsimarin	P02681

6	cirsimarin	P53634
6	cirsimarin	P09914
6	cirsimarin	P03527
6	cirsimarin	P04610
6	cirsimarin	P04613
6	cirsimarin	P18378
6	cirsimarin	P07939
6	cirsimarin	P0CK32
6	cirsimarin	P11077
6	cirsimarin	P11079
6	cirsimarin	P11314
6	cirsimarin	Q9WAB2
6	cirsimarin	P11207
6	cirsimarin	P62877
6	cirsimarin	Q13619
6	cirsimarin	Q16531
6	cirsimarin	P33064
6	cirsimarin	P0C569
6	cirsimarin	P07239
6	cirsimarin	Q1HVF7
6	cirsimarin	C5MK56
6	cirsimarin	P03211
6	cirsimarin	X2G898
6	cirsimarin	P30685
6	cirsimarin	Q9YRL3
6	cirsimarin	Q9YRL8
6	cirsimarin	P11305
6	cirsimarin	P16082
6	cirsimarin	P01887
6	cirsimarin	P69732
6	cirsimarin	Q860N6
6	cirsimarin	P35961
6	cirsimarin	O14788
6	cirsimarin	O35305
6	cirsimarin	P55899

6	cirsimarin	P61769
6	cirsimarin	P01730
6	cirsimarin	P20872
6	cirsimarin	P02794
6	cirsimarin	P10145
6	cirsimarin	P04141
6	cirsimarin	P24394
6	cirsimarin	P0CY27
6	cirsimarin	P03404
6	cirsimarin	P05112
6	cirsimarin	Q9EPW9
6	cirsimarin	Q9Y2C9
6	cirsimarin	P68638
6	cirsimarin	Q89859
6	cirsimarin	Q20NS3
6	cirsimarin	Q05315
6	cirsimarin	P02671
7	cirsimaritin	O60563
7	cirsimaritin	P04608
7	cirsimaritin	P50750
7	cirsimaritin	P03126
7	cirsimaritin	P04637
7	cirsimaritin	Q05086
7	cirsimaritin	P35260
7	cirsimaritin	P09601
7	cirsimaritin	P05000
7	cirsimaritin	P17181
7	cirsimaritin	P48551
7	cirsimaritin	P01562
7	cirsimaritin	Q5ZMD1
7	cirsimaritin	P01994
7	cirsimaritin	P09207
7	cirsimaritin	A5HUM9
7	cirsimaritin	Q1L1H6
7	cirsimaritin	Q9YKD7

7 cirsimaritin	Q5ZJG4
7 cirsimaritin	A0ZXM3
7 cirsimaritin	Q9PSX7
7 cirsimaritin	Q95601
7 cirsimaritin	O46789
7 cirsimaritin	Q9DG07
7 cirsimaritin	A1L565
7 cirsimaritin	O46790
7 cirsimaritin	Q6J4Y8
7 cirsimaritin	P68390
7 cirsimaritin	P08311
7 cirsimaritin	P03973
7 cirsimaritin	P04054
7 cirsimaritin	Q8IZI9
7 cirsimaritin	Q8IU54
7 cirsimaritin	Q8IU57
7 cirsimaritin	A0A0H3K0M1
7 cirsimaritin	P08246
7 cirsimaritin	P00592
7 cirsimaritin	P81534
7 cirsimaritin	P04083
7 cirsimaritin	P19619
7 cirsimaritin	P13987
7 cirsimaritin	Q9LCB8
7 cirsimaritin	O15455
7 cirsimaritin	O60486
7 cirsimaritin	O75326
7 cirsimaritin	P33672
7 cirsimaritin	Q2TBP0
7 cirsimaritin	Q2TBX6
7 cirsimaritin	Q2YDE4
7 cirsimaritin	Q32KL2
7 cirsimaritin	Q3MHN0
7 cirsimaritin	Q3T0X5
7 cirsimaritin	Q3T0Y5

7 cirsimaritin	Q3T108
7 cirsimaritin	Q3ZBG0
7 cirsimaritin	Q3ZCK9
7 cirsimaritin	Q58DU5
7 cirsimaritin	Q5E987
7 cirsimaritin	Q5E9K0
7 cirsimaritin	P07203
7 cirsimaritin	P01912
7 cirsimaritin	P35833
7 cirsimaritin	P35834
7 cirsimaritin	O00175
7 cirsimaritin	P04228
7 cirsimaritin	Q05329
7 cirsimaritin	P80075
7 cirsimaritin	P13725
7 cirsimaritin	Q1HVF7
7 cirsimaritin	C5MK56
7 cirsimaritin	P03211
7 cirsimaritin	X2G898
7 cirsimaritin	P30685
7 cirsimaritin	Q9YRL3
7 cirsimaritin	Q9YRL8
7 cirsimaritin	P0DOY3
7 cirsimaritin	Q9WUD1
7 cirsimaritin	Q13404
7 cirsimaritin	P52564
7 cirsimaritin	Q8VSD5
7 cirsimaritin	O76064
7 cirsimaritin	Q15819
7 cirsimaritin	P61088
7 cirsimaritin	P27635
7 cirsimaritin	P22090
7 cirsimaritin	P61086
7 cirsimaritin	Q8NC51
7 cirsimaritin	Q96L21

7	cirsimaritin	A0A158RFT3
7	cirsimaritin	A0A158RFT4
7	cirsimaritin	A0A158RFT5
7	cirsimaritin	Q5YD59
7	cirsimaritin	P01031
7	cirsimaritin	Q5QGG3
7	cirsimaritin	Q5QGH9
7	cirsimaritin	Q89490
7	cirsimaritin	Q9GJ77
7	cirsimaritin	Q9HBE4
7	cirsimaritin	Q9HBE5
7	cirsimaritin	O00300
7	cirsimaritin	P05113
7	cirsimaritin	Q01344
7	cirsimaritin	P03206
7	cirsimaritin	I2FL84
7	cirsimaritin	I2G9G1
7	cirsimaritin	P01552
7	cirsimaritin	P17213
8	esculetin	O15263
8	esculetin	Q9Y258
8	esculetin	P55773
8	esculetin	P03198
8	esculetin	Q8NKX2
8	esculetin	Q29983
8	esculetin	Q98823
8	esculetin	P42081
8	esculetin	P33681
8	esculetin	P25024
8	esculetin	P01910
8	esculetin	P02789
8	esculetin	P06343
8	esculetin	P08700
8	esculetin	P26718
8	esculetin	Q9BZM4

8	esculetin	P03205
8	esculetin	P00021
8	esculetin	P01904
8	esculetin	P04230
8	esculetin	Q9Y6Q6
8	esculetin	P01835
8	esculetin	P20759
8	esculetin	P22301
8	esculetin	P0A0L2
8	esculetin	P17150
8	esculetin	P02778
8	esculetin	P05121
8	esculetin	Q02223
8	esculetin	P12544
8	esculetin	P08887
8	esculetin	P40223
8	esculetin	P02777
8	esculetin	P40189
8	esculetin	P0A0L3
8	esculetin	P06239
8	esculetin	P19875
8	esculetin	P04626
8	esculetin	P11133
8	esculetin	P07353
8	esculetin	Q92838
8	esculetin	O14625
8	esculetin	P09341
8	esculetin	P12495
8	esculetin	P27705
8	esculetin	P19438
8	esculetin	P02775
8	esculetin	A2Q6L5
8	esculetin	E9PMV2
8	esculetin	O43612
8	esculetin	Q13231

8	esculetin	P03180
8	esculetin	Q13651
8	esculetin	O75594
8	esculetin	Q9GZX6
8	esculetin	P02686
8	esculetin	Q30154
8	esculetin	Q76RJ0
8	esculetin	P13727
8	esculetin	P25774
8	esculetin	O76096
8	esculetin	P09919
8	esculetin	Q99062
8	esculetin	Q15080
8	esculetin	P01589
8	esculetin	O10647
8	esculetin	P04664
8	esculetin	Q52T95
8	esculetin	O15117
8	esculetin	P12018
8	esculetin	P15814
8	esculetin	Q16663
8	esculetin	O95715
8	esculetin	P23510
8	esculetin	P43489
8	esculetin	A2NTY6
8	esculetin	P01738
8	esculetin	Q8NC51
8	esculetin	Q96L21
8	esculetin	P62701
8	esculetin	P05388
8	esculetin	P18077
8	esculetin	P18124
8	esculetin	P18621
8	esculetin	P26373
8	esculetin	P27635

8	esculetin	P30050
9	ISCARISIDE b	A8CDU0
9	ISCARISIDE b	P0A0L5
9	ISCARISIDE b	P18510
9	ISCARISIDE b	P78556
9	ISCARISIDE b	P01583
9	ISCARISIDE b	Q9Y4X3
9	ISCARISIDE b	O00585
9	ISCARISIDE b	P42830
9	ISCARISIDE b	Q99731
9	ISCARISIDE b	P51671
9	ISCARISIDE b	Q49DS8
9	ISCARISIDE b	Q6N095
9	ISCARISIDE b	Q993A8
9	ISCARISIDE b	P19551
9	ISCARISIDE b	Q6N030
9	ISCARISIDE b	Q6P5S8
9	ISCARISIDE b	Q8Q7Z9
9	ISCARISIDE b	O41925
9	ISCARISIDE b	Q48898
9	ISCARISIDE b	Q91LS8
9	ISCARISIDE b	P05106
9	ISCARISIDE b	P15018
9	ISCARISIDE b	P42703
9	ISCARISIDE b	Q16627
9	ISCARISIDE b	A4F255
9	ISCARISIDE b	P01857
9	ISCARISIDE b	Q99616
9	ISCARISIDE b	P02458
9	ISCARISIDE b	P01911
9	ISCARISIDE b	P16757
9	ISCARISIDE b	Q29980
9	ISCARISIDE b	P41273
9	ISCARISIDE b	Q30291
9	ISCARISIDE b	Q4MYJ2

9 ISCARISIDE b	P0A0M0
9 ISCARISIDE b	Q03909
9 ISCARISIDE b	P22413
9 ISCARISIDE b	P20333
9 ISCARISIDE b	P01675
9 ISCARISIDE b	P01837
9 ISCARISIDE b	P68104
9 ISCARISIDE b	Q05639
9 ISCARISIDE b	P13232
9 ISCARISIDE b	P16871
9 ISCARISIDE b	P01897
9 ISCARISIDE b	Q02218
9 ISCARISIDE b	P12273
9 ISCARISIDE b	P25311
9 ISCARISIDE b	Q13158
9 ISCARISIDE b	P0C8E7
9 ISCARISIDE b	Q65XZ7
9 ISCARISIDE b	Q9DHW0
9 ISCARISIDE b	P04578
9 ISCARISIDE b	P04070
9 ISCARISIDE b	P19505
9 ISCARISIDE b	Q30597
9 ISCARISIDE b	Q9WH73
9 ISCARISIDE b	Q16655
9 ISCARISIDE b	P05452
9 ISCARISIDE b	Q14627
9 ISCARISIDE b	Q1PHM6
9 ISCARISIDE b	O95150
9 ISCARISIDE b	P01902
9 ISCARISIDE b	P27930
9 ISCARISIDE b	Q7TDW8
9 ISCARISIDE b	P16410
9 ISCARISIDE b	P21611
9 ISCARISIDE b	Q9BCW3
9 ISCARISIDE b	A0A0A8WFP6

9	ISCARISIDE b	A0A173ADG5
9	ISCARISIDE b	P41355
9	ISCARISIDE b	Q95477
9	ISCARISIDE b	P29965
9	ISCARISIDE b	Q9Q0U7
9	ISCARISIDE b	C4PK56
9	ISCARISIDE b	O19244
9	ISCARISIDE b	Q07717
9	ISCARISIDE b	Q5QGG3
9	ISCARISIDE b	Q5QGH9
9	ISCARISIDE b	Q89490
9	ISCARISIDE b	Q9GJ77
9	ISCARISIDE b	Q9HBE4
9	ISCARISIDE b	Q9HBE5
9	ISCARISIDE b	O00300
9	ISCARISIDE b	P05113
9	ISCARISIDE b	Q01344
9	ISCARISIDE b	P03206
9	ISCARISIDE b	I2FL84
9	ISCARISIDE b	I2G9G1
9	ISCARISIDE b	P01552
9	ISCARISIDE b	Q58DU5
9	ISCARISIDE b	Q5E987
9	ISCARISIDE b	Q5E9K0
9	ISCARISIDE b	P07203
9	ISCARISIDE b	P01912
9	ISCARISIDE b	P35833
9	ISCARISIDE b	P35834
9	ISCARISIDE b	O00175
9	ISCARISIDE b	P04228
9	ISCARISIDE b	Q05329
9	ISCARISIDE b	P80075
9	ISCARISIDE b	P13725
9	ISCARISIDE b	Q1HVF7
10	isoquercetin	P32969

10 isoquercetin	P35268
10 isoquercetin	P36578
10 isoquercetin	P39023
10 isoquercetin	P40429
10 isoquercetin	P42766
10 isoquercetin	P46776
10 isoquercetin	P46777
10 isoquercetin	P46778
10 isoquercetin	P46779
10 isoquercetin	P47914
10 isoquercetin	P49207
10 isoquercetin	P50914
10 isoquercetin	P61254
10 isoquercetin	P61313
10 isoquercetin	P61353
10 isoquercetin	P61513
10 isoquercetin	P61927
10 isoquercetin	P62424
10 isoquercetin	P62750
10 isoquercetin	P62829
10 isoquercetin	P62888
10 isoquercetin	P62891
10 isoquercetin	P62899
10 isoquercetin	P62906
10 isoquercetin	P62910
10 isoquercetin	P62913
10 isoquercetin	P62917
10 isoquercetin	P62945
10 isoquercetin	P62987
10 isoquercetin	P63173
10 isoquercetin	P83731
10 isoquercetin	P83881
10 isoquercetin	P84098
10 isoquercetin	Q02543
10 isoquercetin	Q02878

10	isoquercetin	Q07020
10	isoquercetin	Q9Y3U8
10	isoquercetin	P08708
10	isoquercetin	P08865
10	isoquercetin	P15880
10	isoquercetin	P22090
10	isoquercetin	P23396
10	isoquercetin	P25398
10	isoquercetin	P39019
10	isoquercetin	P42677
10	isoquercetin	P46781
10	isoquercetin	P46782
10	isoquercetin	P46783
10	isoquercetin	P60866
10	isoquercetin	P61247
10	isoquercetin	P62081
10	isoquercetin	P62241
10	isoquercetin	P62244
10	isoquercetin	P62249
10	isoquercetin	P62263
10	isoquercetin	P62266
10	isoquercetin	P62269
10	isoquercetin	P62273
10	isoquercetin	P62277
10	isoquercetin	P62280
10	isoquercetin	P62753
10	isoquercetin	P62841
10	isoquercetin	P62847
10	isoquercetin	P62851
10	isoquercetin	P62854
10	isoquercetin	P62857
10	isoquercetin	P62861
10	isoquercetin	P62979
10	isoquercetin	P63220
10	isoquercetin	P63244

10	isoquercetin	Q8NC51
10	isoquercetin	Q96L21
10	isoquercetin	P62701
10	isoquercetin	P05388
10	isoquercetin	P18077
10	isoquercetin	P18124
10	isoquercetin	P18621
10	isoquercetin	P26373
10	isoquercetin	P27635
10	isoquercetin	P30050
11	orientin	P05112
11	orientin	A4L9V2
11	orientin	Q4G1L3
11	orientin	P05231
11	orientin	P43405
11	orientin	P43403
11	orientin	P09601
11	orientin	Q8IU54
11	orientin	Q8IU57
11	orientin	P01834
11	orientin	P01857
11	orientin	P29459
11	orientin	P29460
11	orientin	Q9NPF7
11	orientin	P01514
11	orientin	P01515
11	orientin	P05230
11	orientin	P21579
11	orientin	P01583
11	orientin	Q15109
11	orientin	Q99584
11	orientin	O00329
11	orientin	P23727
11	orientin	P01574
11	orientin	P05000

11 orientin	P17181
11 orientin	P48551
11 orientin	P01562
11 orientin	P01563
11 orientin	P49768
11 orientin	Q92542
11 orientin	Q96BI3
11 orientin	Q9NZ42
11 orientin	P0C8E7
11 orientin	P10147
11 orientin	Q2F862
11 orientin	Q9ULZ3
11 orientin	Q8WXC3
11 orientin	O75594
11 orientin	P15260
11 orientin	P01589
11 orientin	P30123
11 orientin	P01579
11 orientin	Q66793
11 orientin	P14784
11 orientin	P31785
11 orientin	P60568
11 orientin	A3FBE6
11 orientin	P00813
11 orientin	O95711
11 orientin	Q99467
11 orientin	P07200
11 orientin	Q06265
11 orientin	Q13868
11 orientin	Q15024
11 orientin	Q5RKV6
11 orientin	Q96B26
11 orientin	Q9NPD3
11 orientin	Q9NQT4
11 orientin	Q9NQT5

11	orientin	Q9Y3B2
11	orientin	P52701
11	orientin	P20585
11	orientin	P43246
11	orientin	P04083
11	orientin	P19619
11	orientin	P52566
11	orientin	P15153
11	orientin	P16885
11	orientin	Q15833
11	orientin	P12429
11	orientin	P02775
11	orientin	P10809
11	orientin	P61604
11	orientin	Q99731
11	orientin	P24394
11	orientin	P35225
11	orientin	P78552
11	orientin	P58908
11	orientin	P05386
11	orientin	P05387
11	orientin	P13639
11	orientin	Q8NC51
11	orientin	Q96L21
11	orientin	P62701
11	orientin	P05388
11	orientin	P18077
11	orientin	P18124
11	orientin	P18621
11	orientin	P26373
11	orientin	P27635
11	orientin	P30050
12	pectolinarigenin	Q40960
12	pectolinarigenin	P85524
12	pectolinarigenin	P23526

12 pectolinarigenin	P08575
12 pectolinarigenin	P11275
12 pectolinarigenin	P26645
12 pectolinarigenin	P62937
12 pectolinarigenin	P40136
12 pectolinarigenin	Q9UIK4
12 pectolinarigenin	Q810C8
12 pectolinarigenin	Q13813
12 pectolinarigenin	Q15746
12 pectolinarigenin	P23634
12 pectolinarigenin	Q63450
12 pectolinarigenin	P14151
12 pectolinarigenin	Q9H1R3
12 pectolinarigenin	Q00195
12 pectolinarigenin	P37840
12 pectolinarigenin	P29474
12 pectolinarigenin	Q9UKL4
12 pectolinarigenin	Q13698
12 pectolinarigenin	Q13557
12 pectolinarigenin	P53355
12 pectolinarigenin	Q05586
12 pectolinarigenin	Q05152
12 pectolinarigenin	Q07652
12 pectolinarigenin	P27884
12 pectolinarigenin	P25445
12 pectolinarigenin	P08138
12 pectolinarigenin	P35228
12 pectolinarigenin	P62157
12 pectolinarigenin	Q6J8I9
12 pectolinarigenin	P24588
12 pectolinarigenin	P0DP24
12 pectolinarigenin	P11799
12 pectolinarigenin	P42212
12 pectolinarigenin	Q13936
12 pectolinarigenin	O35433

12	pectolinarigenin	P53779
12	pectolinarigenin	O00159
12	pectolinarigenin	O36972
12	pectolinarigenin	P63098
12	pectolinarigenin	P56696
12	pectolinarigenin	Q92913
12	pectolinarigenin	Q99250
12	pectolinarigenin	P61328
12	pectolinarigenin	Q05096
12	pectolinarigenin	Q9H095
12	pectolinarigenin	Q14524
12	pectolinarigenin	Q08209
12	pectolinarigenin	P23677
12	pectolinarigenin	P0DP23
12	pectolinarigenin	P51787
12	pectolinarigenin	O00571
12	pectolinarigenin	P68466
12	pectolinarigenin	P19508
12	pectolinarigenin	Q9Y3Z3
12	pectolinarigenin	Q9Y4B6
12	pectolinarigenin	P68598
12	pectolinarigenin	P11116
12	pectolinarigenin	P09382
12	pectolinarigenin	Q13651
12	pectolinarigenin	P22301
12	pectolinarigenin	Q8NC51
12	pectolinarigenin	Q96L21
12	pectolinarigenin	P62701
12	pectolinarigenin	P05388
12	pectolinarigenin	P18077
12	pectolinarigenin	P18124
12	pectolinarigenin	P18621
12	pectolinarigenin	P26373
12	pectolinarigenin	P27635
12	pectolinarigenin	P30050

12	pectolinarigenin	P27705
12	pectolinarigenin	P19438
12	pectolinarigenin	P02775
12	pectolinarigenin	A2Q6L5
12	pectolinarigenin	E9PMV2
12	pectolinarigenin	O43612
12	pectolinarigenin	Q13231
12	pectolinarigenin	P03180
12	pectolinarigenin	O75594
13	pinoresinol-β -D-glucopyranoside	P01032
13	pinoresinol-β -D-glucopyranoside	P01876
13	pinoresinol-β -D-glucopyranoside	P20023
13	pinoresinol-β -D-glucopyranoside	P17927
13	pinoresinol-β -D-glucopyranoside	P07357
13	pinoresinol-β -D-glucopyranoside	P07360
13	pinoresinol-β -D-glucopyranoside	P05156
13	pinoresinol-β -D-glucopyranoside	P02748
13	pinoresinol-β -D-glucopyranoside	P00813
13	pinoresinol-β -D-glucopyranoside	P13489
13	pinoresinol-β -D-glucopyranoside	P24627
13	pinoresinol-β -D-glucopyranoside	O77698
13	pinoresinol-β -D-glucopyranoside	Q9TUM0
13	pinoresinol-β -D-glucopyranoside	Q29477
13	pinoresinol-β -D-glucopyranoside	P84156
13	pinoresinol-β -D-glucopyranoside	P02788
13	pinoresinol-β -D-glucopyranoside	Q8T0W8
13	pinoresinol-β -D-glucopyranoside	O77811
13	pinoresinol-β -D-glucopyranoside	P03950
13	pinoresinol-β -D-glucopyranoside	O95711
13	pinoresinol-β -D-glucopyranoside	Q99467
13	pinoresinol-β -D-glucopyranoside	Q06265
13	pinoresinol-β -D-glucopyranoside	Q13868
13	pinoresinol-β -D-glucopyranoside	Q15024
13	pinoresinol-β -D-glucopyranoside	Q5RKV6
13	pinoresinol-β -D-glucopyranoside	Q96B26

13	pinoresinol-β -D-glucopyranoside	Q9NPD3
13	pinoresinol-β -D-glucopyranoside	Q9NQT4
13	pinoresinol-β -D-glucopyranoside	Q9NQT5
13	pinoresinol-β -D-glucopyranoside	Q9Y3B2
13	pinoresinol-β -D-glucopyranoside	P52701
13	pinoresinol-β -D-glucopyranoside	P20585
13	pinoresinol-β -D-glucopyranoside	P43246
13	pinoresinol-β -D-glucopyranoside	P14174
13	pinoresinol-β -D-glucopyranoside	P20160
13	pinoresinol-β -D-glucopyranoside	P47992
13	pinoresinol-β -D-glucopyranoside	P10809
13	pinoresinol-β -D-glucopyranoside	P61604
13	pinoresinol-β -D-glucopyranoside	Q6TQF5
13	pinoresinol-β -D-glucopyranoside	P03502
13	pinoresinol-β -D-glucopyranoside	P05161
13	pinoresinol-β -D-glucopyranoside	P20591
13	pinoresinol-β -D-glucopyranoside	Q05193
13	pinoresinol-β -D-glucopyranoside	P34897
13	pinoresinol-β -D-glucopyranoside	Q03026
13	pinoresinol-β -D-glucopyranoside	Q03023
13	pinoresinol-β -D-glucopyranoside	P02652
13	pinoresinol-β -D-glucopyranoside	P02647
13	pinoresinol-β -D-glucopyranoside	O43521
13	pinoresinol-β -D-glucopyranoside	P16778
13	pinoresinol-β -D-glucopyranoside	Q07812
13	pinoresinol-β -D-glucopyranoside	P30491
13	pinoresinol-β -D-glucopyranoside	A0A075B6T6
13	pinoresinol-β -D-glucopyranoside	P36897
13	pinoresinol-β -D-glucopyranoside	A0JD25
13	pinoresinol-β -D-glucopyranoside	Q31615
13	pinoresinol-β -D-glucopyranoside	P04858
13	pinoresinol-β -D-glucopyranoside	P02786
13	pinoresinol-β -D-glucopyranoside	Q30201
13	pinoresinol-β -D-glucopyranoside	P24740
13	pinoresinol-β -D-glucopyranoside	P04222

13	pinoresinol-β -D-glucopyranoside	P43627
13	pinoresinol-β -D-glucopyranoside	P52292
13	pinoresinol-β -D-glucopyranoside	P15260
13	pinoresinol-β -D-glucopyranoside	P04582
13	pinoresinol-β -D-glucopyranoside	P12901
13	pinoresinol-β -D-glucopyranoside	P04588
13	pinoresinol-β -D-glucopyranoside	P43358
13	pinoresinol-β -D-glucopyranoside	P09713
13	pinoresinol-β -D-glucopyranoside	P43626
13	pinoresinol-β -D-glucopyranoside	O60880
13	pinoresinol-β -D-glucopyranoside	Q13291
13	pinoresinol-β -D-glucopyranoside	P04857
13	pinoresinol-β -D-glucopyranoside	P30504
13	pinoresinol-β -D-glucopyranoside	P04602
13	pinoresinol-β -D-glucopyranoside	Q31206
13	pinoresinol-β -D-glucopyranoside	O11818
13	pinoresinol-β -D-glucopyranoside	Q9Y5X3
13	pinoresinol-β -D-glucopyranoside	Q9TQB0
13	pinoresinol-β -D-glucopyranoside	Q07352
13	pinoresinol-β -D-glucopyranoside	P12977
13	pinoresinol-β -D-glucopyranoside	P51965
13	pinoresinol-β -D-glucopyranoside	P06334
13	pinoresinol-β -D-glucopyranoside	Q9BCZ1
13	pinoresinol-β -D-glucopyranoside	Q860W6
13	pinoresinol-β -D-glucopyranoside	P14079
13	pinoresinol-β -D-glucopyranoside	P27705
13	pinoresinol-β -D-glucopyranoside	P19438
13	pinoresinol-β -D-glucopyranoside	P02775
13	pinoresinol-β -D-glucopyranoside	A2Q6L5
13	pinoresinol-β -D-glucopyranoside	E9PMV2
13	pinoresinol-β -D-glucopyranoside	O43612
13	pinoresinol-β -D-glucopyranoside	Q13231
13	pinoresinol-β -D-glucopyranoside	P03180
13	pinoresinol-β -D-glucopyranoside	Q13651
13	pinoresinol-β -D-glucopyranoside	O75594

13	pinoresinol-β -D-glucopyranoside	P48023
13	pinoresinol-β -D-glucopyranoside	P01374
13	pinoresinol-β -D-glucopyranoside	Q06643
13	pinoresinol-β -D-glucopyranoside	F6ZMI5
13	pinoresinol-β -D-glucopyranoside	O14763
13	pinoresinol-β -D-glucopyranoside	P50591
13	pinoresinol-β -D-glucopyranoside	P60568
14	progloberflowery acid	P81544
14	progloberflowery acid	P82818
14	progloberflowery acid	Q92844
14	progloberflowery acid	P52907
14	progloberflowery acid	P36941
14	progloberflowery acid	Q27084
14	progloberflowery acid	Q76BK2
14	progloberflowery acid	Q6RYA0
14	progloberflowery acid	P13198
14	progloberflowery acid	Q8T9R8
14	progloberflowery acid	P20459
14	progloberflowery acid	P81613
14	progloberflowery acid	Q13114
14	progloberflowery acid	P07947
14	progloberflowery acid	B3RFR8
14	progloberflowery acid	Q86LE5
14	progloberflowery acid	D8L127
14	progloberflowery acid	Q9I9P7
14	progloberflowery acid	P86471
14	progloberflowery acid	Q8N104
14	progloberflowery acid	P09038
14	progloberflowery acid	Q17NR1
14	progloberflowery acid	P80511
14	progloberflowery acid	Q9C035
14	progloberflowery acid	P51124
14	progloberflowery acid	P12318
14	progloberflowery acid	Q90890
14	progloberflowery acid	P21760

14	progloberflowery acid	P19525
14	progloberflowery acid	Q0PF16
14	progloberflowery acid	Q9NRW3
14	progloberflowery acid	G4V4F9
14	progloberflowery acid	P31151
14	progloberflowery acid	P02743
14	progloberflowery acid	O95497
14	progloberflowery acid	P0A6Y8
14	progloberflowery acid	P02751
14	progloberflowery acid	P0DJI8
14	progloberflowery acid	Q9NZH8
14	progloberflowery acid	Q13325
14	progloberflowery acid	P05067
14	progloberflowery acid	O94817
14	progloberflowery acid	Q9NT62
14	progloberflowery acid	P81172
14	progloberflowery acid	G9MAP5
14	progloberflowery acid	P00720
14	progloberflowery acid	Q7Z6L1
14	progloberflowery acid	Q9UM07
14	progloberflowery acid	P05109
14	progloberflowery acid	P06702
14	progloberflowery acid	P31941
14	progloberflowery acid	Q15109
14	progloberflowery acid	P80188
14	progloberflowery acid	Q9BT43
14	progloberflowery acid	Q9BUI4
14	progloberflowery acid	P04271
14	progloberflowery acid	Q15418
14	progloberflowery acid	Q676U5
14	progloberflowery acid	Q9H1Y0
14	progloberflowery acid	Q9GK12
14	progloberflowery acid	P80408
14	progloberflowery acid	P0DOY6
14	progloberflowery acid	P0DOY7

14	progloberflowery acid	P0DOY8
14	progloberflowery acid	P0DOY9
14	progloberflowery acid	P17291
14	progloberflowery acid	P35871
14	progloberflowery acid	P37362
14	progloberflowery acid	P60405
14	progloberflowery acid	P60488
14	progloberflowery acid	P60489
14	progloberflowery acid	P60490
14	progloberflowery acid	P60491
14	progloberflowery acid	P60492
14	progloberflowery acid	P60493
14	progloberflowery acid	P60494
14	progloberflowery acid	P80339
14	progloberflowery acid	P80340
14	progloberflowery acid	P80371
14	progloberflowery acid	Q8NC51
14	progloberflowery acid	Q96L21
14	progloberflowery acid	P62701
14	progloberflowery acid	P05388
14	progloberflowery acid	P18077
14	progloberflowery acid	P18124
14	progloberflowery acid	P18621
14	progloberflowery acid	P26373
14	progloberflowery acid	P27635
14	progloberflowery acid	P30050
15	protocatechuic acid	P0AEX9
15	protocatechuic acid	Q9NP55
15	protocatechuic acid	P12724
15	protocatechuic acid	Q6NXT2
15	protocatechuic acid	P84243
15	protocatechuic acid	Q71DI3
15	protocatechuic acid	P04908
15	protocatechuic acid	P06899
15	protocatechuic acid	P62805

15	protocatechuic acid	P68431
15	protocatechuic acid	P55211
15	protocatechuic acid	Q9NR28
15	protocatechuic acid	Q12933
15	protocatechuic acid	Q13077
15	protocatechuic acid	Q13489
15	protocatechuic acid	P98170
15	protocatechuic acid	Q13490
15	protocatechuic acid	P52566
15	protocatechuic acid	P15153
15	protocatechuic acid	P16885
15	protocatechuic acid	Q15833
15	protocatechuic acid	Q9TTF5
15	protocatechuic acid	P12530
15	protocatechuic acid	P58908
15	protocatechuic acid	P49137
15	protocatechuic acid	Q16539
15	protocatechuic acid	Q99538
15	protocatechuic acid	P43235
15	protocatechuic acid	P01023
15	protocatechuic acid	P13699
15	protocatechuic acid	P41159
15	protocatechuic acid	O44249
15	protocatechuic acid	Q25519
15	protocatechuic acid	P14902
15	protocatechuic acid	P49862
15	protocatechuic acid	Q06124
15	protocatechuic acid	Q15208
15	protocatechuic acid	Q5E997
15	protocatechuic acid	P02638
15	protocatechuic acid	P11116
15	protocatechuic acid	P09382
15	protocatechuic acid	Q96AZ6
15	protocatechuic acid	P09913
15	protocatechuic acid	Q05823

15	protocatechuic acid	O95155
15	protocatechuic acid	P0CG48
15	protocatechuic acid	Q99PZ6
15	protocatechuic acid	Q9UHD2
15	protocatechuic acid	P02281
15	protocatechuic acid	P06897
15	protocatechuic acid	P35226
15	protocatechuic acid	P62799
15	protocatechuic acid	P84233
15	protocatechuic acid	P61077
15	protocatechuic acid	Q86SE9
15	protocatechuic acid	Q99496
15	protocatechuic acid	Q86WV6
15	protocatechuic acid	A7SLZ2
15	protocatechuic acid	Q10588
15	protocatechuic acid	P06744
15	protocatechuic acid	P49768
15	protocatechuic acid	Q92542
15	protocatechuic acid	Q96BI3
15	protocatechuic acid	Q9NZ42
15	protocatechuic acid	P25942
15	protocatechuic acid	Q8NC51
15	protocatechuic acid	Q96L21
15	protocatechuic acid	P62701
15	protocatechuic acid	P05388
15	protocatechuic acid	P18077
15	protocatechuic acid	P18124
15	protocatechuic acid	P18621
15	protocatechuic acid	P26373
15	protocatechuic acid	P27635
15	protocatechuic acid	P30050
15	protocatechuic acid	P29016
15	protocatechuic acid	P29017
15	protocatechuic acid	P18139
15	protocatechuic acid	Q95460

15	protocatechuic acid	P03989
15	protocatechuic acid	P32241
15	protocatechuic acid	P01889
15	protocatechuic acid	P12478
15	protocatechuic acid	P0C6H2
15	protocatechuic acid	P01732
15	protocatechuic acid	P13285
15	protocatechuic acid	Q9YV12
15	protocatechuic acid	P04439
15	protocatechuic acid	P15812
15	protocatechuic acid	Q3KST2
15	protocatechuic acid	Q9QDK7
15	protocatechuic acid	Q49PI7
15	protocatechuic acid	Q692E0
15	protocatechuic acid	E0YFW1
16	quercetin	Q02878
16	quercetin	Q07020
16	quercetin	Q8NC51
16	quercetin	Q96L21
16	quercetin	Q9Y3U8
16	quercetin	P08708
16	quercetin	P08865
16	quercetin	P15880
16	quercetin	P23396
16	quercetin	P25398
16	quercetin	P39019
16	quercetin	P42677
16	quercetin	P46781
16	quercetin	P46782
16	quercetin	P46783
16	quercetin	P60866
16	quercetin	P61247
16	quercetin	P62081
16	quercetin	P62241
16	quercetin	P62244

16	quercetin	P62249
16	quercetin	P62263
16	quercetin	P62266
16	quercetin	P62269
16	quercetin	P62273
16	quercetin	P62277
16	quercetin	P62280
16	quercetin	P62701
16	quercetin	P62753
16	quercetin	P62841
16	quercetin	P62847
16	quercetin	P62851
16	quercetin	P62854
16	quercetin	P62857
16	quercetin	P62861
16	quercetin	P62945
16	quercetin	P62979
16	quercetin	P63220
16	quercetin	P63244
16	quercetin	P83731
16	quercetin	P84098
16	quercetin	Q92583
16	quercetin	P13500
16	quercetin	P55008
16	quercetin	P00966
16	quercetin	P04406
16	quercetin	P13501
16	quercetin	P01308
16	quercetin	Q9UQM7
16	quercetin	Q14258
16	quercetin	Q8WXC3
16	quercetin	Q7SIC1
16	quercetin	Q9NZ08
16	quercetin	P07711
16	quercetin	P62837

16 quercetin	Q96FW1
16 quercetin	Q6P179
16 quercetin	P9WIA1
16 quercetin	P9WNK5
16 quercetin	P9WNK7
16 quercetin	A0A0H2VDN9
16 quercetin	Q9PRS8
16 quercetin	O60814
16 quercetin	Q9H1E1
16 quercetin	Q16695
16 quercetin	P0C0S5
16 quercetin	Q71UI9
16 quercetin	P49450
16 quercetin	P05388
16 quercetin	P18077
16 quercetin	P18124
16 quercetin	P18621
16 quercetin	P26373
16 quercetin	P27635
16 quercetin	P30050
17 trolline	P05107
17 trolline	P20702
17 trolline	A0A0B4J279
17 trolline	A0A592
17 trolline	A0A075B6N1
17 trolline	A0A087WT01
17 trolline	A0A0B4J2E0
17 trolline	P29350
17 trolline	Q13616
17 trolline	Q86VP6
17 trolline	O95628
17 trolline	P46527
17 trolline	P61024
17 trolline	Q13309
17 trolline	Q8OUW2

17 trolline	P12931
17 trolline	Q8WY64
17 trolline	P01111
17 trolline	Q96PU5
17 trolline	P21580
17 trolline	Q9H4M3
17 trolline	Q13191
17 trolline	P22681
17 trolline	O88846
17 trolline	Q96CA5
17 trolline	P43405
17 trolline	P63208
17 trolline	Q9R194
17 trolline	Q9UKT7
17 trolline	Q9XVR6
17 trolline	O75832
17 trolline	P51668
17 trolline	Q9CZW6
17 trolline	Q9H0F5
17 trolline	P25685
17 trolline	P17612
17 trolline	P61925
17 trolline	Q5X159
17 trolline	O14818
17 trolline	P20618
17 trolline	P25786
17 trolline	P25787
17 trolline	P25789
17 trolline	P28066
17 trolline	P28070
17 trolline	P28072
17 trolline	P28074
17 trolline	P49720
17 trolline	P49721
17 trolline	P60900

17 trolline	Q99436
17 trolline	Q6ZSG1
17 trolline	Q96BH1
17 trolline	P25788
17 trolline	P0CG47
17 trolline	Q96EP0
17 trolline	P01112
17 trolline	Q9ULZ3
17 trolline	Q9BXR5
17 trolline	Q15399
17 trolline	O60603
17 trolline	P06179
17 trolline	Q9QUN7
17 trolline	O60602
17 trolline	P30690
17 trolline	P27705
17 trolline	P19438
17 trolline	P02775
17 trolline	A2Q6L5
17 trolline	E9PMV2
17 trolline	O43612
17 trolline	Q13231
17 trolline	P03180
17 trolline	Q13651
17 trolline	O75594
17 trolline	P01903
17 trolline	P13760
17 trolline	O19707
17 trolline	P18573
17 trolline	Q30069
17 trolline	Q7Z434
17 trolline	Q9Y4K3
17 trolline	O75888
17 trolline	Q9Y275
17 trolline	A3FBE6

17	trolline	K4MU32
17	trolline	P12482
17	trolline	Q6V7J5
17	trolline	Q13822
17	trolline	P10147
17	trolline	P80098
17	trolline	Q2F862
18	trollioside	P11305
18	trollioside	P16082
18	trollioside	P01887
18	trollioside	P69732
18	trollioside	Q860N6
18	trollioside	P35961
18	trollioside	O14788
18	trollioside	O35305
18	trollioside	P55899
18	trollioside	P61769
18	trollioside	P01730
18	trollioside	P20872
18	trollioside	P02794
18	trollioside	P10145
18	trollioside	P04141
18	trollioside	P24394
18	trollioside	P35225
18	trollioside	P78552
18	trollioside	P01375
18	trollioside	P01584
18	trollioside	P42768
18	trollioside	Q07912
18	trollioside	P19803
18	trollioside	P35465
18	trollioside	P19878
18	trollioside	Q61036
18	trollioside	Q60610
18	trollioside	P74873

18 trollioside	P62993
18 trollioside	O52623
18 trollioside	Q51451
18 trollioside	P52565
18 trollioside	P53365
18 trollioside	Q64096
18 trollioside	Q9JK83
18 trollioside	Q9QX73
18 trollioside	P46108
18 trollioside	Q00722
18 trollioside	Q05608
18 trollioside	Q07960
18 trollioside	O75962
18 trollioside	Q9NQU5
18 trollioside	Q16512
18 trollioside	P27870
18 trollioside	O49003
18 trollioside	Q9BZ29
18 trollioside	Q9BUB5
18 trollioside	Q92608
18 trollioside	P15498
18 trollioside	Q9R8E4
18 trollioside	Q8BRU4
18 trollioside	P60709
18 trollioside	Q08043
18 trollioside	Q15811
18 trollioside	P70206
18 trollioside	O43157
18 trollioside	Q8C147
18 trollioside	O30916
18 trollioside	P36507
18 trollioside	P27705
18 trollioside	P19438
18 trollioside	P02775
18 trollioside	A2Q6L5

18 trollioside	E9PMV2
18 trollioside	O43612
18 trollioside	Q13231
18 trollioside	P03180
18 trollioside	Q13651
18 trollioside	O75594
18 trollioside	Q1HVF7
18 trollioside	C5MK56
18 trollioside	P03211
18 trollioside	X2G898
18 trollioside	P30685
18 trollioside	Q9YRL3
18 trollioside	Q9YRL8
18 trollioside	Q98Y46
18 trollioside	Q8URG0
18 trollioside	Q70A61
18 trollioside	O11822
18 trollioside	P26590
18 trollioside	P27797
18 trollioside	P16104
18 trollioside	Q8N423
18 trollioside	P16780
18 trollioside	P01899
18 trollioside	O19626
18 trollioside	P33260
18 trollioside	Q70AA1
19 veratric acid	P14483
19 veratric acid	P04440
19 veratric acid	P20036
19 veratric acid	Q0ED31
19 veratric acid	Q16552
19 veratric acid	P20871
19 veratric acid	Q73372
19 veratric acid	P13236
19 veratric acid	P14735

19 veratric acid	P02776
19 veratric acid	P15509
19 veratric acid	O43557
19 veratric acid	Q92956
19 veratric acid	Q29599
19 veratric acid	P61073
19 veratric acid	Q98157
19 veratric acid	Q9Y6K5
19 veratric acid	P48061
19 veratric acid	Q9UNN8
19 veratric acid	Q96RJ3
19 veratric acid	A0A0B4J271
19 veratric acid	A0A0K0K1A5
19 veratric acid	A0A5B9
19 veratric acid	P01848
19 veratric acid	P15813
19 veratric acid	O73909
19 veratric acid	P04233
19 veratric acid	P04229
19 veratric acid	Q15646
19 veratric acid	P69332
19 veratric acid	P78423
19 veratric acid	P01903
19 veratric acid	P13760
19 veratric acid	O19707
19 veratric acid	P18573
19 veratric acid	Q30069
19 veratric acid	Q7Z434
19 veratric acid	Q9Y4K3
19 veratric acid	O75888
19 veratric acid	Q9Y275
19 veratric acid	A3FBE6
19 veratric acid	K4MU32
19 veratric acid	P12482
19 veratric acid	Q6V7J5

19	veratric acid	Q13822
19	veratric acid	P10147
19	veratric acid	P80098
19	veratric acid	Q2F862
19	veratric acid	P22681
19	veratric acid	O88846
19	veratric acid	Q96CA5
19	veratric acid	P43405
19	veratric acid	P63208
19	veratric acid	Q9R194
19	veratric acid	Q9UKT7
19	veratric acid	Q9XVR6
19	veratric acid	O75832
19	veratric acid	P51668
19	veratric acid	Q9CZW6
19	veratric acid	Q9H0F5
19	veratric acid	P25685
19	veratric acid	P17612
19	veratric acid	P61925
19	veratric acid	Q5X159
19	veratric acid	O14818
19	veratric acid	O60602
19	veratric acid	P30690
19	veratric acid	P27705
19	veratric acid	P19438
19	veratric acid	P02775
19	veratric acid	A2Q6L5
19	veratric acid	E9PMV2
19	veratric acid	O43612
19	veratric acid	Q13231
19	veratric acid	P03180
19	veratric acid	Q13651
19	veratric acid	O75594
19	veratric acid	P60491
19	veratric acid	P60492

19	veratric acid	P60493
19	veratric acid	P60494
19	veratric acid	P80339
19	veratric acid	P80340
19	veratric acid	P80371
19	veratric acid	Q8NC51
19	veratric acid	Q96L21
19	veratric acid	P62701
19	veratric acid	P05388
19	veratric acid	P18077
19	veratric acid	P18124
19	veratric acid	P18621
19	veratric acid	P26373
19	veratric acid	P27635
19	veratric acid	P30050
20	vitexin	P03437
20	vitexin	L8E864
20	vitexin	Q94G94
20	vitexin	Q9NPH3
20	vitexin	Q6UXL0
20	vitexin	Q9NYY1
20	vitexin	Q9UHF4
20	vitexin	P60174
20	vitexin	P35585
20	vitexin	Q90VU7
20	vitexin	P14778
20	vitexin	P20718
20	vitexin	P28067
20	vitexin	K7N5N2
20	vitexin	P00491
20	vitexin	P29460
20	vitexin	Q9NPF7
20	vitexin	P14784
20	vitexin	P31785
20	vitexin	P40933

20 vitexin	Q13261
20 vitexin	L7MTK9
20 vitexin	L7MTL0
20 vitexin	P79483
20 vitexin	B8YAC7
20 vitexin	Q96F46
20 vitexin	P10720
20 vitexin	O43508
20 vitexin	P06340
20 vitexin	P13765
20 vitexin	P28068
20 vitexin	Q6ICR9
20 vitexin	P00973
20 vitexin	P40967
20 vitexin	Q75760
20 vitexin	P30466
20 vitexin	Q3KSS8
20 vitexin	P00749
20 vitexin	P04004
20 vitexin	Q03405
20 vitexin	Q28090
20 vitexin	P23946
20 vitexin	C1ITJ8
20 vitexin	P01888
20 vitexin	Q6P4G7
20 vitexin	Q30066
20 vitexin	Q67AJ6
20 vitexin	P00268
20 vitexin	P51681
20 vitexin	P16112
20 vitexin	P08670
20 vitexin	P55774
20 vitexin	Q6PJ56
20 vitexin	P11609
20 vitexin	O95407

20 vitexin	P48023
20 vitexin	P01374
20 vitexin	Q06643
20 vitexin	F6ZMI5
20 vitexin	O14763
20 vitexin	P50591
20 vitexin	P60568
20 vitexin	P32927
20 vitexin	P05231
20 vitexin	P22362
20 vitexin	P01892
20 vitexin	P01909
20 vitexin	P01920
20 vitexin	Q2YD82
20 vitexin	Q5Y7D3
20 vitexin	P01850
20 vitexin	P14434
20 vitexin	P51965
20 vitexin	P06334
20 vitexin	Q9BCZ1
20 vitexin	Q860W6
20 vitexin	P14079
20 vitexin	P27705
20 vitexin	P19438
20 vitexin	P02775
20 vitexin	A2Q6L5
20 vitexin	E9PMV2
20 vitexin	O43612
20 vitexin	Q13231
20 vitexin	P03180
20 vitexin	Q13651
20 vitexin	O75594
20 vitexin	P03211
20 vitexin	X2G898
20 vitexin	P30685

20	vitexin	Q9YRL3
20	vitexin	Q9YRL8
20	vitexin	Q13619
20	vitexin	Q16531

Table S3 The overlapping biological processes (BP) gene enrichment sub-network generated by the CytoNCA plugin of Cytoscape software

GOID	GOTerm	GOGroup	% Associated Genes	Nr. Genes	Associated Genes Found
GO:0043368	positive T cell selection	Group09	21.74	5.00	[ATP1A2, CAMK2D, HDAC4, SLC8A1, SLC9A1]
GO:0002360	T cell lineage commitment	Group09	15.15	5.00	[ATP1A2, CAMK2D, GRK2, SLC8A1, SLC9A1]
GO:0045581	negative regulation of T cell differentiation	Group09	30.77	4.00	[ATP1A2, CAMK2D, OSR1, OXSR1]
GO:0042504	tyrosine phosphorylation of Stat4 protein	Group09	28.57	4.00	[ATP1A2, CAMK2D, OSR1, OXSR1]
GO:0051135	positive regulation of NK T cell activation	Group09	23.53	4.00	[ATP1A2, CAMK2D, OSR1, OXSR1]
GO:0050730	regulation of peptidyl-tyrosine phosphorylation	Group09	6.59	6.00	[ATP1A2, CAMK2D, GRK2, HDAC4, SLC8A1, SLC9A1]
GO:0043369	CD4-positive or CD8-positive, alpha-beta T cell lineage commitment	Group09	6.38	6.00	[ATP1A2, CAMK2D, OSR1, OXSR1, SLC8A1, SLC9A1]
GO:0042991	transcription factor import into nucleus	Group09	9.62	5.00	[ATP1A2, CAMK2D, OSR1, OXSR1, SLC9A1]
GO:0042509	regulation of tyrosine phosphorylation of STAT protein	Group09	7.81	5.00	[ATP1A2, CAMK2D, OSR1, OXSR1, SLC9A1]
GO:0050716	positive regulation of interleukin-1 secretion	Group09	4.48	6.00	[ATP1A2, CAMK2D, GRK2, HDAC4, SLC8A1, SLC9A1]
GO:0043304	regulation of mast cell degranulation	Group09	11.43	4.00	[OSR1, OXSR1, XRCC5, XRCC6]
GO:0042306	regulation of protein import into nucleus	Group09	6.41	5.00	[ATP1A2, CAMK2D, HDAC4, SLC8A1, SLC9A1]
GO:0030316	osteoclast differentiation	Group09	9.76	4.00	[MSX2, OSR1, OXSR1, REG3A]
GO:0007260	tyrosine phosphorylation of STAT protein	Group09	4.90	5.00	[ATP1A2, B2M, CAMK2D, PARP1, SLC8A1]
GO:0030183	B cell differentiation	Group09	16.67	3.00	[ATP1A2, CAMK2D, SLC8A1]
GO:0045582	positive regulation of T cell differentiation	Group09	16.67	3.00	[ATP1A2, SLC8A1, SLC9A1]
GO:2000514	regulation of CD4-positive, alpha-beta T cell activation	Group09	15.79	3.00	[ATP1A2, CAMK2D, SLC8A1]
GO:0042503	tyrosine phosphorylation of Stat3 protein	Group09	4.39	5.00	[ATP1A2, CAMK2D, NOX1, SLC8A1, SLC9A1]
GO:0045830	positive regulation of isotype switching	Group09	6.25	4.00	[ATP1A2, CAMK2D, OSR1, OXSR1]
GO:0050702	interleukin-1 beta secretion	Group09	6.15	4.00	[ECE1, MSX2, OSR1, OXSR1]
GO:2000516	positive regulation of CD4-positive, alpha-beta T cell activation	Group09	12.50	3.00	[ATP1A2, CAMK2D, SLC8A1]
GO:0071549	cellular response to dexamethasone stimulus	Group09	12.00	3.00	[ATP1A2, CAMK2D, SLC8A1]
GO:0051133	regulation of NK T cell activation	Group09	11.54	3.00	[CAMK2D, PARP1, SLC9A1]
GO:0045580	regulation of T cell differentiation	Group09	11.54	3.00	[CAMK2D, PARP1, SLC9A1]
GO:0045191	regulation of isotype switching	Group09	5.56	4.00	[CAMK2D, HDAC4, PARP1, SLC9A1]
GO:0042348	NF-kappaB import into nucleus	Group09	5.56	4.00	[ATP1A2, CAMK2D, OSR1, OXSR1]
GO:0045620	negative regulation of lymphocyte differentiation	Group09	5.48	4.00	[ATP1A2, CAMK2D, OSR1, OXSR1]
GO:0070231	T cell apoptotic process	Group09	5.41	4.00	[CAMK2D, HDAC4, PARP1, SLC9A1]
GO:0046006	regulation of activated T cell proliferation	Group09	5.33	4.00	[CAMK2D, HDAC4, PARP1, SLC9A1]
GO:0002763	positive regulation of myeloid leukocyte differentiation	Group09	5.26	4.00	[CAMK2D, HDAC4, PARP1, SLC9A1]
GO:0045058	T cell selection	Group09	10.34	3.00	[ATP1A2, CAMK2D, SLC8A1]
GO:0001783	B cell apoptotic process	Group09	10.34	3.00	[ATP1A2, CAMK2D, SLC8A1]
GO:0033139	regulation of peptidyl-serine phosphorylation of STAT protein	Group09	10.00	3.00	[MSX2, OSR1, OXSR1]
GO:0045672	positive regulation of osteoclast differentiation	Group09	10.00	3.00	[ATP1A2, CAMK2D, SLC8A1]
GO:0030217	T cell differentiation	Group09	4.94	4.00	[OSR1, OXSR1, XRCC5, XRCC6]
GO:0034393	positive regulation of smooth muscle cell apoptotic process	Group09	4.94	4.00	[ATP1A2, FAS, HDAC4, SLC9A1]
GO:0033135	regulation of peptidyl-serine phosphorylation	Group09	4.82	4.00	[ATP1A2, CAMK2D, OSR1, OXSR1]
GO:0043306	positive regulation of mast cell degranulation	Group09	9.38	3.00	[ATP1A2, CAMK2D, SLC8A1]
GO:0033138	positive regulation of peptidyl-serine phosphorylation	Group09	9.09	3.00	[MSX2, OSR1, OXSR1]

GO:2000352	negative regulation of endothelial cell apoptotic process	Group09	4.60	4.00	[ATP1A2, GRK2, OSR1, OXSR1]
GO:0002891	positive regulation of immunoglobulin mediated immune response	Group09	4.49	4.00	[MSX2, OSR1, OXSR1, SLC8A1]
GO:0045621	positive regulation of lymphocyte differentiation	Group09	4.49	4.00	[ATP1A2, CAMK2D, OSR1, OXSR1]
GO:0042093	T-helper cell differentiation	Group09	8.33	3.00	[ATP1A2, OSR1, OXSR1]
GO:0071624	positive regulation of granulocyte chemotaxis	Group09	4.21	4.00	[ATP1A2, GRK2, OSR1, OXSR1]
GO:0048291	isotype switching to IgG isotypes	Group09	7.89	3.00	[MSX2, OSR1, OXSR1]
GO:0090023	positive regulation of neutrophil chemotaxis	Group09	7.69	3.00	[OSR1, OXSR1, SLC8A1]
GO:0002294	CD4-positive, alpha-beta T cell differentiation involved in immune response	Group09	4.08	4.00	[CAMK2D, HDAC4, PARP1, SLC9A1]
GO:0046641	positive regulation of alpha-beta T cell proliferation	Group09	7.32	3.00	[MSX2, OSR1, OXSR1]
GO:0042104	positive regulation of activated T cell proliferation	Group09	6.98	3.00	[OSR1, OXSR1, SLC8A1]
GO:0046640	regulation of alpha-beta T cell proliferation	Group09	6.67	3.00	[B2M, CAMK2D, PARP1]
GO:0000060	protein import into nucleus, translocation	Group09	6.67	3.00	[CAMK2D, PARP1, SLC9A1]
GO:0002902	regulation of B cell apoptotic process	Group09	6.38	3.00	[CAMK2D, PARP1, SLC9A1]
GO:0042307	positive regulation of protein import into nucleus	Group09	6.12	3.00	[MSX2, OSR1, OXSR1]
GO:0042508	tyrosine phosphorylation of Stat1 protein	Group09	5.45	3.00	[ATP1A2, SLC8A1, SLC9A1]
GO:0043380	regulation of memory T cell differentiation	Group09	5.36	3.00	[ATP1A2, CAMK2D, SLC8A1]
GO:0050704	regulation of interleukin-1 secretion	Group09	4.76	3.00	[PARP1, XRCC5, XRCC6]
GO:0090022	regulation of neutrophil chemotaxis	Group09	4.76	3.00	[ATP1A2, OSR1, OXSR1]
GO:0042506	tyrosine phosphorylation of Stat5 protein	Group09	4.55	3.00	[POLK, XRCC5, XRCC6]
GO:2000515	negative regulation of CD4-positive, alpha-beta T cell activation	Group09	4.41	3.00	[ATP1A2, CAMK2D, SLC9A1]
GO:0002204	somatic recombination of immunoglobulin genes involved in immune response	Group09	4.29	3.00	[ATP1A2, CAMK2D, SLC8A1]
GO:0045670	regulation of osteoclast differentiation	Group09	4.17	3.00	[OSR1, OXSR1, SLC8A1]
GO:0046632	alpha-beta T cell differentiation	Group09	4.17	3.00	[POLK, XRCC5, XRCC6]
GO:0042501	serine phosphorylation of STAT protein	Group09	4.11	3.00	[ATP1A2, CAMK2D, SLC8A1]
GO:0043379	memory T cell differentiation	Group09	4.00	3.00	[ATP1A2, B2M, SLC9A1]
GO:0070661	leukocyte proliferation	Group08	21.74	5.00	[ATP1A2, CAMK2D, HDAC4, SLC8A1, SLC9A1]
GO:0050670	regulation of lymphocyte proliferation	Group08	15.15	5.00	[ATP1A2, CAMK2D, GRK2, SLC8A1, SLC9A1]
GO:0060337	type I interferon signaling pathway	Group08	6.59	6.00	[ATP1A2, CAMK2D, GRK2, HDAC4, SLC8A1, SLC9A1]
GO:1904892	regulation of STAT cascade	Group08	9.62	5.00	[ATP1A2, CAMK2D, OSR1, OXSR1, SLC9A1]
GO:1904019	epithelial cell apoptotic process	Group08	7.81	5.00	[ATP1A2, CAMK2D, OSR1, OXSR1, SLC9A1]
GO:0051096	positive regulation of helicase activity	Group08	4.48	6.00	[ATP1A2, CAMK2D, GRK2, HDAC4, SLC8A1, SLC9A1]
GO:0097192	extrinsic apoptotic signaling pathway in absence of ligand	Group08	6.41	5.00	[ATP1A2, CAMK2D, HDAC4, SLC8A1, SLC9A1]
GO:0043122	regulation of I-kappaB kinase/NF-kappaB signaling	Group08	4.90	5.00	[ATP1A2, B2M, CAMK2D, PARP1, SLC8A1]
GO:1903707	negative regulation of hemopoiesis	Group08	4.39	5.00	[ATP1A2, CAMK2D, NOX1, SLC8A1, SLC9A1]
GO:0031663	lipopolysaccharide-mediated signaling pathway	Group08	15.00	3.00	[FBP1, HDAC4, PARP1]
GO:0002562	somatic diversification of immune receptors via germline recombination within a single locus	Group08	6.56	4.00	[FBP1, HDAC4, PARP1, SEM1]
GO:0046824	positive regulation of nucleocytoplasmic transport	Group08	11.54	3.00	[CAMK2D, PARP1, SLC9A1]
GO:0051054	positive regulation of DNA metabolic process	Group08	11.54	3.00	[CAMK2D, PARP1, SLC9A1]
GO:0050864	regulation of B cell activation	Group08	5.56	4.00	[CAMK2D, HDAC4, PARP1, SLC9A1]
GO:0048260	positive regulation of receptor-mediated endocytosis	Group08	5.41	4.00	[CAMK2D, HDAC4, PARP1, SLC9A1]

GO:0045348	positive regulation of MHC class II biosynthetic process	Group08	5.33	4.00	[CAMK2D, HDAC4, PARP1, SLC9A1]
GO:0043032	positive regulation of macrophage activation	Group08	5.26	4.00	[CAMK2D, HDAC4, PARP1, SLC9A1]
GO:0044130	negative regulation of growth of symbiont in host	Group08	4.94	4.00	[ATP1A2, FAS, HDAC4, SLC9A1]
GO:0042108	positive regulation of cytokine biosynthetic process	Group08	4.08	4.00	[CAMK2D, HDAC4, PARP1, SLC9A1]
GO:0046631	alpha-beta T cell activation	Group08	6.67	3.00	[B2M, CAMK2D, PARP1]
GO:0050829	defense response to Gram-negative bacterium	Group08	6.67	3.00	[CAMK2D, PARP1, SLC9A1]
GO:0050863	regulation of T cell activation	Group08	6.38	3.00	[CAMK2D, PARP1, SLC9A1]
GO:0071347	cellular response to interleukin-1	Group08	4.41	3.00	[ATP1A2, CAMK2D, SLC9A1]
GO:1903708	positive regulation of hemopoiesis	Group08	4.29	3.00	[FBP1, HDAC4, PARP1]
GO:0071887	leukocyte apoptotic process	Group08	4.17	3.00	[FBP1, HDAC4, PARP1]
GO:0016078	tRNA catabolic process	Group07	62.50	10.00	[CYC1, CYTB, UQCR10, UQCR11, UQCRB, UQCRC1, UQCRC2, UQCRFS1, UQCRH, UQCRQ]
GO:0043634	polyadenylation-dependent ncRNA catabolic process	Group07	5.77	18.00	[ATP1A2, CYC1, CYTB, FBP1, GART, GMPR2, HDAC4, HK2, PARP1, SEM1, UQCR10, UQCR11, UQCRB, UQCRC1, UQCRC2, UQCRFS1, UQCRH, UQCRQ]
GO:0043633	polyadenylation-dependent RNA catabolic process	Group07	5.75	18.00	[ATP1A2, CYC1, CYTB, FBP1, GART, GMPR2, HDAC4, HK2, PARP1, SEM1, UQCR10, UQCR11, UQCRB, UQCRC1, UQCRC2, UQCRFS1, UQCRH, UQCRQ]
GO:0071025	RNA surveillance	Group07	5.54	18.00	[ATP1A2, CYC1, CYTB, FBP1, GART, GMPR2, HDAC4, HK2, PARP1, SEM1, UQCR10, UQCR11, UQCRB, UQCRC1, UQCRC2, UQCRFS1, UQCRH, UQCRQ]
GO:0016074	snoRNA metabolic process	Group07	5.33	18.00	[ATP1A2, CYC1, CYTB, FBP1, GART, GMPR2, HDAC4, HK2, PARP1, SEM1, UQCR10, UQCR11, UQCRB, UQCRC1, UQCRC2, UQCRFS1, UQCRH, UQCRQ]
GO:0043628	ncRNA 3'-end processing	Group07	6.06	16.00	[ATP1A2, CYC1, CYTB, FBP1, HDAC4, HK2, PARP1, SEM1, UQCR10, UQCR11, UQCRB, UQCRC1, UQCRC2, UQCRFS1, UQCRH, UQCRQ]
GO:0016180	snRNA processing	Group07	5.48	16.00	[ATP1A2, CYC1, CYTB, FBP1, HDAC4, HK2, PARP1, SEM1, UQCR10, UQCR11, UQCRB, UQCRC1, UQCRC2, UQCRFS1, UQCRH, UQCRQ]
GO:0045006	DNA deamination	Group07	5.35	16.00	[ATP1A2, CYC1, CYTB, FBP1, HDAC4, HK2, PARP1, SEM1, UQCR10, UQCR11, UQCRB, UQCRC1, UQCRC2, UQCRFS1, UQCRH, UQCRQ]
GO:0090503	RNA phosphodiester bond hydrolysis, exonucleolytic	Group07	5.33	16.00	[ATP1A2, CYC1, CYTB, FBP1, HDAC4, HK2, PARP1, SEM1, UQCR10, UQCR11, UQCRB, UQCRC1, UQCRC2, UQCRFS1, UQCRH, UQCRQ]
GO:0031123	RNA 3'-end processing	Group07	4.97	16.00	[ATP1A2, CYC1, CYTB, FBP1, HDAC4, HK2, PARP1, SEM1, UQCR10, UQCR11, UQCRB, UQCRC1, UQCRC2, UQCRFS1, UQCRH, UQCRQ]
GO:0000469	cleavage involved in rRNA processing	Group07	9.17	11.00	[ATP1A2, CYC1, CYTB, SLC9A1, UQCR10, UQCR11, UQCRB, UQCRC1, UQCRFS1, UQCRH, UQCRQ]
GO:0034661	ncRNA catabolic process	Group07	7.06	12.00	[ATP1A2, CYC1, CYTB, NOX1, SLC9A1, UQCR10, UQCR11, UQCRB, UQCRC1, UQCRFS1, UQCRH, UQCRQ]
GO:0010608	posttranscriptional regulation of gene expression	Group07	6.94	12.00	[ATP1A2, CYC1, CYTB, NOX1, SLC9A1, UQCR10, UQCR11, UQCRB, UQCRC1, UQCRFS1, UQCRH, UQCRQ]
GO:0071027	nuclear RNA surveillance	Group07	10.99	10.00	[CYC1, CYTB, UQCR10, UQCR11, UQCRB, UQCRC1, UQCRC2, UQCRFS1, UQCRH, UQCRQ]
GO:0043144	snoRNA processing	Group07	10.64	10.00	[CYC1, CYTB, UQCR10, UQCR11, UQCRB, UQCRC1, UQCRC2, UQCRFS1, UQCRH, UQCRQ]
GO:0043488	regulation of mRNA stability	Group07	8.93	10.00	[CYC1, CYTB, UQCR10, UQCR11, UQCRB, UQCRC1, UQCRC2, UQCRFS1, UQCRH, UQCRQ]
GO:0043487	regulation of RNA stability	Group07	8.62	10.00	[CYC1, CYTB, UQCR10, UQCR11, UQCRB, UQCRC1, UQCRC2, UQCRFS1, UQCRH, UQCRQ]
GO:0016075	rRNA catabolic process	Group07	8.55	10.00	[CYC1, CYTB, UQCR10, UQCR11, UQCRB, UQCRC1, UQCRC2, UQCRFS1, UQCRH, UQCRQ]
GO:0090501	RNA phosphodiester bond hydrolysis	Group07	5.70	11.00	[CYC1, CYTB, IDH2, UQCR10, UQCR11, UQCRB, UQCRC1, UQCRC2, UQCRFS1, UQCRH, UQCRQ]
GO:0071035	nuclear polyadenylation-dependent rRNA catabolic process	Group07	4.10	12.00	[CYC1, CYTB, IDH2, PYGM, UQCR10, UQCR11, UQCRB, UQCRC1, UQCRC2, UQCRFS1, UQCRH, UQCRQ]
GO:0016073	snRNA metabolic process	Group07	8.00	6.00	[IDH2, UQCR10, UQCRB, UQCRC1, UQCRC2, UQCRH]
GO:0002335	mature B cell differentiation	Group06	15.00	3.00	[FBP1, HDAC4, PARP1]
GO:0042501	serine phosphorylation of STAT protein	Group06	6.56	4.00	[FBP1, HDAC4, PARP1, SEM1]
GO:0030097	hemopoiesis	Group06	5.56	4.00	[CAMK2D, HDAC4, PARP1, SLC9A1]
GO:0002312	B cell activation involved in immune response	Group06	5.41	4.00	[CAMK2D, HDAC4, PARP1, SLC9A1]
GO:0043331	response to dsRNA	Group06	5.33	4.00	[CAMK2D, HDAC4, PARP1, SLC9A1]

GO:0002285	lymphocyte activation involved in immune response	Group06	5.26	4.00	[CAMK2D, HDAC4, PARP1, SLC9A1]
GO:0030098	lymphocyte differentiation	Group06	4.71	4.00	[FBP1, HDAC4, HK2, SEM1]
GO:0002521	leukocyte differentiation	Group06	4.65	4.00	[FBP1, HDAC4, HK2, SEM1]
GO:0002313	mature B cell differentiation involved in immune response	Group06	4.26	4.00	[FBP1, HDAC4, HK2, SEM1]
GO:0030183	B cell differentiation	Group06	7.69	3.00	[FBP1, HDAC4, SEM1]
GO:0007162	negative regulation of cell adhesion	Group06	4.08	4.00	[CAMK2D, HDAC4, PARP1, SLC9A1]
GO:0002323	natural killer cell activation involved in immune response	Group06	6.38	3.00	[FBP1, HDAC4, SEM1]
GO:0048534	hematopoietic or lymphoid organ development	Group06	4.62	3.00	[FBP1, HDAC4, SEM1]
GO:0042113	B cell activation	Group06	4.62	3.00	[FBP1, HDAC4, SEM1]
GO:0042100	B cell proliferation	Group06	4.29	3.00	[FBP1, HDAC4, PARP1]
GO:0043330	response to exogenous dsRNA	Group06	4.17	3.00	[FBP1, HDAC4, PARP1]
GO:0060333	interferon-gamma-mediated signaling pathway	Group05	7.61	7.00	[B2M, CAMK2D, HCK, HLA-E, HLA-G, IFNGR1, SOCS3]
GO:0071346	cellular response to interferon-gamma	Group05	4.58	7.00	[B2M, CAMK2D, HCK, HLA-E, HLA-G, IFNGR1, SOCS3]
GO:0034341	response to interferon-gamma	Group05	4.02	7.00	[B2M, CAMK2D, HCK, HLA-E, HLA-G, IFNGR1, SOCS3]
GO:0002480	antigen processing and presentation of exogenous peptide antigen via MHC class I, TAP-independent	Group05	30.00	3.00	[B2M, HLA-E, HLA-G]
GO:0002479	antigen processing and presentation of exogenous peptide antigen via MHC class I, TAP-dependent	Group05	5.00	4.00	[B2M, HLA-E, HLA-G, SEM1]
GO:0042590	antigen processing and presentation of exogenous peptide antigen via MHC class I	Group05	4.82	4.00	[B2M, HLA-E, HLA-G, SEM1]
GO:0045730	respiratory burst	Group04	11.43	4.00	[OSR1, OXSR1, XRCC5, XRCC6]
GO:0051707	response to other organism	Group04	10.71	3.00	[LIG1, PARP1, XRCC6]
GO:0045785	positive regulation of cell adhesion	Group04	4.94	4.00	[OSR1, OXSR1, XRCC5, XRCC6]
GO:0048771	tissue remodeling	Group04	4.76	3.00	[PARP1, XRCC5, XRCC6]
GO:0071216	cellular response to biotic stimulus	Group04	4.55	3.00	[POLK, XRCC5, XRCC6]
GO:0070661	leukocyte proliferation	Group04	4.17	3.00	[POLK, XRCC5, XRCC6]
GO:0032675	regulation of interleukin-6 production	Group03	6.50	8.00	[CAMK2D, HDAC4, MAP3K5, NOX1, SKP2, TGM2, XRCC5, XRCC6]
GO:0032642	regulation of chemokine production	Group03	6.25	8.00	[CAMK2D, HDAC4, MAP3K5, NOX1, SKP2, TGM2, XRCC5, XRCC6]
GO:0032635	interleukin-6 production	Group03	4.37	8.00	[CAMK2D, HDAC4, MAP3K5, NOX1, SKP2, TGM2, XRCC5, XRCC6]
GO:0032755	positive regulation of interleukin-6 production	Group03	7.50	6.00	[CAMK2D, HDAC4, MAP3K5, NOX1, SKP2, TGM2]
GO:0038110	interleukin-2-mediated signaling pathway	Group02	20.00	3.00	[GNPNAT1, MGAT1, RENBP]
GO:0071352	cellular response to interleukin-2	Group02	8.33	3.00	[GNPNAT1, MGAT1, RENBP]
GO:0034250	positive regulation of cellular amide metabolic process	Group02	7.50	3.00	[GNPNAT1, MGAT1, RENBP]
GO:0071353	cellular response to interleukin-4	Group01	42.86	3.00	[ECE1, IDE, XPNPEP1]
GO:0070670	response to interleukin-4	Group01	7.69	3.00	[ECE1, IDE, XPNPEP1]
GO:0098586	cellular response to virus	Group00	6.12	3.00	[MAP3K5, NOX1, PARP1]
KEGGID	KEGGTerm	KEGGGr	% Associated Genes	Nr. Genes	Associated Genes Found
Has:04662	B cell receptor signaling pathway	Group03	54.21	3.00	[MAP3K5, NOX1, PARP1]
Has:04670	Leukocyte transendothelial migration	Group03	5.62	8.00	[CD79B, CD97A, FCGR2B, IGH, IL4, MAPK3, NFATC1, PLCG2]
Has:04660	T cell receptor signaling pathway	Group03	7.49	8.00	[CD79B, CD97A, FCGR2B, IGH, IL4, MAPK3, NFATC1, PLCG2]
Has:04612	Antigen processing and presentation	Group03	4.27	5.00	[CD79B, IGH, MAPK3, NFATC1, PLCG2]
Has:04060	Cytokine-cytokine receptor interaction	Group03	3.52	10.00	[CARD11, CD22, CD79B, CD97A, FCGR2B, IGH, IL4, MAPK3, NFATC1, PLCG2]
Has:04650	Natural killer cell mediated cytotoxicity	Group03	4.50	9.00	[CARD11, CD22, CD79B, CD97A, FCGR2B, IGH, IL4, MAPK3, NFATC1, PLCG2]
Has:04668	TNF signaling pathway	Group02	37.85	10.00	[CHUK, HRAS1, JAK, MAPK10, MAPK14, MAPK3K5, MAPK8, MFKB1, RIPK1, TNF]
Has:04010	MAPK signaling pathway	Group02	8.62	10.00	[CHUK, HRAS1, JAK, MAPK10, MAPK14, MAPK3K5, MAPK8, MFKB1, PIK3CA, TNF]

Has:04064	NF-κ B signaling pathway	Group02	5.27	8.00	[CHUK, HRAS1, JAK, MAPK10, MAPK14, MAPK3K5, MAPK8, MFKB1, PIK3CA]
Has:04151	PI3K-Akt signaling pathway	Group02	4.66	5.00	[CHUK, HRAS1, JAK, MAPK10, MAPK14, MAPK3K5]
Has:05310	asthma	Group01	5.98	3.00	[CD40, IL4, PRG2]
Has:05133	Pertussis	Group00	8.01	3.00	[ITGB2, MAPK14, TLR4]

Table S4 The dock results of 20 compounds to the preotein 3v6s and 4bib

mol	receptor	rseq	mseq	S	rmsd_refin	FP:PLIF	PLIF_ligidx	E_conf	E_place	E_score1	E_refine	E_score2
5	3v6s	1	5	-10.3391	2.6854105	7 14 9 10 5	[31,8,17,17,[11,16,15,14,13,12]]	46.5931	-59.2551	-12.5713	-68.6011	-10.3391
2	4bib	1	2	-10.29957	1.4154652	7 1 12 13 14 8 6	[[11,12,46,10,52,55,56,9,13,15],[4,7,8,9,10,5,4,7,8,9,10,5],17,17,[17,18],[5,21,58,1,2,3,4,6,7,10],[34,36,37,38,39,40,42,43,65,69]]	76.42289	-144.943	-14.2722	-61.5939	-10.2996
5	3v6s	1	5	-10.0292	1.8983506	7 8 14 24 9 4 25 26	[44,44,17,17,8,18,18,[4,7,8,9,10,5]]	45.11908	-120.496	-14.6078	-62.0732	-10.0292
5	4bib	1	5	-9.963227	1.3998624	7 1 11 12 14 33 8 6	[[9,11,12,10,16,50,53,56,57,14],[4,7,8,9,10,5],[4,7,8,9,10,5],17,18,18,[1,5,6,21,2,3,4,7,17,19],[36,40,41,42,43,64,68,69,70,71]]	55.3295	-123.202	-13.2606	-49.1488	-9.96323
5	3v6s	1	5	-9.700732	1.2840828	1 27 14 5	[30,30,[17,19],[1,6,5,4,3,2]]	51.67727	-115.297	-15.943	-50.5603	-9.70073
1	3v6s	1	1	-9.687674	2.6197438	1 2	[30,29]	76.73344	-86.9623	-10.7745	-49.3919	-9.68767
1	3v6s	1	1	-9.605123	1.2462834	1 2	[30,29]	70.6914	-57.4618	-11.4426	-51.6914	-9.60512
1	3v6s	1	1	-9.593266	1.1859188	2	29	72.66728	-56.2611	-10.9032	-47.6317	-9.59327
2	3v6s	1	2	-9.587329	1.8712076		[]	65.34726	-108.279	-13.6831	-56.8819	-9.58733
5	4bib	1	5	-9.565395	1.9617903	7 1 3 19 31 32 4 5 6	[[15,16,49,50,53,56,58,24,26,27],[11,16,15,14,13,12],[5,6,10,53,4,7,8,9,3,11],[4,7,8,9,10,5,1,6,5,4,3,2],31,31,19,19,[17,51,2,3,4,45,67,37,38,52]]	36.70976	-134.602	-16.1091	-54.9425	-9.5654
5	3v6s	1	5	-9.528144	2.3400955	9 10 5	[17,17,[36,41,40,39,38,37]]	54.4725	-94.0109	-12.4791	-57.5031	-9.52814
2	3v6s	1	2	-9.460021	1.902523	9 10 11 5	[17,17,[4,7,8,9,10,5],[34,39,38,37,36,35]]	88.65537	-117.926	-13.8272	-62.5541	-9.46002
2	4ehz	1	2	-9.457643	1.9870707	1 2 3 15 17 6 14 16	[[10,11,12,13,46,52,9,14,16,47],[4,7,8,9,10,5],[4,7,8,9,10,5],33,33,[53,22,23,54,26,58,21,20,25,28],19,19,[44,51,50,19,35,36,63,34,64,1]]	78.71437	-140.072	-13.6206	-61.7094	-9.45764
2	4ehz	1	2	-9.442382	1.7845579	1 2 3 4 19 15 17 6 1	[[10,11,15,16,49,52,12,14,48,20],[4,7,8,9,10,5],[4,7,8,9,10,5],17,27,[33,33],33,[22,23,53,54,21,20,25,26,28,30],19,19]	81.29775	-127.734	-13.6852	-63.2438	-9.44238
2	4ehz	1	2	-9.430423	1.4462125	1 20 6 10 11	[[56,25,28,29,32,34,35,63,36,37],[68,70,35,36,37,38,40,41,63,64],[2,44,51,1,19,50,3,5,6,20],[27,30],27]	79.49385	-130.547	-14.4939	-47.3817	-9.43042
5	3v6s	1	5	-9.402	2.2910757	12 13 6	[[34,35,36,41,65,68,69,40,42,43],22,30]	43.06151	-121.759	-13.9423	-53.9551	-9.402
2	3v6s	1	2	-9.398039	2.8289626	12 13 7 8 14 9 10 5	[[64,58,35,36,63,68,37,40,41,66],30,31,31,8,17,17,[11,16,15,14,13,12]]	189.3934	-104.455	-14.5628	-57.7755	-9.39804
2	4ehz	1	2	-9.343605	1.1939321	1 2 21 19 15 17 6 14	[[15,16,48,49,52,58,11,14,10,20],[4,7,8,9,10,5],[1,6,5,4,3,2],27,33,33,[22,23,30,53,54,21,20,24,25,26],19,19]	77.56097	-149.243	-16.9678	-61.4765	-9.34361
2	4bib	1	2	-9.316828	2.2577522	7 2 17 3 18 9 10 8 6	[[19,44,51,1,2,21,28,32,34,35],30,30,[21,22,53,4,5,1,2,3,6,7],[31,31,31,31,31],29,29,[5,56,1,2,3,4,6,7,8,9],[11,12,13,14,15,16,31,46,47,48]]	71.26305	-140.242	-14.463	-58.991	-9.31683
2	3v6s	1	2	-9.315962	0.99846607	15 6 16 5	[[4,7,8,9,10,5],29,29,[34,39,38,37,36,35]]	78.37252	-153.658	-15.7633	-48.8005	-9.31596
2	4ehz	1	2	-9.297028	1.2107126	1 19 22 20 6 10 11	[[56,70,25,39,43,65,34,38,42,69],19,19,[67,41,68,51,38,42,43,44,69,71],[1,5,6,19,20,21,22,27,53,2],30,30]	74.28674	-101.37	-14.7222	-58.9951	-9.29703
2	4bib	1	2	-9.285941	1.1030242	7 1 2 3 8 4 5 6	[[19,44,1,21,32,33,51,2,6,20],[1,6,5,4,3,2],30,[49,53,58,21,22,9,10,16,4,5],[4,5,56,2,3,6,7,8,9,10],27,27,[12,13,14,15,31,46,47,62,11,16]]	76.46396	-119.711	-14.7553	-62.9768	-9.28594
5	4ehz	1	5	-9.275881	1.6246018	1 2 26 31 6 32 10 11	[[15,16,49,50,53,57,11,14,59,10],[4,7,8,9,10,5,4,7,8,9,10,5],18,18,[22,23,30,54,55,62,21,20,24,25],[1,6,5,4,3,2],19,19]	45.0336	-136.302	-14.5718	-56.5941	-9.27588
2	4bib	1	2	-9.263681	2.1111796	7 3 8 6	[[5,8,9,1,6,10,19,20,49,52],[51,1,19,33,44,56,2,6,25,69],[71,2,3,4,17,43,44,50,65,1],[35,36,37,38,39,40,41,42,64,66]]	67.62508	-106.719	-13.3984	-53.5368	-9.26368
2	3v6s	1	2	-9.253801	1.2531853	15 6 16 5	[[4,7,8,9,10,5],29,29,[34,39,38,37,36,35]]	77.81842	-145.09	-16.9166	-48.4209	-9.2538

2	4bib	1	2	-9.220719	1.2086868	7 1 11 3 8 4 6	[[9,10,8,11,5,12,46,52,6,13],[4,7,8,9,10,5,4,7,8,9,10,5],[4,7,8,9,10,5],[1,19,25,33,51,56,6,20,52,2],[21,53,1,2,3,4,5,6,17,44],23,[34,35,36,37,38,40,41,63,64,68]]	74.09794	-75.1854	-16.5199	-52.5227	-9.22072
5	4ehz	1	5	-9.187481	1.7312806	1 2 3 26 31 19 22 20	[[12,13,47,48,53,57,11,14,10,20],[4,7,8,9,10,5],[4,7,8,9,10,5],18,18,27,27,[31,12,13,14,48,59,60,63,11,15],19,19]	51.21276	-130.667	-16.2134	-56.9252	-9.18748
13	4ehz	1	13	-9.130926	1.2166187	33 21 4 6 51	[[16,17,18,21,22,23,49,52,53,54],[1,6,5,4,3,2],34,[13,14,43,47,7,9,11,12,41,48],[32,64,56,62,63,26,30,31,61]]	129.1914	-78.2234	-12.6836	-47.915	-9.13093
5	4ehz	1	5	-9.119332	2.0696509	1 2 33 21 4 26 31 6	[[15,16,49,50,53,11,14,59,10,20],[4,7,8,9,10,5,4,7,8,9,10,5],[35,36,41,65,68,71,40,42,43,39],[1,6,5,4,3,2],17,18,18,[22,23,30,54,55,21,20,24,25,26],19]	47.56004	-109.118	-15.4824	-56.5128	-9.11933
1	4bib	1	1	-9.110381	1.2464406	1 2 3 4 5 6	[[1,6,5,4,3,2],30,[21,43,46,22,5,8,9,10,16,3],27,27,[12,13,14,15,31,40,41,55,11,16]]	66.28761	-138.913	-13.1992	-61.0788	-9.11038
13	4ehz	1	13	-9.079516	2.345716	45 47 1 2 33 6 18	[35,35,[1,2,5,6,11,45,48,3,4,34],[1,6,5,4,3,2],[22,23,28,29,37,53,54,55,59,60],[42,51,9,20,43,8,10,12,15,19],[65,33,16,24,46,56,25,30,12,15]]	129.0694	-85.7156	-15.1645	-40.9757	-9.07952
1	4bib	1	1	-9.07751	2.7173312	7 1 2 8 6	[[19,32,56,58,57,65,1,21,28,36],[1,6,5,4,3,2],30,[4,5,2,3,6,7,8,9,10,17],[12,13,14,15,31,40,41,55,11,16]]	67.87024	-124.677	-12.4403	-55.7664	-9.07751
5	4ehz	1	5	-9.056802	1.4423625	34 1 20 30 6 10 11	[[71,37,38,39,40,41,42,43,44,72],[25,32,34,35,57,65,33,36,64,66],[36,34,35,37,38,41,65,66,32,33],27,[1,2,19,45,52,60,6,21,26,27],30,30]	52.23127	-178.941	-13.5576	-40.3016	-9.0568
5	4ehz	1	5	-9.051628	1.8782761	1 35 36 6 10 37	[[54,58,11,12,13,14,15,16,31,49],[36,41,40,39,38,37],18,[57,53,24,25,29,56,6,19,20,27],30,[36,41,40,39,38,37]]	42.55458	-104.178	-13.6449	-58.284	-9.05163
1	3v6s	1	1	-9.048012	2.397402	3 4 5	[[11,16,15,14,13,12],29,[11,16,15,14,13,12]]	92.84176	-95.0701	-11.4097	-49.6547	-9.04801
5	4bib	1	5	-9.026092	1.5510459	7 1 20 21 8	[[57,8,9,10,50,5,6,20,25,53],[1,6,5,4,3,2,4,7,8,9,10,5],44,44,[1,2,3,4,6,17,19,45,51,52]]	41.66835	-112.734	-13.4398	-50.9324	-9.02609
13	4ehz	1	13	-9.018941	2.6008177	2 50 33 28 29 12 13	[[1,6,5,4,3,2],[16,17,22,23,42,49,55,53,54,18],[24,29,37,60,21,25,26,28,56,57],33,33,34,34,[13,14,46,47,12,15,48,49,16,53],33,33]	121.547	-105.532	-12.8047	-50.7749	-9.01894
1	3v6s	1	1	-8.97676	1.8919984	6 7 8 5	[27,31,31,[11,16,15,14,13,12]]	87.99085	-108.761	-12.2098	-35.4475	-8.97676
6	4ehz	1	6	-8.943616	1.4533267	1 18	[[39,15,53,14,16,20,40,30,28,51],[45,5,6,10,12,35,37,44,22,9]]	79.06439	-82.0515	-13.823	-41.3839	-8.94362
13	3v6s	1	13	-8.941723	2.271754	5	[[1,6,5,4,3,2]]	128.3948	-91.8712	-12.4071	-43.4205	-8.94172
10	3v6s	1	10	-8.934802	2.3746178	7 14 34 35	[9,[6,8],6,10]	96.14289	-74.9749	-14.4136	-42.1324	-8.9348
13	4ehz	1	13	-8.933168	1.3236299	1 33 26 15 17 6	[[60,24,29,37,17,18,19,50,21,22],[3,4,5,7,11,14,35,36,39,41],33,[8,13],[8,13],[49,55,23,16,46,12,15,17,22,13]]	139.4369	-117.025	-15.1169	-46.6714	-8.93317
13	4ehz	1	13	-8.911814	1.9021779	1 24 33 26 15 17 6	[[29,60,55,17,22,23,37,18,24,53],[15,20,19,18,17,16],[1,4,5,6,7,11,14,38,40,41],33,[8,13],[8,13],[53,15,19,20,46,51,12,16,17,18]]	136.7408	-111.163	-12.719	-47.2809	-8.91181
5	4nyi	1	5	-8.863739	2.9817309	3 8 9 10 4 5 6 7 11	[[48,59,60,13,14,27,31,58,63,12],[11,16,15,14,13,12],18,18,21,21,[18,17,51,2,3,45,4,7,5,8],17,17,[71,43,69,70,68,40,41,42,2,45]]	39.12756	-40.7697	-15.2055	-56.5341	-7.56374
13	3v6s	1	13	-8.858524	1.6127367	12 13	[[31,52,59,63,27,28,58,62,16,17],34]	118.8238	-117.984	-12.3849	-52.1735	-8.85852
5	4bib	1	5	-8.833965	3.2376645	7 1 2 17 35 3 8 4 5	[[1,19,21,28,34,64,68,69,71,2],[1,6,5,4,3,2],30,30,[22,23,54,55,62,64,71,21,30,68],[50,21,54,7,8,9,10,16,46,4],[4,5,53,2,3,6,7,8,9,10],27,27,[12,13,14,15,31,47,48,63,11,16],[11,16,15,14,13,12]]	31.16391	-103.695	-13.6324	-62.9139	-8.83397

1	4ehz	1	1	-8.810451	3.0662725	1 2 3 4 5 6 7 8	[[3,4,5,2,7,17,44,1,6,10],[4,7,8,9,10,5],[4,7,8,9,10,5],31,31,[29,48,49,61,24,25,45,53,20,23],29,29]	66.68647	-108.481	-12.284	-48.3493	-8.81045
5	4bib	1	5	-8.800234	1.9443418	3 36 37 8 6	[[50,53,56,57,58,9,10,11,16,5],44,[36,41,40,39,38,37],[54,59,8,9,10,11,12,13,14,15],[2,3,4,7,17,18,45,51,1,5]]	54.69545	-119.666	-12.7935	-38.6135	-8.80023
13	3v6s	1	13	-8.795109	1.160668	6	22	140.8237	-92.0526	-10.8194	-39.1605	-8.79511
1	4bib	1	1	-8.778168	1.4328015	7 1 9 10 8 4 5 6	[[58,64,19,32,38,60,1,6,21,34],[1,6,5,4,3,2],29,29,[4,5,49,3,6,7,8,9,10,17],27,27,[12,13,14,15,31,40,41,55,11,16]]	83.17808	-126.873	-14.5266	-57.0522	-8.77817
1	4bib	1	1	-8.769168	1.6310655	1 11 3 12 13 14 15 9	[[4,7,8,9,10,5],[4,7,8,9,10,5],[25,28,29,49,61,68,24,48,60,1],17,17,18,[11,16,15,14,13,12],27,27,[1,5,6,21,51,58,2,3,4,7]]	76.13235	-102.342	-11.8976	-53.9757	-8.76917
1	4bib	1	1	-8.764303	3.6467867	7 1 3 8 4 5 6 16	[[57,32,58,1,6,19,21,28,34,60],[1,6,5,4,3,2],[43,21,46,8,9,10,16,4,5,6],[4,5,45,2,3,6,7,8,9,10],27,27,[12,13,14,15,31,40,41,55,11,16],[11,16,15,14,13,12]]	64.34872	-78.6999	-11.8822	-59.1738	-8.7643
13	3v6s	1	13	-8.757544	1.3744901	39	29	125.0579	-103.109	-10.9816	-53.0688	-8.75754
10	4ehz	1	10	-8.730774	0.97871232	1 9 12 13 44 20 10	[[2,19,21,22,8,9,23,25,29,38],[48,18,47,1,6,11,40,50,2,16],12,12,10,[29,38,52,9,25,10,22,30,8,21],3]	96.99109	-130.948	-14.7439	-50.8633	-8.73077
13	3v6s	1	13	-8.730613	1.4070178	5	[[1,6,5,4,3,2]]	129.8347	-106.18	-10.9569	-39.8833	-8.73061
1	4nyi	1	1	-8.72745	1.3460664	1 2 3 4 5 6 7	[[4,7,8,9,10,5],[51,5,6,10,21,40,45,1,19,20],[19,20,26,32,45,48,50,51,56,57],28,28,[14,15,31,42,55,13,16,43,11,12],15]	46.30401	-87.1252	-12.629	-40.255	-7.42745
13	3flw	1	13	-8.723788	2.0222423	30 10 11 13	[33,29,29,28]	121.2423	-56.4008	-10.9314	-51.8455	-8.72379
2	4nyi	1	2	-8.693251	3.3128204	3 8 9 10 4 5 6 7 11	[[47,58,13,14,31,57,62,12,15,27],[11,16,15,14,13,12],18,18,21,21,[17,18,50,3,44,2,4,7,5,8],17,17,[68,36,37,38,40,2,39,44,50,51]]	69.98482	-72.5652	-12.1352	-55.9725	-7.39325
13	4bib	1	13	-8.66641	1.7854599	7 1 3 20 21 49 50 39	[[56,24,25,48,26,30,57,61,13,14],[15,20,19,18,17,16],[47,11,14,41,45,48,7,13,54,5],34,34,29,29,[28,59,21,27,29,31,37,52,58,60],[16,44,49,55,42,69,10,15,17,22]]	118.5816	-124.588	-12.218	-45.4973	-8.66641
6	3v6s	1	6	-8.649964	3.2707293	9	34	76.0596	-63.2428	-13.4191	-43.5868	-8.64996
5	4nyi	1	5	-8.646158	1.9549513	2 8 9 10 6 7 11 28 1	[[47,53,56,20,57,59,23,24,26,29],[11,16,15,14,13,12],18,18,[17,18,51,3,2,4,45,7,5,8],[2,17],17,[51,71,43,70,42,69,45,2,3,17],[71,40,42,43,70,2,45,52,3,17]]	41.11273	-102.708	-14.074	-57.1541	-7.34616
6	4ehz	1	6	-8.638791	1.6985532	1 33 38 39 40 41 15	[[19,41,4,7,8,17,36,3,2,21],[14,15,20,24,25,39,50,26,31,32],32,32,34,34,30,[7,8,17,36,40,39,15,16,30,50]]	78.05483	-105.305	-14.4803	-53.0975	-8.63879
1	4ehz	1	1	-8.627935	2.8032808	1 9 6 10 11	[[29,33,49,59,67,37,69,19,20,24],[40,41,48,24,27,29,52,20,23,25],[32,56,58,57,21,1,6,19,20,22],30,30]	68.09535	-83.5753	-12.0315	-42.6789	-8.62794
13	3flw	1	13	-8.62774	1.4214169	10 21	[29,32]	124.3281	-95.3594	-11.1436	-47.511	-8.62774
1	4ehz	1	1	-8.621923	1.9579926	1 2 12 13 6 14	[[3,4,5,2,7,17,18,44,1,6],[4,7,8,9,10,5],31,31,[48,49,67,45,24,25,29,50,53,20],27]	66.02969	-73.9764	-12.5585	-47.6235	-8.62192
6	4ehz	1	6	-8.610743	1.184377	2 33 9 40 6	[[1,6,5,4,3,2],[14,15,20,24,25,39,50,51,56,26],[6,10,35,37,5,9,11,12,1,18],34,[7,8,9,17,36,40,15,16,39,30]]	77.9903	-101.914	-13.662	-49.0969	-8.61074
1	4ehz	1	1	-8.608733	1.5206105	1 6 10 11	[[29,33,49,59,67,37,69,19,20,24],[32,56,58,57,21,1,6,19,20,22],30,30]	68.64891	-134.072	-12.0601	-40.8324	-8.60873
10	3v6s	1	10	-8.60135	1.5334399	7 8 4 25 35	[9,9,4,4,10]	102.9134	-94.4658	-13.3858	-43.8749	-8.60135
6	4ehz	1	6	-8.590553	1.2629184	1 2 28 20 15 7 8	[[5,6,9,10,35,1,4,44,11,12],[4,7,8,9,10,5],32,[48,23,46,47,49,18,1,2,6,22],[30,34],30,30]	74.00261	-132.636	-14.8754	-53.2201	-8.59055

6	4ehz	1	6	-8.575705	3.0613594	1 42	[[28,52,53,34,60,20,38,51,55,29],[35,45,22,44,1,5,6,10,18,46]]	71.04492	-109.997	-14.4263	-45.7551	-8.5757
6	4bib	1	6	-8.560762	0.87446344	7 1 3 19 22 38 20 39	[[2,3,4,5,6,44,1,10,18,22],[4,7,8,9,10,5],[38,12,37,11,13,14,51,9,15,16],[11,16,15,14,13,12],33,33,34,[17,19,41,3,23,48,2,4,7,21],[1,6,5,4,3,2],[50,8,9,10,11,12,13,14,15,16],34]	89.73334	-101.68	-13.214	-38.0813	-8.56076
10	3v6s	1	10	-8.529781	1.632526	13 36 31 37 26 5 38	[5,5,10,10,[24,28,32,33,31,27],[7,23,22,21,19,20,22,25,29,30,26,23,7,23,22,21,19,20],[22,25,29,30,26,23]]	92.32488	-98.2706	-13.5612	-45.9703	-8.52978
13	3flw	1	13	-8.527034	2.0795808	23 13	[32,14]	127.5791	-94.9185	-11.3569	-31.4079	-8.52703
10	3v6s	1	10	-8.522806	1.890083	31 37 26 5 38	[10,10,[24,28,32,33,31,27],[7,23,22,21,19,20,22,25,29,30,26,23,7,23,22,21,19,20],[22,25,29,30,26,23]]	109.543	-84.6543	-13.7444	-40.0311	-8.52281
13	4bib	1	13	-8.505051	1.2400309	7 1 20 21 49 39 6	[[56,24,25,48,18,21,26,30,33,57],[15,20,19,18,17,16],34,34,29,[28,59,21,27,29,31,37,52,58,60],[1,2,3,4,34,35,36,39,66,67]]	118.0739	-105.373	-13.0175	-45.3032	-8.50505
13	4bib	1	13	-8.485691	1.87994	7 1 49 50 39 8	[[56,24,25,48,21,26,30,57,61,13],[15,20,19,18,17,16],29,29,[28,59,63,21,27,29,31,37,52,58],[16,44,49,53,40,42,10,15,17,22]]	121.7166	-99.1408	-11.9919	-47.6611	-8.48569
5	3flw	1	5	-8.480024	4.0305562	1 3 16 17	[[19,52,53,1,6,20,25,28,32,57],[4,7,8,9,10,5],27,27]	40.38177	-109.519	-15.2519	-48.3079	-8.48002
bulit-	4nyi	1	1	-8.47923	0.36303607	1.6585121	1 2 3 4 5 6 7 8 9 10 11 12 13 14 15 16 17 18 19 20 21 22 23 24 25 26 27 28 29 30 31 32 33 34 35 36 37 38	[4,4,4,4,	-865.363	-117.234	-38.3732	-207.328
5	3flw	1	5	-8.473818	2.4662719	1 3 19 20 12	[[19,52,53,20,24,25,56,57,1,6],[4,7,8,9,10,5],29,29,30]	39.05922	-137.365	-14.1402	-46.3667	-8.47382
6	3v6s	1	6	-8.471058	1.8454186	28	30	78.02773	-87.6596	-14.9254	-38.0116	-8.47106
10	3v6s	1	10	-8.462091	2.069129	1 13 36 6 31 37 5	[4,5,5,5,10,10,[7,23,22,21,19,20]]	103.1713	-66.8839	-14.9366	-44.961	-8.46209
6	3v6s	1	6	-8.461165	0.93991858	17	[[4,7,8,9,10,5]]	80.88761	-85.234	-13.031	-40.7393	-8.46117
1	4ehz	1	1	-8.456502	1.6388334	1 15 6 14 16	[[62,25,28,35,49,53,68,34,36,60],18,[32,56,58,57,21,46,50,1,6,19],27,27]	82.2449	-127.631	-12.0697	-35.9109	-8.4565
6	3flw	1	6	-8.435455	2.0841556		[]	79.7676	-88.6226	-11.9493	-43.8596	-8.43546
2	3flw	1	2	-8.413394	2.9305968	19 20	[17,17]	84.0777	-123.199	-12.3542	-46.7623	-8.41339
6	3flw	1	6	-8.410733	1.7925255	1	[[31,32,51,56,26,27,52,58,36]]	80.10929	-72.8214	-13.2886	-42.5723	-8.41073
2	4nyi	1	2	-8.410168	2.1977198	2 6 7 11 12 15 16	[[49,16,53,8,9,45,4,7,10,11],[61,55,24,29,30,60,57,52,56,26],29,29,[71,42,43,70,19,55,56,60,65,24],33,[23,30,54,61,29,24,60,22,25,28]]	70.22295	-71.1118	-12.1725	-31.5504	-7.11017
10	4nyi	1	10	-8.406959	2.5936363	42 43 2 3 6	[[3,4],4,[34,3,35,7,13,42,4,43,14,44],[29,35,43,4,9,10,22,25,30,52],[12,33,41,53,32,11,31,40,24,28]]	98.32186	-84.2761	-16.9438	-34.8622	-7.10696
11	4bib	1	11	-8.398315	1.5005242	7 1 48 14 8 4 6	[[44,39,1,19,25,28,40,2,6,20],[1,6,5,4,3,2],18,18,[4,5,10,3,6,7,8,9,18,34],30,[13,14,15,16,31,32,51,52,11,12]]	55.66406	-130.242	-14.9415	-47.9844	-8.39832
1	4nyi	1	1	-8.387784	1.3893703	2 8 9 10 6 7 11 12	[[40,48,45,49,53,24,25,29,30,50],[11,16,15,14,13,12],18,18,[17,18,44,3,2,4,38,7,5,8],17,17,[64,2,38,44,58,66,3,17,36,60]]	71.9772	-82.7187	-12.0124	-48.6768	-7.08778
5	3flw	1	5	-8.38432	1.1220328	1 24 15 25 4 6 13	[[71,41,42,43,45,52,68,70,40,69],21,[4,7,8,9,10,5],[4,7,8,9,10,5],29,30,27]	46.96635	-77.6029	-13.7812	-42.241	-8.38432
10	4nyi	1	10	-8.377589	1.9365618	2 4 5 6 7 11	[[40,50,27,46,7,23,26,49,20,22],2,2,[6,37,43,48,18,4,14,35,16,34],[3,4],[3,4]]	88.93385	-113.058	-14.7365	-40.9541	-7.07759
20	4nyi	1	20	-8.37651	1.5316105	1 2 4 5 6	[[4,7,8,9,10,5],[46,5,6,10,20,21,40,1,19,39],28,28,[14,31,51,35,13,12,15,11,16,34]]	45.38717	-80.1669	-12.7619	-37.7463	-7.07651
10	4bib	1	10	-8.376103	1.7267442	1 3 22 31 32 8 4 5 6	[[7,23,22,21,19,20,22,25,29,30,26,23],[40,11,1,27,47,48,50,31,2,19],6,9,9,[19,20,36,51,5,28,46,2,7,8],6,6,[3,4,13,14,34,35,42,45,5,15]]	89.14577	-148.563	-14.4145	-47.3848	-8.3761

1	4nyi	1	1	-8.339061	1.921989	13 2 3 9 6 7 11 12	[29,[49,53,25,40,48,67,29,24,45,51],[13,14,15,31,41,42,55,11,12,16],18,[17,18,44,3,38,2,4,7,5,8],17,17,[2,19,32,38,44,56,57,3,17,1]]	56.26993	-70.7691	-13.1984	-48.4544	-7.03906
10	4nyi	1	10	-8.326097	1.6968176	44 3 8 45 4 6	[[11,34,40,3,5,13,15,27,31,36],[36,46,51,53,5,28,32,2,12,24],[24,28,32,33,31,27],10,6,[10,30,39,29,52,9,25,38,23,26]]	84.4431	-114.629	-17.735	-55.639	-7.0261
1	4nyi	1	1	-8.325397	2.0378964	13 14 2 3 8 9 10 4 5	[27,27,[40,46,52,21,22,27,67,23,37,47],[37,41,55,68,69,13,14,31,12,15],[11,16,15,14,13,12],18,18,28,28,[17,18,44,3,2,4,7,38,1,5],[2,17],17,[1,2,19,38,56,3,17,32,44,6]]	76.03182	-79.5981	-11.0356	-55.7851	-7.0254
5	4nyi	1	5	-8.315731	2.3666356	13 2 3 8 9 10 6 7 11	[29,[47,57,61,25,33,56,29,24,53,28],[48,56,13,14,63,11,12,15,16,31],[11,16,15,14,13,12],18,18,[17,18,51,3,45,2,4,7,5,8],17,17,[70,71,43,69,2,19,45,51,52,3],[17,18,51,2,3,4,45,65,32,33]]	29.27787	-56.4189	-14.2501	-53.9141	-7.01573
10	4bib	1	10	-8.313179	0.79843855	19 22	[[24,28,32,33,31,27],9]	82.11705	-161.312	-16.1625	-54.2604	-8.31318
11	4bib	1	11	-8.31276	1.3114437	48 14 33 8 4 5 6	[18,18,18,[4,5,6,21,1,2,3,7,8,9],30,30,[13,14,15,16,31,32,41,51,52,11]]	52.18053	-115.961	-14.2425	-48.8493	-8.31276
2	4nyi	1	2	-8.304516	2.6906543	13 14 2 3 8 9 10 6 7	[29,29,[46,56,60,24,25,29,55,52,57,28],[47,13,14,31,62,11,12,15,16,46],[11,16,15,14,13,12],18,18,[17,18,50,2,3,44,1,4,7,5],17,17,[70,43,71,2,19,44,50,51,3,17],[17,18,50,2,3,4,33,65,69,71]]	62.89388	-78.3473	-12.9069	-49.0506	-7.00452
2	4nyi	1	2	-8.288651	2.4773939	2 6 12 16	[[46,12,33,53,65,8,45,7,9,10,46,12,33,53,65,8,45,7,9,10],[29,55,61,24,30,60,56,57,25,52],[19,25,29,56,60,63,24,28,55,51],[30,54,61,23,29,60,22,24,53,28]]	67.60922	-67.66	-12.7079	-29.4612	-6.98865
2	4nyi	1	2	-8.281833	2.7046778	13 2 3 8 9 10 6 7 11	[29,[46,56,60,25,33,55,24,29,52,58],[47,55,13,14,62,11,12,15,16,31],[11,16,15,14,13,12],18,18,[17,18,50,3,44,2,4,7,5,8],17,17,[68,2,19,44,50,51,64,3,17,1],[17,18,50,2,3,4,33,35,63,64]]	59.77292	-51.4565	-13.9625	-53.6381	-6.98183
10	4nyi	1	10	-8.280778	2.1241579	1 13 2 27 3 45 6 26	[[24,28,32,33,31,27],10,[36,26,49,5,7,23,39,30,10,22],3,[5,34,36,42,9,38,13,15,46,1],12,[12,41,33,11,31,40,27,50,32,24],[4,6]]	87.92629	-74.2485	-16.6624	-81.9981	-6.98078
1	4nyi	1	1	-8.276267	2.6702425	2 3 8 9 6 7 11	[[43,51,26,27,46,52,21,22,50,45],[42,46,27,52,14,15,31,55,11,12],[11,16,15,14,13,12],18,[18,17,44,3,2,4,38,68,7,5],17,17]	56.73802	-91.3042	-11.2344	-51.1815	-6.97627
20	4ehz	1	20	-8.266013	1.1108505	1 2 3 12 13 19 15	[[3,4,5,2,7,17,18,38,1,6],[4,7,8,9,10,5],[4,7,8,9,10,5],31,31,19,29]	53.28429	-129.2	-13.9382	-48.6755	-8.26601
13	3flw	1	13	-8.263854	0.92823684	2	[[38,1,40,66,2,34,67,3,4,5]]	125.953	-73.8181	-11.1754	-37.7056	-8.26385
10	4bib	1	10	-8.261035	1.9780769	7 1 42 15 8 4 5	[[28,51,24,32,53,2,7,8,19,20],[24,28,32,33,31,27],4,[24,28,32,33,31,27],[40,50,42,46,45,5,11,13,15,17],6,6]	88.21059	-143.845	-13.3592	-48.2637	-8.26104
10	4ehz	1	10	-8.259525	1.0929546	45 1 2 21 19 22 20	[[3,4],[45,46,1,16,17,42,4,13,14,15],[7,23,22,21,19,20],[22,25,29,30,26,23],[11,11],[11,11],[40,43,44,3,34,15,1,6,13,14]]	81.98629	-123.244	-17.0593	-43.1252	-8.25953
10	4bib	1	10	-8.242746	1.3935769	27 3 20 21 8 6	[3,[36,5,2,19,44,15,17,20,24,27],9,9,[27,37,48,7,20,24,28,31,32,33],[9,10,22,23,25,26,29,30,38,39]]	87.82349	-137.188	-17.172	-40.6502	-8.24275
10	4nyi	1	10	-8.233611	0.99840832	3 6	[[36,31,33,44,11,12,24,27,28,32],[10,30,39,29,52,25,38,9,22,23]]	79.51627	-113.341	-14.9667	-51.6167	-6.93361
bulit-	4ehz	1	1	-8.224356	0.33132178	1.5364238	1 2 3 4 5 6 7	[[5,14,1	27.02839	-91.0357	-12.9465	-43.3418
5	4nyi	1	5	-8.210027	2.8420458	13 14 2 3 9 6 7 11 1	[29,29,[57,61,25,33,50,29,56,24,53,32],[13,14,15,31,48,49,63,11,12,16],18,[17,18,51,3,45,2,4,7,5,8],17,17,[2,19,45,51,52,1,3,17,6,68]]	25.72868	-59.273	-13.1006	-43.5294	-6.91003
20	4nyi	1	20	-8.190424	1.1481181	2 3 4 6	[[46,5,6,10,21,37,40,1,19,20],[19,20,26,39,40,43,45,46,1,24],28,[14,31,35,51,13,12,15,34,11,16]]	45.66469	-83.9979	-14.4436	-38.6561	-6.89042

11	3v6s	1	11	-8.176206	1.7887602	7 31 14 9 10 5	[31,32,[8,16,32],17,17,[11,16,15,14,13,12]]	50.65619	-92.827	-14.2607	-50.4421	-8.17621
bulit-	4ehz	1	1	-8.173535	0.53262162	1.0754225	8 1 2 3 4 5 9 10 11 6	[[19,2,1,	28.28151	-78.2768	-11.9658	-41.5704
11	3v6s	1	11	-8.171367	1.0150359	31 14 9 10 5	[32,[8,16],17,17,[11,16,15,14,13,12]]	54.61262	-104.578	-13.9947	-49.682	-8.17137
6	3v6s	1	6	-8.157945	1.2419165	9 11	[32,[11,16,15,14,13,12]]	78.27081	-123.066	-16.8948	-44.7972	-8.15794
10	4bib	1	10	-8.146037	1.8997653	26 1 3 22 8	[6,[24,28,32,33,31,27],[2,19,20,24,28,51,32,7,21,27],9,[27,31,50,7,20,24,28,32,33,51]]	81.05023	-116.684	-13.7552	-50.12	-8.14604
6	3v6s	1	6	-8.131212	1.2411218	12 21 29 30	[[20,28,38,51,52,53,26,27,34,13],30,32,32]	81.62798	-93.4995	-12.7137	-49.1144	-8.13121
6	4bib	1	6	-8.129324	1.9202725	7 3 20 21 18 41 42 8	[[35,1,6,18,47,5,10,2,3,4],[12,13,14,38,37,20,10,11,15,16],33,33,[34,34,34,34,34],[34,34,34,34,34],30,[10,12,37,5,6,35,45,9,11,13],33]	88.63881	-117.372	-13.0433	-42.4546	-8.12932
5	3flw	1	5	-8.118611	2.1729813	1 24 13 14	[[52,42,45,51,70,2,3,17,40,41],21,27,27]	43.32836	-83.8803	-12.2879	-39.7497	-8.11861
20	4bib	1	20	-8.104997	1.5776092	1 2 17 9 10 8 4 5 6	[[1,6,5,4,3,2],30,30,28,28,[4,5,44,2,3,6,7,8,9,10],29,29,[12,13,14,15,31,35,36,51,11,16]]	56.55383	-138.114	-14.5242	-46.3431	-8.105
13	3flw	1	13	-8.103448	1.9111835	21 22 13	[32,32,27]	125.4436	-83.3921	-11.7097	-38.5109	-8.10345
2	3flw	1	2	-8.098372	1.9770241	1 13 14	[[53,58,54,61,21,22,23,26,30,57],17,17]	68.62505	-86.5816	-12.3279	-51.8807	-8.09837
11	4ehz	1	11	-8.092423	2.0998445	1 2 3 21 4 26 15 30	[[11,12,13,48,14,15,16,31,35,36],[4,7,8,9,10,5,4,7,8,9,10,5],[4,7,8,9,10,5],[1,6,5,4,3,2],17,18,30,29]	64.57497	-111.1	-14.8499	-48.0173	-8.09242
1	3flw	1	1	-8.086477	2.3936214	1 2 3 4 5 6 7	[[58,32,38,56,57,19,44,1,2,3],[32,57,58,19,20,21,26,27,45,51],[1,6,5,4,3,2],29,29,30,30]	75.28716	-80.5484	-11.301	-45.8466	-8.08648
13	4bib	1	13	-8.084406	1.1363664	35 51 19 22 12 13 8	[[9,43,46,49,55,12,15,16,17,22],24,[1,6,5,4,3,2],33,34,34,[45,1,2,3,4,5,6,34,38,40],[36,62,67,68,35,69,30,31,32,52,36,62,67,68,35,69,30,31,32,52]]	142.084	-82.3831	-11.6323	-35.161	-8.08441
18	3v6s	1	18	-8.071128	1.6776079	1 27 14	[20,20,4]	68.02945	-89.1972	-12.0916	-35.5805	-8.07113
1	3flw	1	1	-8.069157	1.774137	1 6 7	[[65,66,36,60,34,35,61,64,63,69],27,27]	61.79409	-73.6782	-13.0566	-43.0021	-8.06916
11	3v6s	1	11	-8.069071	2.3088777	4 25 35	[29,29,32]	57.79223	-79.9218	-13.4836	-40.2039	-8.06907
10	4ehz	1	10	-8.067395	1.2085631	1 23 46 20 6	[[11,12,31,32,33,41,20,24,27,46],6,6,[41,11,12,27,31,32,33,40,53,24],[21,22,2,7,8,19,20,23,25,26]]	89.99408	-121.498	-14.3627	-44.6269	-8.0674
2	3flw	1	2	-8.06586	2.5413311	1	[[12,13,14,15,16,9,11,31,45,46]]	72.93127	-79.6911	-12.9178	-42.8426	-8.06586
2	3flw	1	2	-8.0621	3.0096555	1	[[33,24,25,28,29,32,34,39,56,60]]	66.78182	-118.755	-12.8304	-36.9061	-8.0621
11	3v6s	1	11	-8.053769	0.83193821	31 5 18 19	[31,[11,16,15,14,13,12],32,32]	58.64365	-118.704	-13.471	-41.9309	-8.05377
11	4nyi	1	11	-8.03745	2.0078197	2 6 7 11	[[7,8,37,18,34,3,4,9,17],[30,43,46,50,24,29,49,23,25,44],29,29]	66.42595	-95.5746	-16.482	-37.7423	-6.73745
11	3v6s	1	11	-8.037385	1.8052697	18 19	[31,31]	63.73562	-84.2018	-13.8947	-43.3846	-8.03739
4	3v6s	1	4	-8.025078	3.4641352		[]	50.78114	-70.8694	-13.255	-46.3903	-8.02508
4	3v6s	1	4	-8.024909	1.4256941	20	17	55.29835	-84.6403	-12.5765	-45.3743	-8.02491
2	3flw	1	2	-8.015816	1.0253828	1 4 13 14	[[7,17,18,3,4,5,8,9,10,45],30,29,29]	75.69312	-115.542	-13.2929	-43.9907	-8.01582
5	3flw	1	5	-7.999114	2.6703446		[]	40.1778	-122.8	-12.3224	-43.4347	-7.99911
11	4ehz	1	11	-7.993818	1.2524844	1 2 19 14	[[20,21,26,27,40,45,47,22,46,23],[1,6,5,4,3,2],30,8]	63.01806	-110.546	-14.0437	-43.2166	-7.99382
20	4bib	1	20	-7.992757	1.2861713	1 2 9 8 6	[[1,6,5,4,3,2],30,29,[4,5,44,1,2,3,6,7,8,9],[12,13,14,15,31,34,35,36,51,11]]	54.97202	-123.753	-13.5488	-42.0621	-7.99276
4	3v6s	1	4	-7.988847	1.214219	1 21 14 5	[31,28,8,[11,16,15,14,13,12]]	57.75245	-107.23	-16.3589	-39.4483	-7.98885
bulit-	4nyi	1	1	-7.988575	0.70956022	2.3975041	1 2 3 4 5 6 7 8 9 10 11 12 13 14 15 16 19 20 21 22 23 24 25 26 27 29 30 31 32 33 34 35 36 37 38	[4,4,4,4,	-864.202	-110.311	-40.0725	-205.563
4	4ehz	1	4	-7.988536	1.1418816	1 26 15 17 6	[[23,40,2,3,19,31,32,39,21,24],29,17,17,[19,39,3,12,4,7,17,32,8]]	52.56095	-135.603	-13.7005	-44.3686	-7.98854
13	4bib	1	13	-7.985225	2.4732723	35 3 6	[[16,17,22,23,44,49,55,15,18,53],[8,9,42,44,10,43,5,7,41,1],[2,3,4,5,34,35,36,39,68,69]]	116.8923	-109.833	-11.896	-36.8341	-7.98522
20	3v6s	1	20	-7.984271	1.9414388	1 27 14 9 10	[29,29,8,17,17]	52.64213	-67.7603	-13.5877	-49.3198	-7.98427
18	3v6s	1	18	-7.980941	2.7284598	1 27 39	[20,20,9]	77.67139	-75.2725	-11.7061	-44.1908	-7.98094
bulit-	4ehz	1	1	-7.980644	3.6672642	3.3211176	8 1 12 9 10	[[34,35,3	27.27276	-67.3174	-12.957	-32.633
4	4ehz	1	4	-7.977727	1.3124586	2 24 25 6 27 14 16	[[11,16,15,14,13,12],[4,7,8,9,10,5],[4,7,8,9,10,5],[17,4,7,8,9,34,3,19,39,2],31,31,31]	49.06264	-106.458	-13.6852	-43.6141	-7.97773

20	4ehz	1	20	-7.974769	1.5015057	1 2 12 13 20 15 30 7	[[3,4,5,7,2,8,17,18,38,1],[4,7,8,9,10,5],31,31,[1,2,3,4,17,32,38,39,7,18],29,27,30]	63.69658	-158.466	-13.8921	-47.0372	-7.97477
10	4ehz	1	10	-7.974434	0.71694803	1 21 4 5 19 22 6 7	[[8,37,47,1,2,18,21,43,44,6],[22,25,29,30,26,23],10,10,[4,4],4,[4,13,42,45,14,16,35,46,1,15],11]	91.16113	-144.95	-14.3293	-43.0168	-7.97443
4	4ehz	1	4	-7.966767	2.5640512	1 2 24 25 28 29 6 14	[[12,13,35,36,9,10,11,5,7,8],[11,16,15,14,13,12],[4,7,8,9,10,5],[4,7,8,9,10,5],28,28,[7,8,17,34,4,5,9,10,38,3],[28,31],[28,31]]	50.97031	-96.5396	-14.5077	-49.7455	-7.96677
bulit-	3v6s	1	1	-7.965797	10.498543	2.6099167	1 2 3 4 5 6 7 8	[48,48,[2	-127.542	-70.216	-12.5911	-51.0016
10	4ehz	1	10	-7.963913	2.1012707	45 47 1 2 20	[[3,4],3,[45,46,1,16,17,42,4,5,13,14],[24,28,32,33,31,27],[34,43,44,15,1,3,13,14,16,17]]	90.25816	-126.234	-13.8137	-46.5988	-7.96391
20	4bib	1	20	-7.96255	1.4704187	1 4 6	[[1,6,5,4,3,2],29,[12,13,14,15,16,31,34,35,36,51]]	54.41158	-107.8	-13.8438	-40.3152	-7.96255
20	4nyi	1	20	-7.958779	2.4635549	42 43 2 8 9 10 6 7 1	[29,29,[34,48,28,42,49,21,23,25,29,22],[11,16,15,14,13,12],18,18,[17,18,38,3,2,4,32,1,7,5],17,17]	58.12151	-65.789	-14.198	-47.6544	-6.65878
11	4nyi	1	11	-7.956529	2.2726536	42 43 6 7 11	[27,27,[31,52,32,51,14,13,15,36,11,12],31,31]	67.31599	-79.3851	-15.56	-32.189	-6.65653
20	4bib	1	20	-7.951141	1.0741042	48 14 33 8 4 5 6	[18,18,18,[4,5,6,21,46,1,2,3,7,8],[27,30],[27,30],[12,13,14,31,35,41,51,11,15,16]]	55.51727	-107.158	-13.9448	-48.7247	-7.95114
20	4ehz	1	20	-7.943485	0.93831062	1 12 19 22 30 49 14	[[45,20,26,27,40,46,47,24,30,43],17,30,30,29,29,8]	67.21497	-129.363	-13.6465	-41.9798	-7.94349
20	4nyi	1	20	-7.940602	2.0095155	3 8 9 10 4 5 6 7 11	[[26,27,35,45,47,13,14,31,51,12],[11,16,15,14,13,12],18,18,21,21,[17,18,38,3,2,4,32,7,5,8],17,17]	56.05698	-70.972	-13.9313	-48.5698	-6.6406
20	3v6s	1	20	-7.939966	1.4997191		[]	59.56714	-86.2235	-13.7687	-34.7502	-7.93997
18	4nyi	1	18	-7.939677	1.7055548	56 39 2 6	[20,22,[30,47,32,8,10,33,19,39,38,14],[51,27,28,3,25,26,1,2,31,4]]	65.33869	-81.5184	-12.8035	-30.0031	-6.63968
20	3v6s	1	20	-7.937837	2.7486799	7 14 9 10	[31,8,17,17]	51.50123	-110.009	-13.6699	-49.6908	-7.93784
6	3flw	1	6	-7.915444	1.4463979	21 22 13 14	[34,34,30,30]	71.70787	-107.746	-11.8233	-43.2489	-7.91544
20	3v6s	1	20	-7.913864	1.5970987	1 27 4 25 5	[29,29,18,18,[11,16,15,14,13,12]]	59.92608	-97.0288	-13.8518	-39.7832	-7.91386
20	3v6s	1	20	-7.913795	2.7075276	14	26	55.88451	-87.89	-15.7418	-33.6232	-7.91379
9	4ehz	1	9	-7.907664	2.2011406	1 26 31 15 17 6	[[33,35,3,4,7,26,29,32,5,30],12,12,23,23,[30,28,44,2,3,4,14,15,46,51]]	24.49206	-84.7625	-11.4974	-40.1542	-7.90766
4	4ehz	1	4	-7.905552	1.4599726	1 2 15 17 30 7 8	[[5,6,10,16,33,38,9,11,15,37],[4,7,8,9,10,5],[28,28],28,31,28,28]	50.94199	-121.626	-15.6181	-50.7942	-7.90555
11	4nyi	1	11	-7.900085	3.5543027	2 6 7 11 15 41	[[48,9,10,11,12,28,35,13,14,15],[33,39,38,2,17,1,3,19,6,40],17,17,18,18]	56.10132	-106.646	-13.7992	-35.0818	-6.60009
1	3flw	1	1	-7.881654	1.6400632	1 8 9 10 11 12 13 14	[[59,69,33,37,60,67,29,24,25,28],18,18,18,18,30,17,17]	73.05812	-134.014	-12.5248	-33.2164	-7.88165
11	4nyi	1	11	-7.87949	2.5750432	2 3 9 10 4 5 6 7 11	[[35,46,21,22,26,27,41,47,45,40],[36,41,42,47,27,13,14,51,11,12],18,18,28,28,[17,18,38,3,33,2,4,7,5,8],17,17]	68.37599	-53.0504	-13.8359	-56.2787	-6.57949
6	4bib	1	6	-7.867219	1.259715	7 3 43 44 39 8	[[13,20,28,38,53,29,30,34,52,55],[46,35,44,37,9,10,11,5,6,8],33,33,[25,31,56,57,59,24,26,27,32,33],[50,11,36,7,8,9,12,13,14,15]]	84.45984	-124.156	-12.6848	-30.9863	-7.86722
4	4bib	1	4	-7.859421	1.5311718	7 1 3 22 12 13 14 25	[[35,9,10,11,12,8,13,14,15,16],[4,7,8,9,10,5],[1,6,33,44,2,5,18,32,3,4],28,19,19,17,[11,16,15,14,13,12],[1,2,3,4,5,6,7,8,9,10]]	54.54079	-90.7188	-13.9988	-42.9192	-7.85942
4	3v6s	1	4	-7.837198	1.3561974	22 23 14 24 4 25 26	[[23,28,31],[23,31],19,19,17,17,[4,7,8,9,10,5]]	57.56427	-96.9943	-13.578	-49.9585	-7.8372
20	4bib	1	20	-7.834794	1.5451279	7 1 9 10 8 23 6 16	[[39,19,32,1,21,2,6,20,28,41],[1,6,5,4,3,2],29,29,[4,5,44,2,3,6,7,8,9,10],[11,16,15,14,13,12],[12,13,14,15,31,34,35,51,11,16],[11,16,15,14,13,12]]	56.90289	-124.475	-14.0456	-48.8234	-7.83479
4	4ehz	1	4	-7.833155	1.2116785	1 9 28 15 17	[[10,11,15,16,33,38,12,13,14,37],[23,18,33,40,1,6,5,10,21,24],31,29,29]	61.71643	-110.624	-14.1827	-45.2218	-7.83315
5	4nyi	1	5	-7.813145	3.6437624	2 3 8 6 12 16	[[47,33,25,28,32,34,57,61,64,37],[48,57,61,29,12,13,14,56,63,11],[11,16,15,14,13,12],[17,18,51,2,3,45,4,7,8,1],[70,45,51,69,2,17,19,52,1,3],[17,18,51,3,69,45,39,44,38,40]]	28.10895	-72.7529	-15.1555	-56.676	-6.51315

6	4bib	1	6	-7.81149	2.5946774	7 1 3 19 22 38 20 21	[[1,2,3,4,5,6,47,49,10,35],[4,7,8,9,10,5],[50,12,13,37,38,24,9,11,14,15],[11,16,15,14,13,12],34,34,33,33,[41,3,17,19,42,2,21,43,1,4],[11,16,40,8,9,12,13,14,15,36],34,[20,25,26,27,28,31,32,51,52,53]]	77.31652	-92.0979	-12.5547	-39.2355	-7.81149	
18	4nyi	1	18	-7.809878	1.9131055	6	[[28,2,25,26,1,3,9,31,4,5]]	70.77139	-82.2354	-13.1235	-30.5478	-6.50988	
10	3flw	1	10	-7.804618	1.6571481		[]	82.36637	-96.5194	-13.249	-36.4628	-7.80462	
4	4bib	1	4	-7.799832	2.8766658	26 7 1 3 8	[24,[44,24,31,32,1,2,6,18,46,21],[1,6,5,4,3,2],[38,5,7,8,9,10,4,6,11,16],[4,5,3,6,7,8,9,10,17,19]]	47.36208	-125.287	-12.5686	-43.6511	-7.79983	
11	4bib	1	11	-7.788797	0.65250391	1 27 28 3 19 9 8 6	[[1,6,5,4,3,2],27,27,[10,37,5,40,7,8,9,34,18,4],[4,7,8,9,10,5],29,[28,3,4,5,7,8,9,17,18,34],[12,13,14,15,31,36,51,11,16,35]]	57.04365	-126.685	-14.1648	-47.4985	-7.7888	
4	3v6s	1	4	-7.787243	3.4950352	22 23 14 24 4 25 26	[[23,28,31],[23,31],19,19,17,17,[4,7,8,9,10,5]]	57.40222	-84.2553	-13.2854	-49.8712	-7.78724	
18	3v6s	1	18	-7.765204	1.5747656	28 42 43 44 22 23 14	[21,21,24,24,26,26,27,27]	74.37094	-74.0401	-12.427	-35.7787	-7.7652	
10	3flw	1	10	-7.764375	1.5997072	8 9 30 19 20 13 14	[8,8,6,12,12,5,5]	84.69486	-97.6654	-13.2567	-38.5681	-7.76437	
18	3v6s	1	18	-7.760786	1.4158045	43 44 14 45 32 33	[24,24,4,22,27,27]	62.55706	-86.4166	-12.7559	-32.3538	-7.76079	
20	4nyi	1	20	-7.759458	1.656143	42 3 9 10 4 6 7 11	[27,[26,27,45,47,14,15,31,36,51,13],18,18,21,[17,18,38,2,3,32,4,7,5,8],17,17]	52.81696	-76.16	-16.0551	-43.6302	-6.45946	
4	4bib	1	4	-7.734084	1.517929	27 28 3 19 9 10 6 29	[30,30,[1,2,3,4,5,32,6,18,19,39],[4,7,8,9,10,5],31,31,[9,10,11,12,13,14,15,16,35,36],[11,16,15,14,13,12],[11,16,15,14,13,12]]	60.49876	-134.31	-14.2374	-35.3706	-7.73408	
9	3flw	1	9	-7.732213	1.6698945	13	23	25.40448	-73.1309	-10.5594	-31.7357	-7.73221	
20	4ehz	1	20	-7.728078	0.69821823	1 21 4 5 15 6 7	[[3,4,5,7,1,2,6,8,9,10],[11,16,15,14,13,12],31,31,28,[44,3,4,5,6,7,10,25,28,1],29]	51.78872	-130.593	-14.6973	-41.9089	-7.72808	
11	4bib	1	11	-7.725598	1.5582056	1 2 17 27 9 10 8 23	[[1,6,5,4,3,2],30,30,29,27,27,[4,5,27,47,1,2,3,6,7,8],[11,16,15,14,13,12],[12,13,14,15,31,35,36,51,11,16],[11,16,15,14,13,12]]	54.25476	-99.1033	-14.469	-48.0127	-7.7256	
9	3flw	1	9	-7.719839	2.6549652		[]	15.50808	-88.4998	-10.0844	-33.3296	-7.71984	
6	4nyi	1	6	-7.700866	1.816487	20 8 29 30 31 32 6 2	[[37,10,12,38,13,29,30,54,55,34],[8,8,8,8,8,8,11,16,15,14,13,12],[13,14,15,20,24,50,59,12,29,38],32,32,32,[45,46,22,18,43,2,21,42,1,6],20]	94.57903	-87.7077	-15.1826	-43.0141	-6.40087	
13	4nyi	1	13	-7.689921	2.1113589	1 20 8 46 47 29 48 3	[[1,6,5,4,3,2],[14,45,48,51,7,11,13,20,41,47],[15,20,19,18,17,16],33,33,[15,16,17,18,19,20,52,65,13,21],29,29,29,34,8,8,[2,34,66,3,35,67,36,69,68,1],[1,6,5,4,3,2],37]	126.5406	-43.9685	-13.885	-67.534	-6.38992	
11	4nyi	1	11	-7.667254	1.3562924	2 9 4 5 6 7 11	[[45,21,26,41,22,46,27,50,47,40],18,28,28,[17,18,38,3,33,2,4,7,5,8],17,17]	48.9347	-92.8303	-14.2879	-47.4593	-6.36725	
4	4bib	1	4	-7.651601	0.91229284	7 3 31 32 8 6	[[21,27,30,40,47,2,32,1,3,18],[30,50,27,47,5,6,10,29,33,49],28,28,[44,24,25,31,45,46,51,6,18,33],[11,12,13,14,15,16,20,37,38,9]]	56.33858	-90.3706	-13.3866	-29.0405	-7.6516	
6	3flw	1	6	-7.650574	1.5236218	21 13 14	[33,[30,34],[30,34]]	81.46258	-90.9057	-11.4952	-49.6717	-7.65057	
bulit-	3v6s	1	1	-7.649303	5.237699	1.7463188	9 10 11 12 13 14 15 16 17	[31,31,[1	-118.008	-60.3689	-13.1661	-47.5615	
11	4ehz	1	11	-7.641799	1.5593772	1 48 6 14	[[45,20,21,22,26,40,46,27,47,23],29,[29,49,52,32,37,15,16,24,25,28],8]	61.85114	-118.023	-14.3148	-42.6629	-7.6418	
9	3flw	1	9	-7.641583	1.4404519	12	23	12.27426	-77.6212	-9.90023	-32.7566	-7.64158	
4	4bib	1	4	-7.635033	1.9196689	7 1 20 14 33 34 8 23	[[46,1,2,18,32,6,26,30,33,50],[1,6,5,4,3,2,4,7,8,9,10,5],22,17,17,23,[5,10,4,7,8,9,11,12,17,34],[11,16,15,14,13,12]]	48.16768	-139.744	-13.9669	-47.2768	-7.63503	
1	3flw	1	1	-7.627237	2.6845577	1 13	[[32,56,57,19,49,58,48,20,24,25],16]	70.13213	-132.497	-12.2476	-42.0021	-7.62724	
11	4ehz	1	11	-7.624363	1.6358069	1 2 3 26 19 22 30 49	[[10,12,13,35,36,40,9,11,14,20],[4,7,8,9,10,5,4,7,8,9,10,5],[4,7,8,9,10,5],18,27,27,28,28]	63.0318	-162.185	-14.2858	-53.8342	-7.62436	
18	3flw	1	18	-7.61911	1.3421594	10 13 14	[20,9,9]	68.23901	-103.653	-11.1566	-36.3107	-7.61911	
1	3flw	1	1	-7.615534	1.3369225	15 16 17 12 18	[[11,16,15,14,13,12],27,27,30,30]	72.08506	-120.244	-11.6209	-31.3477	-7.61553	
18	3flw	1	18	-7.613781	2.5939767	8 10 11 16 13 14	[24,24,24,9,24,24]	66.84522	-82.7135	-11.0569	-43.1477	-7.61378	

9	4ehz	1	9	-7.61334	1.5297505	1 15 17 6	[[31,36,37,3,4,7,26,29,41,5],23,23,[30,28,46,2,3,4,5,7,14,32]]	10.81003	-114.075	-11.503	-40.0809	-7.61334
18	4ehz	1	18	-7.60355	1.9817828	45 47 1 7 8 10 11	[[26,26],26,[3,34,2,4,25,26,27,28,29,51],20,20,24,24]	66.77148	-86.6525	-12.4926	-46.7507	-7.60355
9	4bib	1	9	-7.59874	2.186125	26 31 32 46	[23,22,22,12]	15.82112	-67.029	-10.2721	-40.5716	-7.59874
4	3flw	1	4	-7.597671	0.99726355	19	28	53.17871	-90.8776	-11.9509	-43.7169	-7.59767
10	3flw	1	10	-7.568221	1.617691	31 32 21 4 5 6	[11,11,10,6,6,12]	82.48872	-120.363	-13.9411	-47.2635	-7.56822
6	3flw	1	6	-7.566315	4.2589626	26 27	[34,34]	73.8251	-77.8892	-11.61	-42.6177	-7.56632
10	3flw	1	10	-7.557315	1.2317564	8 9 28	[8,8,6]	85.76409	-115.187	-14.1276	-35.9174	-7.55731
10	3flw	1	10	-7.554987	1.2626179	31 21 6 7	[11,10,12,12]	82.03736	-117.75	-12.8695	-46.0193	-7.55499
13	4nyi	1	13	-7.547963	1.9053205	13 14 2 6 12	[31,31,[13,59,63,14,48,31,27,28,58],[54,55,22,23,53,17,37,60,16,18],[24,29,60,22,54,21,37,56,25,17]]	133.2347	-107.324	-13.8675	-25.5369	-6.24796
6	4bib	1	6	-7.547572	1.3846476	8 6	[[51,52,53,56,13,20,26,38,14,24],[38,35,44,46,22,45,6,10,12,37]]	74.57629	-92.4195	-12.6212	-36.3464	-7.54757
18	3v6s	1	18	-7.547502	2.5174949	15 9 10	[[1,6,5,4,3,2],9,9]	65.41985	-68.1621	-11.7777	-41.6564	-7.5475
18	4ehz	1	18	-7.544591	2.5908349	45 47 1 7	[26,26,[3,2,4,25,26,27,28,29,1,6],20]	69.34658	-92.3045	-12.2306	-46.8158	-7.54459
9	3flw	1	9	-7.541399	1.6534474	10 11	[23,23]	18.20354	-90.6983	-10.0083	-34.667	-7.5414
9	4ehz	1	9	-7.540765	2.1552973	1 40 41 15 17 6	[[33,37,4,7,29,31,34,3,5,32],24,24,23,23,[30,2,28,46,3,4,14,15,29,32]]	27.47306	-84.1539	-13.6038	-39.6811	-7.54077
11	4bib	1	11	-7.534414	1.3651794	7 3 42 31 32 8 4 5 6	[[8,9,10,11,16,37,4,5,7,12],[40,43,45,47,37,32,52,26,15,16],30,31,31,[28,29,44,49,25,11,14,15,16,32],28,28,[22,23,30,41,42,48,50,24,28,29]]	60.24745	-116.23	-13.9728	-40.4702	-7.53441
18	4ehz	1	18	-7.514754	1.8007246	1 6 10 11	[[38,12,14,30,33,40,11,13,37,39],[20,41,42,16,6,7,8,15,32,48],9,9]	69.07843	-104.94	-12.9817	-40.3253	-7.51475
11	4ehz	1	11	-7.500526	0.73904103	1 9 21 4 26 31 7 8	[[47,12,13,27,35,36,14,31,51,26],[19,40,6,20,27,43,47,1,2,5],[1,6,5,4,3,2],17,18,18,29,29]	67.83548	-143.902	-14.1865	-37.9348	-7.50053
18	4bib	1	18	-7.498754	1.6555457	20 8 4 5 6	[27,[10,32,39,40,33,11,12,13,14,34],24,24,[1,2,3,4,5,6,25,26,27,28]]	68.01769	-86.2399	-11.6169	-39.0811	-7.49875
13	4nyi	1	13	-7.490097	1.8827361	49 2 6 12 15 41	[34,[46,49,55,15,16,17,52,63,12,13],[29,60,50,19,18,37,21,20,51,15],[29,57,60,21,24,28,37,26,27,58],31,31]	136.3447	-95.1692	-15.474	-33.0251	-6.1901
4	3flw	1	4	-7.472914	1.1331477	1 12 18 4	[[1,2,3,4,5,6,19,32,39,18],17,17,31]	72.014	-108.52	-11.9675	-39.3528	-7.47291
18	4ehz	1	18	-7.44558	1.8101441	1 26 31 20 15 30 49	[[6,11,12,13,37,38,14,33,34,35],26,26,[39,36,32,11,12,13,14,40,10,34],21,22,22,21,21]	68.12923	-104.063	-12.2688	-37.1774	-7.44558
18	4ehz	1	18	-7.426246	2.6387355	45 1 19 14	[26,[3,4,5,30,32,1,2,25,27,28],9,21]	69.62457	-112.482	-12.3704	-34.5367	-7.42625
4	3flw	1	4	-7.422835	3.0004547	1	[[30,1,6,18,26,27,33,46,50,21]]	47.19243	-116.058	-12.8522	-40.2831	-7.42283
18	4bib	1	18	-7.414228	1.5143226	7 9 10 8	[[11,33,34,35,5,6,7,8,10,30],[20,22],[20,22],[39,14,38,40,4,20,32,41,45,1]]	77.14546	-134.346	-12.9525	-35.6622	-7.41423
9	4bib	1	9	-7.411382	1.2685429	7 3 22 38 31 32 8 6	[[24,28,47,51,54,20,2,16,21,15],[27,1,5,28,34,46,2,26,47,6],12,12,25,25,[17,48,49,13,41,42,43,53,18,26],[9,10,11,12,36,37,39,42,8,13]]	20.56191	-76.9512	-11.5801	-32.9088	-7.41138
18	3flw	1	18	-7.41075	1.6439043	16 17 13 14	[24,24,27,27]	78.62019	-88.4073	-11.0591	-33.2001	-7.41075
13	4nyi	1	13	-7.409832	2.214066	36 50 51 33 4 5 26	[29,29,33,33,8,8,24]	149.8234	-105.318	-14.0982	1.286812	-6.10983
9	4ehz	1	9	-7.370506	1.9491693	1 28 15 17 6 14 16	[[8,34,35,41,12,5,13,27,31,4],24,23,23,[30,32,33,7,3,4,28,46,2,5],22,22]	18.10405	-96.9817	-11.4094	-45.6751	-7.37051
18	4nyi	1	18	-7.369726	1.9237374	13 14	[27,27]	77.67364	-74.6476	-13.463	-32.4175	-6.06973
13	4nyi	1	13	-7.369397	2.027149	19 52 23 24 2 3 33 3	[33,33,31,31,[53,45,48,11,14,49,54,13,16,47],[13,16,17,22,49,53,15,18,19,20],33,33,[34,66,67,36,2,1,3,38,69,35],[1,34],34,24]	133.288	-111.725	-13.511	13.96621	-6.0694
9	3flw	1	9	-7.360005	1.7871805	12 18	[22,22]	13.51961	-77.2342	-10.4089	-26.5635	-7.36001
18	4nyi	1	18	-7.355108	2.7502472	21 3 6 26	[19,[33,41,42,48,50,15,16,22,47,7],[51,27,28,25,26,2,31,1,3,9],21]	75.47659	-110.177	-12.9478	-47.4633	-6.05511
20	4ehz	1	20	-7.354519	1.0703515	52 53 1 4 5 14 16	[29,29,[15,16,28,36,37,48,49,14,24,25],17,17,19,19]	77.21075	-105.076	-13.6562	-39.8372	-7.35452

9	4ehz	1	9	-7.339539	2.1422143	1 33 15 6	[[9,36,37,26,29,31,41,3,4,5],[17,18,19,23,48,49,53,55,25,20],[23,23],[30,32,7,28,46,2,3,4,5,6]]	11.97346	-77.4047	-11.3826	-39.7031	-7.33954
bulit-	3flw	1	1	-7.338225	2.447758	1.3939288	1 2 3	[34,34,3	-113.776	-113.473	-12.8995	-47.7071
9	3v6s	1	9	-7.332757	0.94712698	12 29 30	[[24,26,43,47,54,20,13,42,1,27],23,23]	10.85174	-81.5693	-10.7041	-36.6066	-7.33276
9	3v6s	1	9	-7.326075	1.1869534	22 23 7 8	[[22,24,25],[24,25],25,25]	24.69187	-67.7802	-11.1562	-39.6796	-7.32607
4	4nyi	1	4	-7.319136	1.5627285	19 20 8 4 5 6	[17,[33,6,1,5,10,18,16,38,11,15],[4,7,8,9,10,5],23,23,[47,49,28,29,26,48,25,27,40,21]]	58.33303	-88.6334	-13.8784	1.689981	-6.01914
9	3v6s	1	9	-7.307204	2.1946156	22 23	[24,24]	26.10707	-65.3157	-11.1106	-28.2439	-7.3072
18	4bib	1	18	-7.303286	1.7873173	7 4 5 6	[[4,29,30,32,3,25,27,51,5,6],[17,24],24,[24,50,23,48,43,17,18,21,44,16]]	63.45276	-96.0333	-11.8436	-39.766	-7.30329
4	4nyi	1	4	-7.301714	2.8914843	21 22 23 24 25 3 6 2	[31,31,31,31,31,[1,2,6,18,33,47,50,5,21,27],[22,42,43,20,41,13,14,36,12,15],29]	76.86633	-90.8844	-16.0235	-25.3105	-6.00171
9	3v6s	1	9	-7.301097	1.1053371	22 23 7 8	[[24,25],[24,25],[24,25],25]	24.91677	-68.4289	-11.5731	-39.2737	-7.3011
12	4bib	1	12	-7.293335	1.0106347	3 19	[[4,7,11,27,5,6,10,1,2,3],[1,6,5,4,3,2,4,7,8,9,10,5,4,7,8,9,10,5]]	13.85447	-102.737	-12.1511	-38.8709	-7.29334
18	4nyi	1	18	-7.290773	1.6974003	1 39 2 3 6 15 41	[[1,6,5,4,3,2],22,[30,47,32,4,5,6,8,10,33,38],[14,18,19,21,30,32,38,39,44,46],[28,31,51,9,1,2,3,6,7,23],26,26]	64.69073	-83.9593	-12.674	-34.3413	-5.99077
16	4nyi	1	16	-7.280898	2.0143878	6	[[13,14,22,26,32,15,21,31,11,12]]	10.11772	-112.376	-17.1958	-17.2736	-5.9809
bulit-	4bib	1	1	-7.275651	0.69294822	1.5219957	1 2 3 4 5 6 7 8 9 10	[1,1,5,5,	-31.4761	-93.5227	-10.744	-46.1044
bulit-	3v6s	1	1	-7.248328	9.1660604	1.5671083	3 18 19 20 21 22 23 24 13	[[21,26,2	-130.511	-112.98	-12.9602	-49.2795
7	4nyi	1	7	-7.222248	1.2798332	37 38 6	[21,21,[23,35,36,12,33,37,1,13,22,32]]	15.05696	-71.0541	-14.2238	-48.1383	-5.92225
16	4nyi	1	16	-7.211726	1.0627559	6 17 18 7 11	[[19,29,3,17,2,4,23,7,8,20],[4,7,8,9,10,5],[4,7,8,9,10,5],19,19]	7.320876	-98.9756	-16.8622	-36.2179	-5.91173
9	4bib	1	9	-7.21146	2.4403858	22 38 31 8 6	[12,12,24,[48,49,18,17,19,22,25,55,28,30],[9,10,11,12,36,37,38,39,40,8]]	20.69895	-81.9481	-10.2088	-28.4129	-7.21146
9	3v6s	1	9	-7.203577	2.1556835	31 32 33	[25,22,22]	28.89206	-79.974	-10.8443	-15.318	-7.20358
bulit-	3flw	1	1	-7.202297	2.5099208	4.0048423	1 2 3	[34,34,3	-113.953	-104.421	-11.4017	-46.1287
18	4bib	1	18	-7.186418	1.5134771	1 3 4 5 6	[[1,6,5,4,3,2],[32,38,4,29,3,5,10,11,12,25],[24,24],24,[12,13,35,36,37,40,11,14,34,39]]	59.64215	-120.917	-14.5492	-43.0583	-7.18642
18	4bib	1	18	-7.175961	1.6364166	1 3 42 14 8 4 5 46 5	[[1,6,5,4,3,2],[48,7,23,42,43,49,6,33,1,9],20,26,[30,34,1,2,3,4,5,6,9,25],[21,22],21,22,22]	69.71748	-111.421	-11.7699	-41.7665	-7.17596
16	4nyi	1	16	-7.126083	2.3790083	4 6 7 11	[17,[18,28,1,2,23,6,3,19,29,4],18,18]	3.945181	-103.392	-16.4713	-52.5766	-5.82608
16	4nyi	1	16	-7.122669	2.3456359	6 7 11	[[21,22,14,15,31,32,13,26,12,11],22,22]	15.96304	-109.96	-18.0583	-28.8694	-5.82267
16	4nyi	1	16	-7.097168	0.85867751	27 8 6 7	[21,[11,16,15,14,13,12],[18,28,1,23,2,3,29,6,24,4],18]	7.947134	-100.79	-16.0907	-57.0799	-5.79717
6	4nyi	1	6	-7.095857	1.7487833	21 22 23 2 30 33 34	[34,34,34,[5,6,10,35,46,1,18,22,44,45],25,32,32,[19,41,3,42,7,17,4,8,2,5],[42,21,43,47,48,19,23,1,2,3],[25,33]]	94.43343	-98.842	-14.8118	-13.4393	-5.79586
9	4bib	1	9	-7.092365	1.5634996	26 9 8 6	[23,24,[1,13,26,27,41,42,6,43,45,2],[5,8,9,10,11,12,33,34,37,39]]	29.99367	-81.1397	-10.7942	-29.8351	-7.09237
18	3flw	1	18	-7.085617	1.8999556	10 11 13 14	[22,22,[9,22],[9,22]]	63.53079	-105.858	-12.1831	-41.8955	-7.08562
bulit-	4bib	1	1	-7.079152	0.20755692	1.3589329	11 1 2 3 4 7 8 9 10	[[6,36,3	-25.3719	-81.4902	-11.1028	-44.476
7	4bib	1	7	-7.071707	1.3127925	1 11 8	[[15,20,19,18,17,16],[15,20,19,18,17,16],[20,9,10,15,18,19,24,29,30,16]]	8.807282	-82.0535	-11.814	-38.7148	-7.07171
bulit-	3flw	1	1	-7.071405	2.6210554	1.4495625	4 1 2 3 5	[[8,42,7,	-109.873	-106.06	-11.624	-44.4177
4	3flw	1	4	-7.061531	1.6744499	21 22 16 17 13 14	[29,29,19,19,30,30]	47.72224	-107.812	-13.9255	-40.3053	-7.06153
7	4ehz	1	7	-7.05411	0.95562351	1 9 14	[[1,6,23,24,35,12,36,37,5,10],[30,20,29,4,7,8,9,10,25,5],8]	11.30394	-96.6944	-12.344	-35.9856	-7.05411
7	4bib	1	7	-7.054088	1.5468639	3 19 20 21 8	[[4,3,7,11,26,5,6,10,1,2],[1,6,5,4,3,2],21,21,[34,36,1,2,3,4,5,6,10,12]]	8.525458	-81.3229	-12.3091	-40.2089	-7.05409
9	4bib	1	9	-7.054006	1.4164474	47 3 8	[17,[2,3,14,28,30,45,46,15,44,1],[1,13,26,27,41,42,53,6,43,2]]	28.75077	-113.142	-11.0442	-25.894	-7.05401
bulit-	3v6s	1	1	-7.047496	11.637961	2.3420315	25 26 19 27	[[21,26,2	-126.624	-102.783	-11.7467	-48.6035

16	4bib	1	16	-7.044807	1.8155588	19 20 21	[[1,6,5,4,3,2,4,7,8,9,10,5],22,22]	3.189635	-95.2867	-13.4327	-37.6552	-7.04481
12	4bib	1	12	-7.041366	1.1247075	3 19 8	[[4,7,8,11,5,6,9,10,1,3],[4,7,8,9,10,5,4,7,8,9,10,5],[22,32,34,1,2,3,4,5,6,10]]	13.15589	-92.3168	-12.1136	-36.677	-7.04137
4	4nyi	1	4	-7.018144	1.3267269	6 7 11	[[31,44,51,24,25,45,23,28,48,46],[28,31],[28,31]]	73.40884	-84.6643	-15.3502	-17.2704	-5.71814
bulit-	4bib	1	1	-7.013778	1.2381576	1.2838759	12 3 4 7 8 9 10	[32,5,5,2	-16.7384	-84.6347	-10.9581	-38.537
12	4nyi	1	12	-7.012736	2.5506713	1 6 17 18 26	[[15,20,19,18,17,16],[21,23,35,36,18,29,37,17,19,16],[15,20,19,18,17,16],[15,20,19,18,17,16],13]	40.07943	-72.9525	-13.8015	-36.5315	-5.71274
7	4nyi	1	7	-7.001719	1.4653922	6	[[12,23,35,36,32,22,37,13,33,34]]	11.63471	-81.8782	-14.1144	-48.6875	-5.70172
4	3flw	1	4	-6.996932	2.6677773	1 19 20 23	[[44,31,18,23,24,33,51,25,45,46],28,28,29]	52.13352	-98.9522	-11.917	-42.1563	-6.99693
bulit-	4bib	1	1	-6.991616	1.1935441	1.3202853	12 3 4 7 8 9 10 13	[32,5,5,2	-20.3997	-79.015	-11.3925	-37.8715
12	4bib	1	12	-6.989906	1.2082558	1 8	[[1,6,5,4,3,2],[4,5,2,3,6,7,8,9,10,11]]	11.75577	-102.839	-14.2118	-36.8022	-6.98991
7	4ehz	1	7	-6.978271	1.7569777	24 6	[[4,7,8,9,10,5],[7,8,9,15,25,30,19,20,29,17]]	10.02353	-91.976	-12.852	-37.7522	-6.97827
14	4nyi	1	14	-6.969264	0.77638149	6 7 11 15 41	[[9,23,20,1,2,18,6,7,8,5],9,9,16,16]	-14.2312	-76.0995	-12.0758	-31.5209	-5.66926
3	4nyi	1	3	-6.967546	1.322026	9 6 7 11	[11,[11,13,26,3,2,4,22,7,5,8],13,13]	-11.5928	-93.5671	-13.177	-33.5809	-5.66755
18	3flw	1	18	-6.96307	2.0111325	31 32 13 14	[22,22,[9,9],9]	62.00391	-89.8018	-11.6276	-36.4353	-6.96307
11	3flw	1	11	-6.954475	2.6821859	26 27 13	[19,19,16]	56.59056	-103.25	-14.0921	-39.8474	-6.95447
6	4nyi	1	6	-6.947601	1.3753284	21 22 23 24 2 30 6 1	[34,34,34,34,[37,4,5,7,8,9,10,17,36,11],32,[22,44,45,46,18,35,1,6,10,16],[46,18,22,44,49,45,23,1,2,6],[25,33]]	83.52397	-101.662	-17.8913	-19.2459	-5.6476
7	4bib	1	7	-6.946283	0.97467369	3 19 8	[[4,7,8,11,3,5,9,10,14,25],[4,7,8,9,10,5],[34,32,3,4,5,7,8,11,14,26]]	9.298896	-86.264	-12.2453	-35.9896	-6.94628
12	4bib	1	12	-6.943679	1.3681408	3 19 42 8 16	[[10,31,5,6,24,4,7,8,9,1],[1,6,5,4,3,2,4,7,8,9,10,5],8,[34,22,1,2,3,4,5,6,7,8],[15,20,19,18,17,16]]	11.03218	-94.9717	-13.3629	-40.8919	-6.94368
bulit-	4nyi	1	1	-6.938195	1.2085036	1.7820457	1 3 4 7 8 11 12 13 14 39 19 20 22 23 24 25 40 41 29 30 31 32 33 42 34 35 36 37 38	[3,3,3,3,	-874.106	-118.179	-34.329	-185.684
11	3flw	1	11	-6.929007	2.8312247	12 18	[18,18]	49.00303	-78.9151	-13.3942	-39.9229	-6.92901
7	4nyi	1	7	-6.91467	1.0671321	8 4 6	[[15,20,19,18,17,16],11,[23,35,36,37,12,13,34,2,22,26]]	10.97449	-71.471	-14.3018	-41.7638	-5.61467
bulit-	3v6s	1	1	-6.907033	10.803727	2.7105193	28 9 10 29 30	[[63,54,5	-127.202	-74.5569	-11.7721	-48.535
11	3flw	1	11	-6.904049	4.3869524	19 20 26 27	[30,30,19,19]	49.0776	-71.926	-13.1556	-37.5087	-6.90405
7	4bib	1	7	-6.890504	1.1487869	1 3 20 21 8 23	[[1,6,5,4,3,2],[30,4,5,8,9,10,3,6,7,11],21,21,[34,5,6,1,2,3,4,7,8,9],[15,20,19,18,17,16]]	7.483145	-89.342	-12.3221	-40.9762	-6.8905
12	3v6s	1	12	-6.890323	1.7035582	14 9 10	[8,14,14]	14.83209	-81.2252	-13.0243	-43.8492	-6.89032
16	4bib	1	16	-6.882279	1.3357782	3 19 20 21 8 4	[[7,17,4,8,5,6,9,10,1,2],[1,6,5,4,3,2,4,7,8,9,10,5],22,22,[1,2,3,4,5,6,10,18,23,24],20]	5.60739	-99.6514	-14.4311	-39.6206	-6.88228
12	3flw	1	12	-6.874388	1.014526	1 8	[[32,34,22,33,1,12,26,2,6,13],11]	13.62946	-89.3891	-11.2463	-34.2729	-6.87439
12	3v6s	1	12	-6.870394	1.5246671	17 5	[[4,7,8,9,10,5],[15,20,19,18,17,16,15,20,19,18,17,16]]	14.00432	-89.8514	-13.1449	-36.8369	-6.87039
bulit-	3flw	1	1	-6.869509	8.9612551	1.5927527	6 7	[48,48]	-121.975	-122.543	-11.594	-41.4498
6	4nyi	1	6	-6.863252	1.5915426	23 24 25 35 36 2 6 1	[34,34,34,34,30,[40,35,46,5,6,10,16,22,44,1],[17,41,19,7,36,8,37,3,4,9],[19,41,47,23,48,2,3,4,17,42],[25,33]]	87.14661	-127.815	-14.4044	-7.60999	-5.56325
7	4bib	1	7	-6.860809	1.6143731	1 3 8 23 16	[[1,6,5,4,3,2],[30,5,8,9,10,3,4,6,7,11],[4,5,2,3,6,7,8,9,10,11],[15,20,19,18,17,16],[15,20,19,18,17,16]]	12.07526	-111.694	-12.3468	-41.4543	-6.86081
12	4bib	1	12	-6.85711	1.2764348	1 3 19 42 8 16	[[1,6,5,4,3,2],[10,31,5,6,24,4,7,8,9,1],[1,6,5,4,3,2,4,7,8,9,10,5],8,[34,1,2,3,4,5,6,7,8,10],[15,20,19,18,17,16]]	12.60965	-104.024	-12.7414	-38.5474	-6.85711
12	3flw	1	12	-6.854927	0.78628129	1 8 9 30 33 10 11	[[37,17,18,21,23,29,36,35,16,19],11,11,12,12,11,11]	12.81734	-81.2243	-11.1144	-36.2887	-6.85493
bulit-	4ehz	1	1	-6.854191	3.4221845	1.0153552	8 13 4 5 9 6	[[30,33,3	30.06241	-67.356	-11.6822	-31.2776
14	4nyi	1	14	-6.851021	1.6850591	1 2 6 7 11	[[1,6,5,4,3,2],[27,26,11,13,20,12,29,8,22,21],[16,15,18,2,3,17,1,23,4,19],9,9]	-15.2375	-65.5545	-12.5749	-33.1289	-5.55102
bulit-	3flw	1	1	-6.846555	1.7062606	2.6162446	4 5 8	[[47,12,4	-109.872	-85.045	-10.9911	-46.3782

6	4nyi	1	6	-6.841009	1.0171765	21 22 23 24 2 8 33 3	[34,34,34,34,[35,5,6,10,40,46,18,22,45,1],[11,16,15,14,13,12],32,32,[17,41,19,36,7,8,3,4,9,37],[19,41,47,23,2,3,4,17,42,21],[25,33]]	83.33353	-129.196	-16.8486	-11.8436	-5.54101
7	3v6s	1	7	-6.832715	1.3374072	17 22 23 14	[[15,20,19,18,17,16],11,11,8]	10.10141	-68.9432	-12.8863	-27.95	-6.83272
7	4ehz	1	7	-6.820994	1.1135404	1 24 9 6	[[4,7,11,3,8,14,25,2,26,1],[4,7,8,9,10,5],[6,24,37,5,10,1,12,23,2,35],[7,8,9,10,11,15,25,30,19,20]]	11.33071	-88.3383	-12.2128	-39.0139	-6.82099
9	4nyi	1	9	-6.814036	1.3523568	6 26	[[12,38,10,11,35,40,7,8,32,33],[22,25]]	20.5639	-62.8599	-20.6755	-59.9978	-5.51404
11	3flw	1	11	-6.812326	2.1123097	10 12 18 13 14	[32,18,18,32,32]	47.58169	-101.588	-14.792	-46.4393	-6.81233
11	3flw	1	11	-6.792308	0.97491032	1 3 6 7 13 14	[[2,3,4,7,17,18,33,38,1,5],[4,7,8,9,10,5,4,7,8,9,10,5],30,30,32,32]	56.08928	-111.638	-13.7253	-48.3703	-6.79231
12	3v6s	1	12	-6.786249	0.79053146	17 14 5	[[4,7,8,9,10,5],[8,16],[15,20,19,18,17,16]]	17.6369	-84.4846	-12.6263	-43.3604	-6.78625
12	3flw	1	12	-6.782042	1.2467362	21	12	11.52257	-89.6043	-11.5896	-35.9184	-6.78204
7	4ehz	1	7	-6.777431	1.6751717	1 2 24 6	[[4,7,11,3,8,14,25,2,13,26],[1,6,5,4,3,2],[4,7,8,9,10,5],[25,4,7,8,9,10,11,30,15,19]]	5.975885	-90.0369	-12.3346	-39.4604	-6.77743
bulit-	4bib	1	1	-6.775487	0.97490472	1.1058686	14 1 2 3 4 9 13	[[30,36,3	-24.8162	-91.703	-10.8828	-38.861
12	3v6s	1	12	-6.773796	1.0929025	17 5	[[4,7,8,9,10,5,15,20,19,18,17,16],[15,20,19,18,17,16]]	12.92774	-68.6816	-14.0678	-40.0284	-6.7738
7	3v6s	1	7	-6.771868	0.98954862	17 22 23 14	[[15,20,19,18,17,16],11,11,[8,20]]	10.00462	-85.0614	-13.086	-33.7963	-6.77187
7	3v6s	1	7	-6.763874	2.3253849	17 7 8 14 5	[[4,7,8,9,10,5],21,21,8,[15,20,19,18,17,16]]	10.15328	-90.4547	-12.4906	-42.2168	-6.76387
12	3v6s	1	12	-6.762381	0.94418257	14 5	[8,[15,20,19,18,17,16]]	13.32239	-76.5735	-14.4996	-40.7735	-6.76238
7	4nyi	1	7	-6.761719	1.2243096	6	[[23,36,37,12,34,22,32,35,1,13]]	9.684947	-83.0139	-14.6771	-41.9022	-5.46172
7	3v6s	1	7	-6.750516	1.4540621	7 8 14 9 10 5	[21,21,8,14,14,[15,20,19,18,17,16]]	8.206195	-102.696	-13.9986	-44.1186	-6.75052
bulit-	4nyi	1	1	-6.747789	1.3553718	1.8454504	1 3 4 5 7 8 11 12 13 14 39 17 19 20 21 22 23 24 25 40 41 43 29 30 31 32 33 42 34 35 36 37 38	[3,3,3,1	-871.827	-67.6201	-34.0911	-181.065
3	4nyi	1	3	-6.731489	2.0260532	6 17 18	[[20,21,31,32,33,17,18,29,16,15],[14,19,18,17,16,15],[14,19,18,17,16,15]]	9.437621	-91.9436	-16.151	-21.6069	-5.43149
12	4nyi	1	12	-6.726321	2.5369918	6	[[12,26,33,1,13,22,32,2,34,6]]	13.22859	-87.2091	-14.4465	-40.4687	-5.42632
12	3flw	1	12	-6.725523	0.87017453	8 9 30 33 10	[11,11,12,12,11]	15.23576	-94.9154	-11.2036	-38.8449	-6.72552
7	4nyi	1	7	-6.721903	0.99528039	6	[[23,36,37,12,34,22,32,35,1,13]]	10.65049	-85.8641	-14.6621	-46.5347	-5.4219
bulit-	4nyi	1	1	-6.718116	1.183099	2.0487707	3 4 11 12 13 14 17 18 19 20 22 23 26 27 40 41 29 30 31 32 33 42 34 35 36 37 38	[4,4,4,4,	-862.562	-120.037	-38.8018	-181.315
3	4nyi	1	3	-6.706736	1.1204723	4 6 7 11	[11,[12,25,1,2,22,6,3,13,26,4],12,12]	-13.085	-99.8976	-13.3672	-41.3837	-5.40674
3	4bib	1	3	-6.705457	0.94828147	3 19	[[10,23,19,30,9,14,15,5,8,16],[14,19,18,17,16,15]]	-14.382	-83.5392	-13.0339	-33.7654	-6.70546
7	4ehz	1	7	-6.70534	1.8128953	1 2 24 6	[[23,37,12,35,36,1,5,6,10,24],[1,6,5,4,3,2],[4,7,8,9,10,5],[9,10,15,16,17,18,28,21,31,19]]	12.28643	-102.103	-12.7533	-39.9355	-6.70534
12	3flw	1	12	-6.695149	1.6652867		[]	12.67343	-89.5514	-12.15	-33.9395	-6.69515
7	3v6s	1	7	-6.67961	1.4881315	17 7 14 5	[[4,7,8,9,10,5,4,7,8,9,10,5],21,8,[15,20,19,18,17,16]]	10.75014	-80.5694	-12.5769	-41.2656	-6.67961
9	4nyi	1	9	-6.675102	1.0585878	6 26	[[12,10,38,9,37,11,36,40,31,32],[22,25]]	23.57015	-97.205	-13.6941	-55.611	-5.3751
7	3flw	1	7	-6.674876	1.9918466	19 20	[21,21]	11.15035	-82.9808	-13.4972	-41.8414	-6.67488
20	3flw	1	20	-6.66489	2.1629295	3 21 4	[[4,7,8,9,10,5,4,7,8,9,10,5],31,29]	53.45403	-105.194	-12.9257	-37.6353	-6.66489
3	4bib	1	3	-6.66389	1.5518619	1 3 19	[[1,6,5,4,3,2,1,6,5,4,3,2],[7,8,11,5,9,10,4,6,14,15],[4,7,8,9,10,5,4,7,8,9,10,5]]	-15.4695	-80.1481	-11.9799	-36.9786	-6.66389
12	4nyi	1	12	-6.662401	1.0441017	6	[[12,26,22,32,1,13,33,34,2,6]]	12.90407	-73.5969	-14.5009	-41.9853	-5.3624
16	4ehz	1	16	-6.653794	2.4731913	2 12 13 23 30 6	[[1,6,5,4,3,2],19,19,18,21,[21,31,27,15,16,14,22,32]]	4.769875	-96.3337	-13.7551	-38.2208	-6.65379
20	3flw	1	20	-6.649158	1.1936612	12 18	[18,18]	46.98443	-92.8823	-13.1496	-37.7496	-6.64916
9	4nyi	1	9	-6.646441	1.2165644	2 6 26	[[41,13,42,26,35,43,1,6,27,53],[31,12,37,9,10,7,32,4,30,33],[22,25]]	29.71186	-100.77	-13.2833	-59.4573	-5.34644
7	3flw	1	7	-6.642991	0.83935869	8 9	[11,11]	7.237696	-107.015	-11.8658	-33.0271	-6.64299
3	3flw	1	3	-6.639086	1.0570241	8	11	-14.2774	-76.6067	-11.4228	-32.7499	-6.63909
bulit-	4ehz	1	1	-6.637328	3.6807992	0.87304068	8 12 6	[[32,34,1	27.934	-84.9403	-14.6543	-30.1593
17	4nyi	1	17	-6.634863	1.7028325	6	[[16,29,15,1,2,28,6,3,17,4]]	-27.7256	-67.521	-11.6815	-19.3774	-5.33486

16	4bib	1	16	-6.625709	0.77840948	1 3 42 45 8 16	[[1,6,5,4,3,2],[27,7,8,9,10,20,30,4,5,6],20,20,[4,5,2,3,6,7,8,9,10,17],[11,16,15,14,13,12]]	6.358019	-113.775	-14.3009	-39.6415	-6.62571
16	4bib	1	16	-6.580311	1.5589663	1 22 8	[[11,16,15,14,13,12],19,[11,12,13,9,10,14,15,16,21,22]]	3.478776	-113.343	-13.6327	-40.1424	-6.58031
3	4nyi	1	3	-6.57907	1.0075405	6 7 11	[[13,3,11,26,2,4,22,7,8,5],13,13]	-15.8642	-89.1848	-14.0391	-14.1231	-5.27907
14	4bib	1	14	-6.577346	0.84478939	3 20 21	[[19,28,12,13,14,31,32,4,10,11],9,9]	-12.4439	-82.88	-10.0083	-23.4591	-6.57735
20	3flw	1	20	-6.575695	1.5324581	34 12 18 6 13 14	[18,18,18,27,30,30]	66.1789	-105.233	-13.5521	-41.9843	-6.5757
3	4bib	1	3	-6.57542	0.57533354	20 21	[13,13]	-13.5619	-87.7861	-11.4995	-31.2542	-6.57542
3	4bib	1	3	-6.563292	0.64646405	3 19 22 20 21 23 24	[[8,24,9,14,19,30,7,10,15,16],[14,19,18,17,16,15],13,[2,12],12,[4,7,8,9,10,5],[4,7,8,9,10,5],[1,2,3,4,5,6,12,22,23,25]]	-18.8418	-92.2836	-12.4562	-39.1093	-6.56329
7	3flw	1	7	-6.562856	1.0956589	1 8	[[32,22,33,1,12,23,35,36,2,6],11]	9.484345	-108.181	-11.9102	-33.2898	-6.56286
4	4nyi	1	4	-6.562535	1.6230849	2 27 3 17 16 26	[[35,50,12,36,30,13,41,14,22,43],30,[27,30,40,47,50,21,23,48,18,26],[4,7,8,9,10,5],[13,36,43,12,14,20,35,22,42,41],28]	55.88936	-66.4187	-14.7589	-47.9007	-5.26254
4	4nyi	1	4	-6.556691	1.7887219	2 3 17 18 26	[[51,37,38,15,16,31,44,22,41,43],[44,45,46,51,18,24,25,26,1,6],[4,7,8,9,10,5],[4,7,8,9,10,5],29]	52.85204	-92.5274	-18.2604	-48.9091	-5.25669
14	3flw	1	14	-6.556687	1.2138177	21 22	[9,9]	-13.6695	-65.2768	-10.7643	-29.683	-6.55669
16	4bib	1	16	-6.554808	1.132857	19 42 45 48 31 14 23	[[4,7,8,9,10,5],21,21,17,19,17,[11,16,15,14,13,12],22,22]	7.692291	-113.95	-13.4516	-42.066	-6.55481
3	4nyi	1	3	-6.550805	2.0558584	8 6 17 7	[[14,19,18,17,16,15],[12,25,1,22,2,3,26,5,6,23],[4,7,8,9,10,5],12]	-13.8301	-96.1137	-17.4784	-39.4562	-5.25081
20	3flw	1	20	-6.542504	1.661037	31 32 12 18 13 14	[29,29,18,18,[27,30],30]	50.54865	-127.205	-15.0134	-43.2487	-6.5425
12	4ehz	1	12	-6.540462	2.0709965	1 2 19 6	[[12,1,5,6,10,24,26,2,3,4],[1,6,5,4,3,2],12,[9,10,16,24,28,29,17,18,21,23]]	12.61302	-87.7326	-12.2033	-38.6967	-6.54046
14	4ehz	1	14	-6.522544	1.8824576	1	[[7,24,26,10,11,20,5,8,25,1]]	-13.2246	-80.7409	-10.8752	-31.2851	-6.52254
16	3flw	1	16	-6.521692	0.74215728	8 9 10 11	[17,17,17,17]	9.510285	-113.472	-12.9438	-40.0108	-6.52169
20	3flw	1	20	-6.52101	2.8232636	19 20 12 18	[30,30,27,27]	55.24772	-100.146	-12.4468	-34.6104	-6.52101
3	4bib	1	3	-6.520252	1.479017	1 19	[[1,6,5,4,3,2,1,6,5,4,3,2],[4,7,8,9,10,5]]	-17.187	-99.5725	-11.5315	-37.1366	-6.52025
9	4nyi	1	9	-6.512268	1.9741092	6 26	[[31,38,7,32,11,35,39,40,33,4],[22,24,25]]	19.39583	-81.3169	-16.404	-81.3497	-5.21227
12	4ehz	1	12	-6.511855	2.0223637	1 2 26 31 20	[[10,20,31,15,18,19,30,35,6,24],[4,7,8,9,10,5,4,7,8,9,10,5],11,11,[37,18,21,19,20,23,30,36,15,16]]	11.806	-91.7689	-11.931	-34.4878	-6.51186
7	3flw	1	7	-6.509216	1.1985742	8 9	[11,11]	8.224216	-104.859	-11.3225	-33.9676	-6.50922
12	4ehz	1	12	-6.50646	1.089875	24 6	[[4,7,8,9,10,5],[7,8,9,11,15,25,28,16,17,29]]	10.07637	-97.9178	-12.128	-38.2957	-6.50646
12	4ehz	1	12	-6.504011	2.2036083	1 2 24 25 33 19 22 6	[[1,5,6,24,10,12,26,31,2,3],[1,6,5,4,3,2],[4,7,8,9,10,5],[4,7,8,9,10,5],[18,21,23,35,36,37],12,12,[5,6,9,10,24,31,15,19,20,30]]	10.56728	-98.863	-12.5201	-39.3532	-6.50401
14	3flw	1	14	-6.502493	1.310066	21 22	[9,9]	-14.6911	-74.4728	-10.3777	-33.9195	-6.50249
3	4ehz	1	3	-6.488726	0.89696234	2 23	[[1,6,5,4,3,2],12]	-14.3202	-86.561	-11.7041	-25.3756	-6.48873
16	4ehz	1	16	-6.485415	1.5127779	1 21 4 14 16	[[12,25,11,13,14,26,10,24,5,9],[1,6,5,4,3,2],19,18,18]	8.712336	-93.7101	-14.1723	-41.2613	-6.48541
9	4nyi	1	9	-6.484311	2.9658206	2 6 15 41 26	[[26,13,41,42,1,6,27,29,37,43],[32,7,31,35,4,30,33,5,8,34],12,12,25]	8.224733	-85.4724	-13.8962	-34.3354	-5.18431
3	4ehz	1	3	-6.479238	1.9926494	2 24 25 12 13 6	[[1,6,5,4,3,2],[14,19,18,17,16,15],[14,19,18,17,16,15],12,12,[27,28,14,15,16,17,20,21,32,33]]	-17.4865	-84.6983	-11.9695	-36.9332	-6.47924
7	3flw	1	7	-6.478345	1.1461245	10 13	[14,14]	10.73597	-82.9259	-11.1885	-37.1593	-6.47835
3	4ehz	1	3	-6.477994	1.8086761	2 24 12 13 23 6	[[1,6,5,4,3,2],[14,19,18,17,16,15],13,13,12,[15,16,27,28,31,17,20,21,32,33]]	-16.9282	-94.0812	-11.5394	-35.6703	-6.47799
12	4ehz	1	12	-6.472188	2.6064305	1 2 24 25 50 9 19 22	[[1,5,6,10,24,2,4,12,26,28],[1,6,5,4,3,2],[4,7,8,9,10,5],[4,7,8,9,10,5],[16,17,28,29,15,18,37,21,23,19],[7,8,11,25,4,9,3,14,27,31],12,12,[5,9,10,24,16,28,15,17,18,20]]	12.52081	-86.0453	-12.2949	-39.4437	-6.47219
16	3v6s	1	16	-6.462997	2.0400023	7 14 18 19	[19,17,18,18]	5.078489	-90.1172	-13.2191	-32.9862	-6.463
16	3v6s	1	16	-6.45717	1.5151461	7 14 18 19	[19,17,18,18]	4.944116	-80.7107	-13.6828	-35.0233	-6.45717

16	3flw	1	16	-6.455411	0.83098775	16	21	7.015657	-84.0757	-13.1581	-29.4382	-6.45541
12	4nyi	1	12	-6.451715	2.2094517	6	[[26,12,22,32,34,33,13,1,2,6]]	13.36151	-78.0597	-13.8352	-43.0071	-5.15172
16	4ehz	1	16	-6.431804	1.1968884	1 21 4 23 14 16	[[16,27,31,15,21,10,24,5,6,9],[1,6,5,4,3,2],19,20,18,18]	9.288373	-114.991	-14.3734	-39.5758	-6.4318
14	4ehz	1	14	-6.427092	2.3349063	1 24 7 8	[[20,25,8,21,5,6,1,2,7,9],[1,6,5,4,3,2],17,17]	-12.9166	-75.2352	-11.5526	-32.3889	-6.42709
16	4ehz	1	16	-6.409009	1.059263	2 12 13 14	[[1,6,5,4,3,2],18,18,21]	8.118939	-110.557	-14.1011	-37.4507	-6.40901
3	3v6s	1	3	-6.401907	1.890959		[]	-16.6738	-90.2332	-12.2797	-34.5197	-6.40191
3	4ehz	1	3	-6.395651	0.87578201	1 2	[[6,23,1,12,5,10,30,2,3,4],[1,6,5,4,3,2]]	-14.254	-98.2777	-12.1526	-30.7843	-6.39565
14	4nyi	1	14	-6.39544	1.0772618	27 53 6 26	[17,17,[27,28,30,13,14,31,12,29,32,7],16]	-14.9588	-81.897	-12.0643	-45.5041	-5.09544
14	4ehz	1	14	-6.393305	2.6410122	1 2	[[8,20,24,21,22,10,25,32,5,7],[1,6,5,4,3,2,1,6,5,4,3,2]]	-11.5377	-70.3754	-11.0788	-29.159	-6.39331
14	4nyi	1	14	-6.391869	1.9268461	6 26	[[29,28,13,31,11,12,30,14,26,27],16]	-12.8133	-59.948	-13.0182	-38.9317	-5.09187
14	4nyi	1	14	-6.39029	1.7893682	6 26	[[29,27,13,31,12,30,11,14,26,28],16]	-12.8522	-61.2733	-12.158	-38.7802	-5.09029
14	3v6s	1	14	-6.387633	1.9551994		[]	-14.0984	-78.9156	-12.7848	-30.5369	-6.38763
3	4ehz	1	3	-6.37865	0.82670557	1 21 4 5 14	[[8,15,24,27,14,16,28,7,9,11],[1,6,5,4,3,2],12,12,13]	-16.7638	-97.4485	-12.0488	-35.0156	-6.37865
16	4ehz	1	16	-6.372937	0.57717043	1 2 7 8 14 16	[[6,24,1,18,5,10,25,2,3,4],[1,6,5,4,3,2],21,21,20,20]	6.064731	-106.809	-13.6102	-34.9079	-6.37294
14	4ehz	1	14	-6.360415	1.3279777	1 2	[[15,17,33,4,16,19,31,3,2,5],[1,6,5,4,3,2]]	-13.4195	-72.3753	-10.5138	-28.1257	-6.36042
14	4ehz	1	14	-6.358866	1.2457964	1 2	[[15,16,17,19,26,33,3,4,28,2],[1,6,5,4,3,2]]	-11.087	-82.6556	-11.4545	-31.2753	-6.35887
14	3flw	1	14	-6.339597	1.8265811	13 14	[9,9]	-16.897	-66.7624	-10.7089	-36.5964	-6.3396
3	3v6s	1	3	-6.338934	1.0177253	14 9 10	[8,13,13]	-13.9261	-84.6254	-14.1703	-39.0418	-6.33893
14	3flw	1	14	-6.336196	3.4676986		[]	-12.9146	-63.4916	-10.6536	-27.8077	-6.3362
3	3v6s	1	3	-6.330311	2.9534991	9 10 5	[12,12,[14,19,18,17,16,15]]	-14.9171	-79.2499	-12.5263	-38.2956	-6.33031
14	3flw	1	14	-6.321473	2.0135663		[]	-14.5959	-73.2598	-10.3681	-32.3806	-6.32147
14	4bib	1	14	-6.321396	1.7762539	3 22 6	[[24,30,11,12,13,26,27,29,5,10],9,[1,2,3,4,15,16,17,18,19,33]]	-15.1053	-65.907	-10.1913	-28.3836	-6.3214
14	3v6s	1	14	-6.320687	1.1096145	14 24	[9,9]	-15.0457	-62.7425	-10.4575	-31.3228	-6.32069
16	3v6s	1	16	-6.318814	1.8934186	17 31 5	[[4,7,8,9,10,5],19,[1,6,5,4,3,2]]	8.464665	-78.2322	-13.5146	-33.5457	-6.31881
19	4nyi	1	19	-6.317537	0.93554479	6 57 58	[[11,12,13,23,3,14,2,22,4,1],12,12]	-6.70443	-61.9528	-11.9889	-25.0446	-5.01754
16	3v6s	1	16	-6.300639	1.4736158		[]	3.096311	-75.6057	-13.2685	-32.4789	-6.30064
16	3v6s	1	16	-6.280108	1.7521101	17 14 24 5	[[11,16,15,14,13,12],[16,21],21,[11,16,15,14,13,12]]	10.11972	-93.9329	-13.4586	-37.2064	-6.28011
3	3v6s	1	3	-6.277241	1.2750149	17 5	[[4,7,8,9,10,5,14,19,18,17,16,15],[14,19,18,17,16,15]]	-14.3287	-76.6055	-12.2816	-37.6246	-6.27724
3	3v6s	1	3	-6.260803	3.0516505	7 14 18 19	[13,11,12,12]	-16.8231	-75.4977	-11.8907	-35.6425	-6.2608
16	3flw	1	16	-6.242453	1.69866	1 31 8 9 3 10 11	[[21,13,14,15,22,26,32,16,27,31],21,17,17,[11,16,15,14,13,12],17,17]	6.734031	-94.9006	-13.2292	-35.4282	-6.24245
3	3flw	1	3	-6.24211	2.1620133	8 9 3 10 11	[11,11,[14,19,18,17,16,15],11,11]	-14.4449	-75.3258	-11.9969	-35.9218	-6.24211
14	4bib	1	14	-6.238484	1.2505776	1 8	[[1,6,5,4,3,2],[33,28,31,1,2,3,4,5,6,7]]	-15.3738	-74.1084	-10.4722	-22.9864	-6.23848
8	4nyi	1	8	-6.219485	1.4603475	1 6	[[1,6,5,4,3,2],[13,19,2,12,1,18,3,14,4,5]]	-6.89766	-53.3872	-11.2206	-19.3983	-4.91949
14	4bib	1	14	-6.203831	1.1464418	3 22 20 21 6	[[7,14,20,24,30,32,21,31,5,10],9,17,17,[1,2,3,4,5,15,16,17,18,19]]	-11.4224	-76.1725	-10.1807	-29.1902	-6.20383
3	3flw	1	3	-6.2015	1.1585062	21 22	[12,12]	-15.5749	-82.8048	-11.0555	-32.4678	-6.2015
12	4nyi	1	12	-6.189012	1.8643075	6	[[12,26,1,13,22,33,34,2,3,5]]	14.38111	-75.4533	-14.1385	-35.3699	-4.88901
14	4bib	1	14	-6.177824	2.5622969	3 19 6	[[1,2,3,9,18,23,5,6,7,21],[1,6,5,4,3,2,1,6,5,4,3,2],[11,12,13,14,19,24,28,29,30,31]]	-13.1424	-71.5123	-10.0948	-29.2735	-6.17782
14	3v6s	1	14	-6.175432	1.1872635	13 36 17 22 23 14	[9,9,[1,6,5,4,3,2],16,16,4]	-14.5357	-62.7214	-10.8218	-27.077	-6.17543
14	3v6s	1	14	-6.169746	1.7158281	6	17	-13.6527	-67.339	-11.9147	-27.6501	-6.16975
14	3v6s	1	14	-6.166646	1.1550249	14	2	-12.7776	-65.4626	-11.1887	-30.4774	-6.16665
3	3flw	1	3	-6.142944	2.094275	4	12	-14.2524	-80.8952	-11.015	-35.4754	-6.14294
3	3flw	1	3	-6.131932	2.0237064	8 9 10 21 22	[11,11,11,13,13]	-12.1346	-87.1721	-11.0621	-35.0941	-6.13193
17	4bib	1	17	-5.969632	1.7928083	3 20 21	[[13,14,20,23,26,8,11,12,25,7],15,15]	-28.1879	-65.4696	-11.4009	-25.8357	-5.96963
17	4bib	1	17	-5.954059	1.1826653	3 20 21 16	[[8,13,19,20,21,27,7,9,10,14],16,16,[1,6,5,4,3,2]]	-27.2684	-62.5065	-10.5571	-23.5372	-5.95406

16	3flw	1	16	-5.91647	2.6651578	15	[[11,16,15,14,13,12]]	5.599943 -88.9814 -12.8514 -36.0451 -5.91647
16	3flw	1	16	-5.91147	1.3905249		[]	5.519156 -90.4346 -13.9308 -34.0577 -5.91147
17	4nyi	1	17	-5.900956	2.3516927	8 4 5 6	[[1,6,5,4,3,2],14,14,[12,14,26,27,13,23,8,9,10,20]]	-26.0063 -41.302 -13.642 -39.2631 -4.60096
19	3flw	1	19	-5.871233	0.68459982		[]	-1.2561 -65.237 -9.13994 -24.3375 -5.87123
17	4nyi	1	17	-5.870404	1.3851827	8 4 5 6	[[1,6,5,4,3,2],14,14,[26,12,14,13,23,8,11,20,25,9]]	-30.7766 -72.5196 -12.3689 -39.5298 -4.5704
19	3flw	1	19	-5.862255	2.376754		[]	-5.63012 -61.324 -9.21259 -25.0689 -5.86226
17	3v6s	1	17	-5.848464	1.065747	13 36	[15,15]	-28.8485 -54.8587 -10.8622 -25.7018 -5.84846
17	4nyi	1	17	-5.836688	1.660758	4 5 6	[14,14,[26,12,11,13,14,24,25,27,23,4]]	-29.3248 -59.2945 -15.3383 -31.5067 -4.53669
17	4ehz	1	17	-5.832331	1.1616936	26 31	[14,14]	-28.5253 -69.7374 -9.75598 -29.3179 -5.83233
17	4bib	1	17	-5.81999	1.000859	19 14 33 8 4 5 6	[[1,6,5,4,3,2],14,14,[25,26,23,4,5,7,8,9,10,11],[15,15],[15,15],[18,6,22,28,1,15]]	-29.2449 -70.9247 -11.5233 -28.8829 -5.81999
17	4bib	1	17	-5.799228	1.2465296	8	[[25,5,10,23,3,4,6,7,8,11]]	-28.9969 -58.5074 -10.0896 -26.0621 -5.79923
19	3flw	1	19	-5.792639	1.1672655	13	5	-5.09767 -60.1252 -9.1112 -24.953 -5.79264
17	4bib	1	17	-5.76709	1.4408942	19 12 8	[[1,6,5,4,3,2],15,[21,1,2,3,4,5,6,15,16,17]]	-30.0598 -81.7889 -10.1302 -28.7084 -5.76709
17	4ehz	1	17	-5.758651	1.4464055	1 2 3 19 22	[[21,1,15,5,6,18,22,10,28,2],[1,6,5,4,3,2],[1,6,5,4,3,2],15,15]	-27.4749 -52.4918 -9.74181 -25.9393 -5.75865
17	3v6s	1	17	-5.736975	1.246447	9 10	[16,16]	-29.2652 -55.3599 -10.2516 -28.6517 -5.73697
17	3flw	1	17	-5.719678	2.221005		[]	-27.5723 -47.3028 -9.68212 -27.2056 -5.71968
19	3flw	1	19	-5.715515	0.97873068		[]	-4.91602 -60.0058 -8.89387 -26.536 -5.71552
17	3v6s	1	17	-5.715152	1.4887618		[]	-30.2757 -68.3821 -10.1951 -25.6739 -5.71515
19	3flw	1	19	-5.706616	0.48637089		[]	-5.09427 -64.4548 -9.40717 -24.4346 -5.70662
8	3flw	1	8	-5.636545	1.1950523	21 22	[13,13]	-6.29069 -80.2055 -9.648 -24.9052 -5.63654
17	3v6s	1	17	-5.632562	1.8153104	13	16	-30.1098 -75.3519 -10.2068 -29.1259 -5.63256
17	4ehz	1	17	-5.60928	2.7807593	1 2	[[25,16,29,3,11,17,24,2,1,4],[1,6,5,4,3,2]]	-28.76 -59.7849 -10.136 -25.0287 -5.60928
19	4nyi	1	19	-5.599302	1.5795373	59 26 54	[[1,6,5,4,3,2],12,12]	-4.25491 -71.5539 -12.2244 -41.0029 -4.2993
19	4ehz	1	19	-5.59741	3.6419899	2 12	[[1,6,5,4,3,2],13]	-6.05771 -70.4212 -9.52245 -27.3111 -5.59741
17	4ehz	1	17	-5.597343	2.2905462	1	[[3,17,19,2,4,16,29,1,7,11]]	-28.3858 -66.7698 -9.81142 -24.5914 -5.59734
17	4ehz	1	17	-5.592792	1.5953295	2	[[1,6,5,4,3,2]]	-28.6636 -65.6917 -9.65653 -27.0529 -5.59279
17	3v6s	1	17	-5.557982	1.6305746	7	15	-25.9156 -50.2203 -10.8593 -27.1507 -5.55798
19	4bib	1	19	-5.519432	0.77646732	40 53	[12,12]	-4.28547 -68.9114 -9.37951 -18.168 -5.51943
17	3flw	1	17	-5.471204	1.0892928	30	16	-27.8275 -64.5485 -9.45563 -24.248 -5.4712
19	3v6s	1	19	-5.437053	1.0060139	31	8	-4.94702 -60.1572 -9.83126 -25.3465 -5.43705
19	3v6s	1	19	-5.416422	1.4928786		[]	-7.04176 -60.2442 -9.50815 -25.7174 -5.41642
17	4nyi	1	17	-5.401553	1.5556438		[]	-25.011 -64.7819 -13.9892 -29.3105 -4.10155
19	3v6s	1	19	-5.394178	1.3095936	40 41 5	[12,12,[1,6,5,4,3,2]]	-6.35978 -59.8292 -9.30869 -23.0811 -5.39418
8	4ehz	1	8	-5.388821	0.59154868	1 2 23	[[12,18,15,6,1,2,3,4,5,13],[1,6,5,4,3,2],13]	-6.95233 -59.0886 -9.47723 -24.1209 -5.38882
19	4nyi	1	19	-5.384545	2.7127831	26	12	-3.55329 -80.2156 -13.7457 -39.9981 -4.08454
19	4bib	1	19	-5.371798	1.0855311	3	[[15,16,5,17,19,4,6,7,8,18]]	-5.63404 -64.3797 -9.37258 -22.9455 -5.3718
19	4bib	1	19	-5.353386	0.76617813	4 6	[13,[1,2,3,4,5,6,7,8,15,16]]	-4.77475 -66.0862 -8.96351 -20.247 -5.35339
8	4ehz	1	8	-5.334032	1.3545846	1 2 4 23	[[13,19,14,2,3,1,4,6,7,12],[1,6,5,4,3,2],9,12]	-6.79491 -51.5192 -9.91231 -24.004 -5.33403
19	3v6s	1	19	-5.329889	2.7285626	17 14	[[1,6,5,4,3,2],2]	-6.2609 -59.7153 -9.31365 -26.7151 -5.32989
19	4bib	1	19	-5.327627	0.81725866		[]	-2.62347 -62.0647 -9.84785 -16.0245 -5.32763
19	3v6s	1	19	-5.299749	1.0005615	35	13	-5.31512 -74.9649 -9.45405 -26.6412 -5.29975
19	4bib	1	19	-5.270317	1.3698238	3 20 21 6	[[19,9,10,21,22,1,20,6,7,8],13,13,[1,2,3,4,11,12,13,14,15,23]]	-4.98872 -66.8115 -9.80039 -23.0509 -5.27032
19	4ehz	1	19	-5.243303	1.515618	1 9	[[11,12,2,3,13,14,1,20,4,5],[16,4,5,15,6,7,8,19,1,17]]	-7.07597 -64.5253 -8.96419 -23.4378 -5.2433
19	4nyi	1	19	-5.223949	1.6888561	26	12	-4.74526 -66.6465 -11.7433 -39.3394 -3.92395
19	4ehz	1	19	-5.221115	1.2666976	1 2 26 31	[[22,10,1,9,21,20,2,14,3,6],[1,6,5,4,3,2],12,12]	-7.02617 -62.0852 -9.06687 -27.308 -5.22111
8	4bib	1	8	-5.216312	0.73064351	20 21	[13,13]	-5.87912 -71.3695 -10.7173 -21.391 -5.21631
19	4nyi	1	19	-5.180684	1.5093501	26	12	-6.14247 -70.9656 -12.3374 -17.0911 -3.88068
8	4ehz	1	8	-5.168692	1.5658461	2 26	[[1,6,5,4,3,2],12]	-6.81114 -71.1459 -10.0555 -25.0034 -5.16869

17	3flw	1	17	-5.157734	1.7215863	13 14	[16,16]	-30.0009	-55.7774	-9.76542	-26.086	-5.15773
19	4ehz	1	19	-5.156723	1.0157572	26	13	-5.51835	-77.1649	-9.03665	-27.0267	-5.15672
8	3flw	1	8	-5.154935	1.5173743	28 29	[11,11]	-6.54102	-64.2278	-9.61964	-22.7705	-5.15494
19	4ehz	1	19	-5.153882	1.3519725	26 31	[12,12]	-5.11147	-65.0488	-8.97498	-25.841	-5.15388
17	3flw	1	17	-5.104636	1.2477417	8 9 10 11 16	[14,14,14,14,16]	-28.4311	-78.7351	-10.1495	-25.2799	-5.10464
15	3flw	1	15	-5.086645	1.8913363	8 9 30 33	[11,11,7,7]	-26.9421	-67.4176	-11.5434	-21.8162	-5.08664
15	3flw	1	15	-5.083914	1.2338367	8 9 30 10 11	[11,11,7,11,11]	-27.6199	-58.2964	-9.82294	-24.7722	-5.08391
17	3flw	1	17	-5.079452	2.5100403		[]	-29.937	-76.9653	-9.59613	-21.2028	-5.07945
15	3flw	1	15	-5.067165	2.5561211		[]	-27.2093	-68.9183	-10.3846	-22.4527	-5.06716
8	4ehz	1	8	-5.028329	0.96133989	9 21 43 4 12 13 26	[[8,9,10,16,17,5,4,7,11,1],[4,7,8,9,10,5],[4,7,8,9,10,5]],11,13,13,13]	-6.74289	-58.4825	-9.60285	-24.5533	-5.02833
8	3v6s	1	8	-5.020923	2.0341885	17 4 25 26	[[1,6,5,4,3,2],11,11,[4,7,8,9,10,5]]	-6.89839	-56.7416	-11.0849	-21.5781	-5.02092
8	4bib	1	8	-5.0153	1.7498608	3 20 21 6	[[11,7,8,9,10,16,17,4,5],12,12,[1,2,3,4,5,6,12,13,14,15]]	-6.10015	-68.0874	-10.5002	-21.6657	-5.0153
8	4ehz	1	8	-4.996341	1.7422122	14 16	[13,13]	-7.15385	-63.8956	-9.59681	-24.8848	-4.99634
15	3flw	1	15	-4.984514	1.2009171	21 22	[7,7]	-26.5995	-58.1366	-9.64579	-21.0417	-4.98451
8	3v6s	1	8	-4.973851	2.1776745	17 14 26	[[4,7,8,9,10,5,1,6,5,4,3,2],6,[1,6,5,4,3,2]]	-6.95897	-55.8773	-10.2997	-24.2097	-4.97385
8	3flw	1	8	-4.972781	0.97598541	13 14	[13,13]	-6.99524	-76.8164	-9.35909	-25.5994	-4.97278
15	3flw	1	15	-4.971583	1.5141188	21 22	[8,8]	-27.5075	-52.6589	-10.0733	-23.7561	-4.97158
15	4nyi	1	15	-4.953846	1.5020658	26 54	[11,11]	-27.0865	-73.2727	-12.4196	-26.8099	-3.65385
8	3v6s	1	8	-4.925707	1.0628302	17 14	[[4,7,8,9,10,5,1,6,5,4,3,2],3]	-7.0729	-60.2407	-9.51961	-25.67	-4.92571
8	4nyi	1	8	-4.90707	0.92954588	6	[[8,9,16,7,10,11,17,5]]	-6.04879	-59.8758	-13.032	-24.3539	-3.60707
8	4nyi	1	8	-4.884285	1.5342505	39 40 8 26	[13,13,[4,7,8,9,10,5],11]	-6.03547	-64.062	-11.4591	-20.9966	-3.58429
15	4ehz	1	15	-4.848695	0.89921564	2 12 26	[[1,6,5,4,3,2],8,8]	-27.7956	-70.1584	-9.51902	-20.993	-4.8487
8	3v6s	1	8	-4.834397	1.0843642	4 25	[11,11]	-7.133	-55.1617	-9.56694	-24.3851	-4.8344
15	4nyi	1	15	-4.812675	2.5838695	55 39 26	[8,7,11]	-27.6121	-60.9675	-12.2472	-39.5353	-3.51267
15	4ehz	1	15	-4.811939	1.5430428	12 26	[7,7]	-27.7116	-64.4207	-9.49818	-22.6935	-4.81194
8	4bib	1	8	-4.809781	2.2454469	3 19 42 12 13	[[6,10,15,17,1,4,5,2,3,7],[1,6,5,4,3,2,4,7,8,9,10,5],11,13,13]	-7.11542	-79.0191	-9.46909	-25.1092	-4.80978
8	4bib	1	8	-4.808991	1.8564057	19 42 45 14 33	[[4,7,8,9,10,5,1,6,5,4,3,2,1,6,5,4,3,2],13,13,11,11]	-6.65716	-68.1733	-10.1656	-25.1169	-4.80899
8	4bib	1	8	-4.805298	0.91802114	3 19 42 12 13	[[6,10,15,17,1,4,5,2,3,7],[1,6,5,4,3,2,4,7,8,9,10,5,4,7,8,9,10,5]],11,13,13]	-7.1177	-83.766	-9.88906	-25.126	-4.8053
15	4nyi	1	15	-4.798666	2.2953424	39 27 53 8 37	[10,8,8,[1,6,5,4,3,2],7]	-29.2401	-74.1302	-12.288	-36.3074	-3.49867
8	3v6s	1	8	-4.7849	0.66715664	3 7 8	[[4,7,8,9,10,5],13,13]	-6.74143	-53.4407	-10.9307	-21.6149	-4.7849
15	4bib	1	15	-4.755419	2.3009181	19 12 13 14 33	[[1,6,5,4,3,2],8,8,11,11]	-27.6422	-70.2642	-9.36258	-22.1512	-4.75542
15	4bib	1	15	-4.745464	2.8351159	1 12 13	[[1,6,5,4,3,2],10,10]	-27.7086	-60.8159	-9.42906	-20.293	-4.74546
15	3v6s	1	15	-4.733975	1.20464	13 5 18 19	[7,[1,6,5,4,3,2],8,8]	-26.0393	-55.5905	-9.84838	-20.3552	-4.73397
15	3v6s	1	15	-4.729227	0.81293029	40 41 5	[11,11,[1,6,5,4,3,2]]	-26.9969	-56.09	-9.86623	-17.9331	-4.72923
15	4nyi	1	15	-4.721747	1.6435386	26	11	-26.8308	-82.1239	-12.3501	-16.6046	-3.42175
15	3v6s	1	15	-4.716661	2.559042	32 33 5	[10,10,[1,6,5,4,3,2]]	-27.7352	-58.1855	-10.3935	-17.6211	-4.71666
15	4bib	1	15	-4.705464	1.7172475	20 21 6	[10,10,[1,3,4,5,6,9,10,11,13,14]]	-26.0791	-67.873	-9.68269	-18.3228	-4.70546
15	3v6s	1	15	-4.693799	2.4935639	31 5 18	[8,[1,6,5,4,3,2],7]	-27.5898	-54.702	-10.3782	-17.6577	-4.6938
15	4bib	1	15	-4.68211	1.3603866	3 20 21 4 5	[[11,9,10,14,17,5,6],7,7,[10,10],10]	-27.2126	-60.9792	-10.5705	-22.9488	-4.68211
15	4ehz	1	15	-4.679424	0.62532645	26 31	[11,11]	-27.1674	-60.8214	-9.7225	-21.9524	-4.67942
15	3v6s	1	15	-4.608673	2.2331948	32 33 5 18 19	[10,10,[1,6,5,4,3,2],8,8]	-27.1836	-61.1577	-9.81062	-21.3048	-4.60867
15	4bib	1	15	-4.603613	1.8870831	19 12 13	[[1,6,5,4,3,2],8,8]	-27.6119	-63.8504	-9.69638	-20.8459	-4.60361
15	4ehz	1	15	-4.572426	0.76101917		[]	-27.417	-63.6977	-10.0485	-20.8042	-4.57243
8	4nyi	1	8	-4.542263	1.1047232	26	[[12,13]]	-6.71301	-47.4449	-11.2631	-51.2916	-3.24226
15	4ehz	1	15	-4.541072	2.1823528	1 26 31	[[1,2,7,12,15,3,5,6,4,8],11,11]	-27.5529	-75.8384	-10.3421	-19.2518	-4.54107
8	4nyi	1	8	-4.528099	1.085984	8 37 38	[[1,6,5,4,3,2],12,12]	-4.96088	-61.5162	-11.8078	-31.9913	-3.2281
8	3flw	1	8	-4.454193	3.7694647	13 14	[[6,12],12]	-6.9862	-47.0009	-9.45878	-20.4803	-4.45419
15	4nyi	1	15	-4.42014	2.1615057	27 26	[8,[10,11]]	-27.4108	-75.6992	-12.5012	-40.3946	-3.12014
8	3flw	1	8	-4.397346	1.166218	8 9 10 11 16	[11,11,11,11,12]	-6.69429	-63.8465	-9.30944	-21.5959	-4.39735
